# Anion Recognition with Antimony(III) and Bismuth(III) Triaryl‐Based Pnictogen Bonding Receptors

**DOI:** 10.1002/chem.202201838

**Published:** 2022-10-11

**Authors:** Heike Kuhn, Andrew Docker, Paul D. Beer

**Affiliations:** ^1^ Department of Chemistry University of Oxford Chemistry Research Laboratory Mansfield Road Oxford OX1 3TA UK

**Keywords:** anion recognition, antimony, bismuth, pnictogen bonding, sigma-hole interactions

## Abstract

The synthesis and characterisation of a library of acyclic antimony(III) and bismuth(III) triaryl pnictogen bonding (PnB) receptor systems are reported. In the first‐generation receptor series, quantitative ^1^H NMR chloride titration experiments in THF solvent media reveal halide anion binding potency is intimately correlated with both the electronic‐withdrawing nature of the aryl‐ substituent and the polarisability of the PnB donor. Further extensive anion binding investigations with the most potent Sb‐ and Bi‐based PnB receptors: **1⋅Sb^2CF3^** and **1⋅Bi^2CF3^**, reveal novel selectivity profiles, both displaying Cl^−^ selectivity relative to the heavier halides and, impressively, to a range of highly basic oxoanions. The synthesis and preliminary chloride anion binding studies of a series of novel tripodal tris‐proto‐triazole triaryl Sb(III) and Bi(III) mixed PnB‐HB receptor systems are also described. Whereas parent triphenyl Sb(III) and Bi(III) compounds are incapable of binding Cl^−^ in THF solvent media, the PnB‐triazole HB host systems exhibit notable halide affinity.

## Graphical abstract

**A series of Antimony(III) and Bismuth(III)** pnictogen bonding (PnB) triaryl‐based anion receptors demonstrate PnB anion binding strength is influenced strongly by the electronic withdrawing nature of the aryl substituent and pnictogen atom donor identity. The triaryl PnB receptors display chloride anion selectivity over a range of halides and oxoanions. The synthesis and preliminary chloride anion binding investigations of the first mixed PnB‐HB anion host systems are also described.

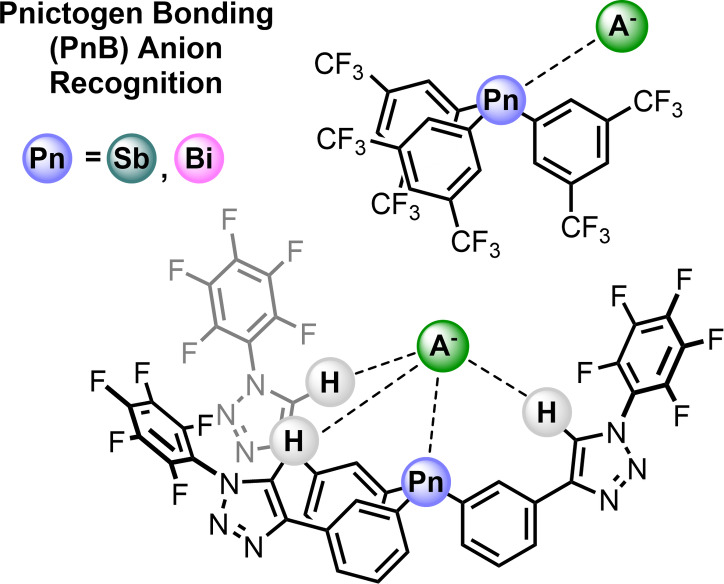

## Introduction

In recent years sigma‐(σ)‐hole interactions, broadly defined as the attractive non‐covalent interactions between an electrophilic region of a main‐group element and a nucleophilic Lewis base, have been the subject of intense interest from the supramolecular chemistry community.[^1^, ^2^, ^3^, ^4^] Halogen bonding (XB) and chalcogen bonding (ChB) in particular, have come to the fore as remarkably powerful additions to the supramolecular chemistry toolbox, with notable achievements in applications such as directed self‐assembly,[^5^, ^6^, ^7^, ^8^, ^9^] materials chemistry,[^10^, ^11^, ^12^, ^13^, ^14^] organocatalysis[^15^, ^16^, ^17^, ^18^] and molecular recognition.[^19^, ^20^, ^21^] Indeed, the considered employment of XB and ChB in host design for the purposes of anion recognition has experienced a surge in activity.[^22^, ^23^, ^24^, ^25^] In particular, the frequently observed superior binding affinities,[^26^, ^27^, ^28^, ^29^, ^30^] contrasting selectivity profiles[^31^, ^32^, ^33^] and improved sensing capabilities[^34^, ^35^, ^36^, ^37^, ^38^] relative to traditional hydrogen bonding (HB) mediated recognition strategies very much constitute a new frontier in host‐guest anion recognition. In contrast, pnictogen bonding (PnB) anion host systems, which employ σ‐hole donors from group 15 of the Periodic Table, are relatively rare. While pioneering examples by Matile and Gabbaï have demonstrated promising potential as organocatalysts[^39^, ^40^, ^41^] and transmembrane anion transporters,[^42^, ^43^] the strategic exploitation of PnB σ‐hole donors in anion receptor design remains underdeveloped.^[44]^

**Figure 1:**
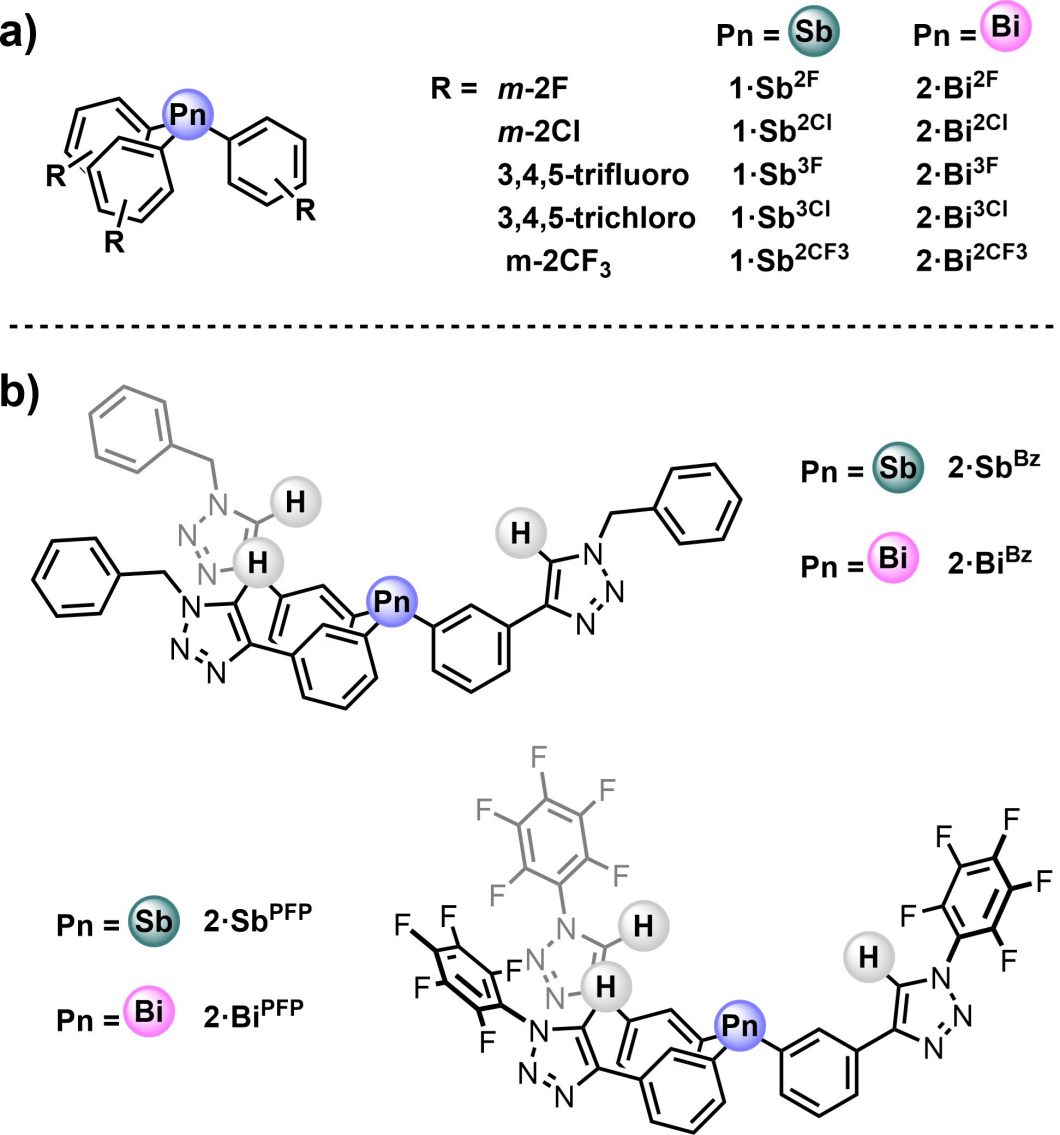
PnB receptors in this study; a) triaryl series **1⋅Sb^R^** and **1⋅Bi^R^**; b) tripodal mixed PnB‐HB series **2⋅Sb^R^** and **2⋅Bi^R^**.

With the aim of understanding how structural and electronic factors influence PnB based anion recognition properties, herein we report the synthesis, characterisation and anion binding studies of a series of antimony(III) and bismuth(III) triaryl acyclic receptor systems. In the first receptor series, **1⋅Sb^R^** and **1⋅Bi^R^** (Figure 1a), extensive quantitative ^1^H NMR anion titration experiments elucidate the effects of the aryl substituent's electron‐withdrawing capabilities and pnictogen donor atom identity on anion binding potency and selectivity. Furthermore, we also investigate the chloride anion recognition properties of novel tripodal PnB triaryl donors functionalised with additional electron‐deficient proto‐triazole HB donors, **2⋅Sb^R^** and **2⋅Bi^R^** (Figure 1b), as the first examples of mixed PnB‐HB anion host systems.

## Results and Discussion

### Synthesis of 1⋅Sb^R^ and 1⋅Bi^R^ receptor series

**Scheme 1:**
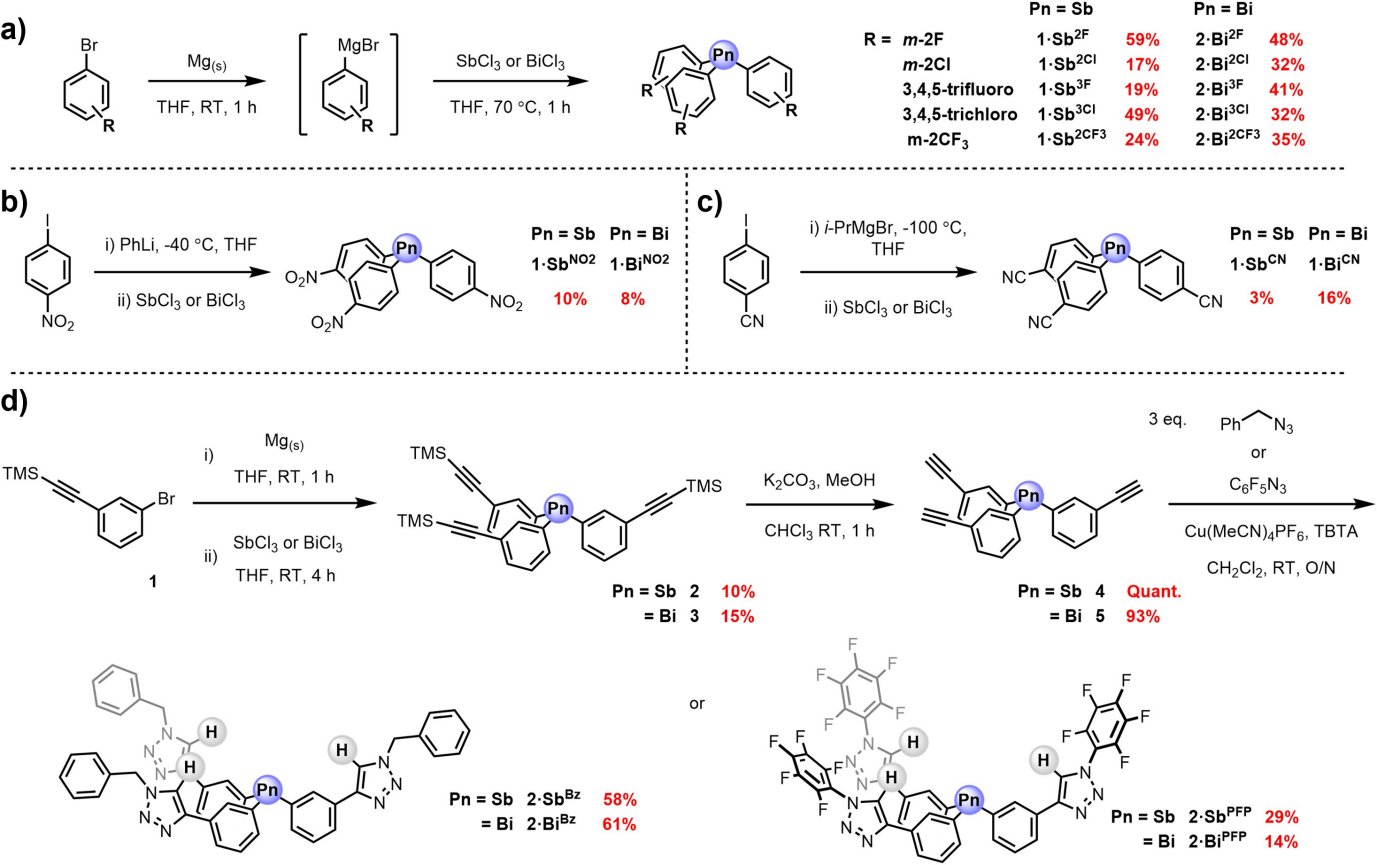
Synthesis of the first generation triaryl receptor series **1⋅Sb^R^** and **1⋅Bi^R^**; a) preparation and reaction of Grignard reagents. Metal‐halogen exchange methodology for triaryl pnictogen preparation using b) phenyl lithium or c) isopropylmagnesium bromide. d) Synthesis of the second generation tripodal PnB‐HB receptor systems **2⋅Sb^R^** and **2⋅Bi^R^**.

**Figure 2:**
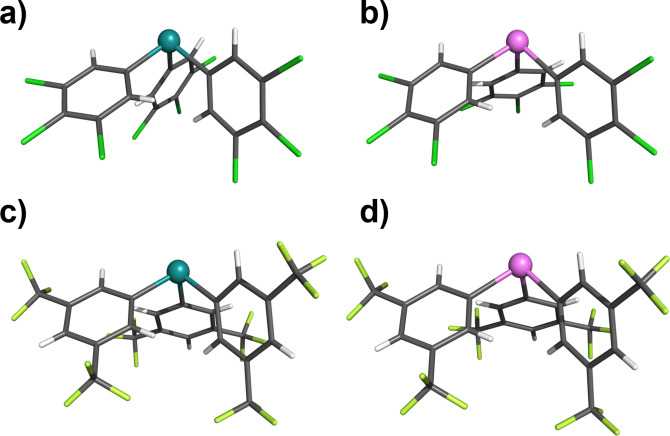
X‐ray crystallographic structures of a) **1⋅Sb^3Cl^** b) **1⋅Bi^3Cl^** c) **1⋅Sb^2CF3^** and d) **1⋅Bi^2CF3^**. Grey=carbon, white=hydrogen, green=fluorine, dark green=chlorine, teal=antimony, pink=bismuth.

In general, the first and second generation PnB receptor series were synthesised via treatment of the corresponding Grignard or organolithium reagent to a suspension of either antimony(III) or bismuth(III) trichloride, as summarised in Scheme 1 according to modified literature procedures.[^45^, ^46^] In the majority of cases this was achieved by in situ Grignard formation from the commercially available aryl bromide with freshly activated magnesium turnings in anhydrous THF. Subsequent addition of SbCl_3_ or BiCl_3_ afforded the target triaryl receptors, in variable yields, after purification by silica gel column chromatography. In the case of the cyano‐ and nitro‐appended triaryl pnictogens: **1⋅Sb^CN^**, **1⋅Bi^CN^**, **1⋅Sb^NO2^** and **1⋅Bi^NO2^** synthesis of the requisite organometallic precursor was achieved by a metal‐halogen exchange reaction between the appropriately functionalised aryl iodide and phenyllithium or isopropylmagnesium bromide. The consistently low yields for the cyano‐ and nitro‐triaryl pnictogens, for example 3 % for **1⋅Sb^CN^**, may be rationalised by undesired side reactions of organometallic reagents and the cyano or nitro groups.^[47]^ The 14 Sb(III) and Bi(III) receptors were characterised by ^1^H, ^13^C NMR, CHN elemental analysis and where possible single crystal X‐ray structural analysis, representative examples are shown in Figure 2.

### Synthesis of 2⋅Sb^R^ and 2⋅Bi^R^ receptor series

**Figure 3:**
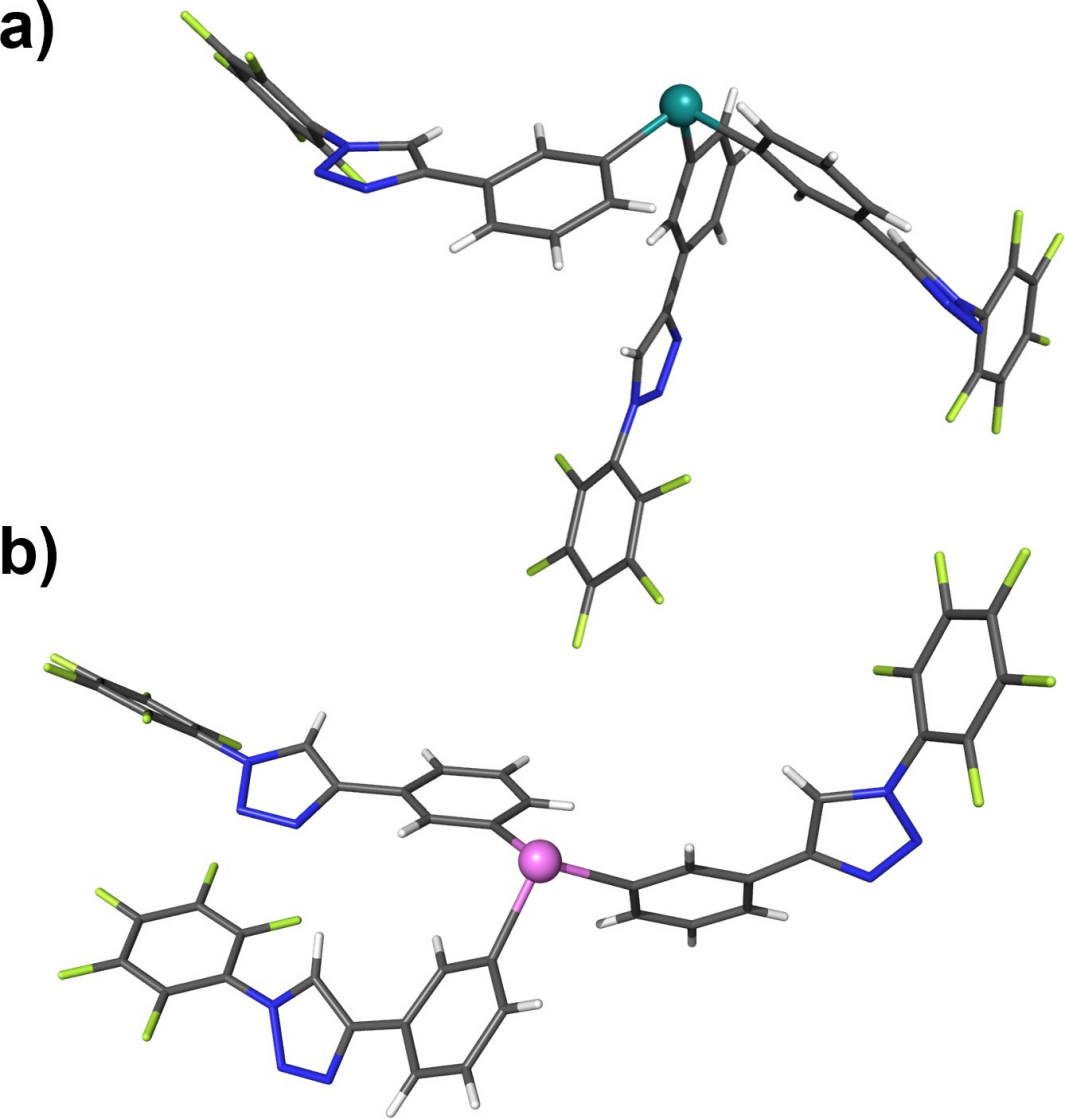
X‐ray crystallographic structures of a) **2⋅Sb^PFP^** b) **2⋅Bi^PFP^**. Grey=carbon, white=hydrogen, green=fluorine, teal=antimony, pink=bismuth.

The preparation of the tris‐proto‐triazole tripodal receptors is detailed in Scheme 1d. In a similar fashion to the first receptor series, a Grignard reagent prepared from a TMS‐protected alkyne aryl bromide **1**, which in turn was prepared from a chemoselective Sonogashira reaction between 1‐bromo‐3‐iodobenzene and TMS acetylene,^[48]^ was reacted with a suspension of either SbCl_3_ or BiCl_3_ to yield the tris‐TMS‐alkynes **2** and **3** in yields of 10 % and 15 % respectively. TMS deprotection was achieved through base promoted hydrolysis with K_2_CO_3_ in MeOH:CHCl_3_ mixtures to give the target proto‐alkynes **4** and **5** in excellent yield. The obtained tris‐alkynes were used in subsequent copper(I) catalysed azide–alkyne cycloaddition reactions with 3 equivalents of either benzyl or perfluorophenyl azide in the presence of catalytic TBTA and [Cu(MeCN)_4_]PF_6_ in anhydrous dichloromethane under an atmosphere of nitrogen. After stirring at room temperature for approximately 16 h, TLC analysis of the crude reaction mixtures indicated complete consumption of the alkyne starting materials and the formation of new species of considerably higher polarity. Subsequent NH_4_OH/EDTA_(aq)_ work‐up procedures and purification by column chromatography gave the four novel tris‐proto‐triazole containing Sb(III) and Bi(III) receptors, **2⋅Sb^Bz^**, **2⋅Bi^Bz^**, **2⋅Sb^PFP^** and **2⋅Bi^PFP^**, in yields in the range of 14–61 % and were characterised by ^1^H, ^13^C NMR spectroscopy, CHN elemental analysis and high resolution ESI‐MS. In addition, crystals of **2⋅Sb^PFP^** and **2⋅Bi^PFP^** suitable for X‐ray diffraction analysis were obtained from slow vapour diffusion of pentane into chloroform solutions of the tripodal receptors (Figure 3).

### Anion binding studies of 1⋅Sb^R^ and 1⋅Bi^R^

**Figure 4:**
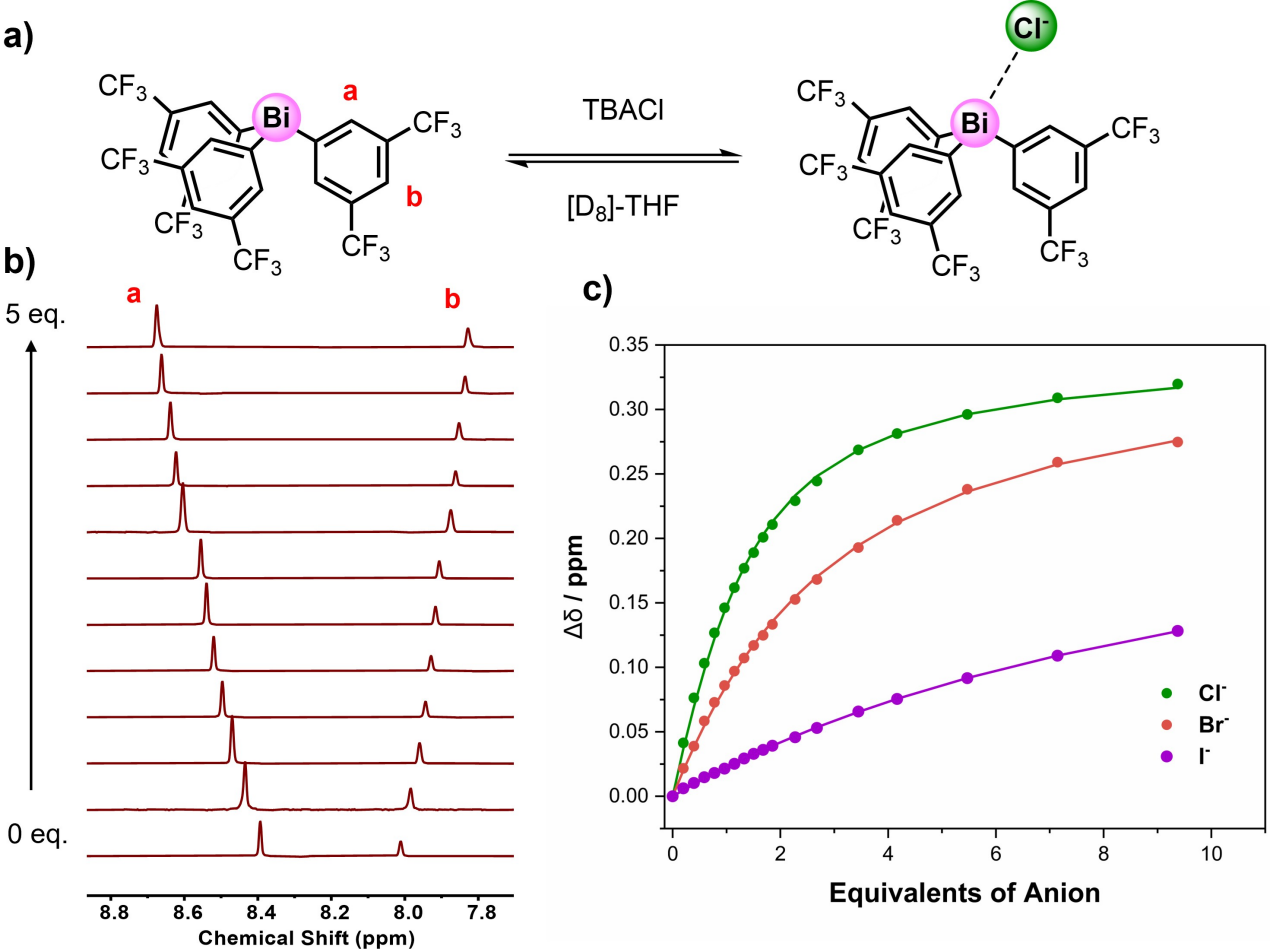
a) Chloride anion binding equilibrium for **1⋅Bi^2CF3^**. b) Truncated stacked ^1^H NMR titration spectra for **1⋅Bi^2CF3^** with increasing equivalents of TBACl ([D_8_]‐THF, 500 MHz, 298 K). c) Anion binding titration isotherm for **1⋅Bi^2CF3^** with chloride, bromide and iodide.

In order to assess the anion binding properties of the **1⋅Sb^R^** and **1⋅Bi^R^** receptor series, preliminary ^1^H NMR chloride anion titration experiments in [D_8_]‐THF were undertaken. In general, the addition of increasing equivalents of chloride, added as the tetrabutylammonium (TBA) salt, to solutions of the receptors induced significant perturbations of various proton resonances. Typically, the ortho‐substituted proton environment to the pnictogen centre exhibited the largest perturbations, consistent with an anion recognition event mediated by PnB⋅⋅⋅Cl^−^ interactions (Figure 4a and b). The generated isotherm binding titration data (Figure 4c), obtained by monitoring the chemical shift of the respective receptor's ortho proton signal was analysed using Bindfit,^[49]^ which determined 1 : 1 stoichiometric host:guest chloride association constants displayed in Table 1.^[50]^ Surveying the summarised *K_a_* (Cl^−^) values reveals several noteworthy trends. Firstly, with increasing electron withdrawing capability of the aryl substituents, reflected in the larger positive value of the aryl group's summed Hammett substituent constants (Σσ), there is a general increase in chloride affinity, consistent with an inductive activation of PnB donor atom electrophilicity, culminating in the largest association constants observed for the bis‐trifluoromethyl functionalised receptors **1⋅Sb^2CF3^** and **1⋅Bi^2CF3^** with *K_a_* (Cl^−^) values of 702 M^−1^ and 1,300 M^−1^ respectively. Furthermore, the bismuth triaryl receptors exhibit consistently enhanced chloride affinities relative to their antimony congeners, which is concordant with the accepted trend of increasing σ‐hole donor potency progressing down a given main group. To quantitatively evaluate the effect of changing the pnictogen atom donor identity in these receptor systems, chloride anion binding enhancement factors (*α*=*K*_a_^Bi^/*K*_a_^Sb^) were determined, facilitating comparison of the relative augmentation of association constant magnitudes. Interestingly, with the exception of the dichloro‐substituted aryl derivative,^[51]^ the binding enhancement factor is relatively consistent across the series, α≈2.

**Table 1:** Chloride anion association constants for **1⋅Sb^R^** and **1⋅Bi^R^** receptors.

	Chloride association constants [*K_a_*/M^‐1^]^[a]^		Binding enhancement factor α=[*K_a_* ^Bi^/ *K_a_* ^Sb^]	Σσ^[b]^
**R**	**1⋅Sb^R^**	**1⋅Bi^R^**		
CN	36 (2)	76 (1)	2.1±0.12	0.66
2F	61 (2)	152 (2)	2.5±0.09	0.674
2Cl	52 (1)	213 (1)	4.0±0.08	0.746
3F	253 (3)	580 (7)	2.3±0.04	0.736
NO_2_	94 (2)	227 (3)	2.4±0.06	0.778
3Cl	341 (6)	716 (8)	2.1±0.04	0.60
2CF_3_	702 (16)	1300 (43)	1.8±0.12	0.86

[a] Stoichiometric 1 : 1host–guest binding model calculated using Bindfit software in [D_8_]‐THF at 298 K. Errors (±) are in parentheses. [b] Summed Hammett substituent constants.

Analogous ^1^H NMR titration experiments with a range of anions including the remaining halides and a series of oxoanions were undertaken with the PnB receptors exhibiting the strongest chloride affinities, **1⋅Sb^2CF3^** and **1⋅Bi^2CF3^**. In all cases, similar downfield chemical shift perturbations were observed upon the addition of the anionic guests (Figure 4c). Bindfit analysis determined 1 : 1 host:guest stoichiometric association constants summarised in Table 2.

**Table 2:** Anion association constants for **1⋅Sb^2CF3^** and **1⋅Bi^2CF3^** receptors.

Anion	Anion association constants [*K_a_*/M^−1^]^[a]^		p*K_a_* of the anion's conjugate acid
	**1⋅Sb^2CF3^**	**1⋅Bi^2CF3^**	
Cl^−^	702 (16)	1300 (43)	−8.0
Br^−^	207 (3)	432 (8)	−9.0
I^−^	147 (4)	192 (2)	−10
AcO^−^	268 (15)	‐^[b]^	4.8
OCN^−^	263 (11)	344 (12)	3.7
NO_2_^−^	125 (4)	332 (6)	3.3
NO_3_^−^	79 (3)	124 (4)	−1.3

[a] Stoichiometric 1 : 1 host–guest binding model calculated using Bindfit software in [D_8_]‐THF at 298 K. Errors (±) are in parentheses. [b] Receptor decomposition.

As anticipated, **1⋅Bi^2CF3^** displays consistently larger anion *K_a_* values over the antimony analogue **1⋅Sb^2CF3^**. Halide anion binding affinities in both cases exhibit the trend of Cl^−^>Br^−^>I^−^ which mirrors decreasing halide charge density. Interestingly however, whilst HB based anion receptors typically display anion selectivity profiles dictated by anion basicity, wherein the more basic anion is bound more strongly, in contrast **1⋅Bi^2CF3^** and **1⋅Sb^2CF3^** both exhibit the largest *K_a_* value for chloride, despite OCN^−^ and NO_2_^−^ being more basic by at least 10 orders of magnitude. It is noteworthy to mention that this halide over oxoanion binding preference is a commonly observed selectivity trend for XB and ChB receptor systems, but not seen with traditional HB receptors. Despite successful acetate anion titration studies for **1⋅Sb^2CF3^**, analogous experiments conducted on the bismuth analogue resulted in immediate decomposition of the triaryl receptor, which presumably reflects relative strengths of Bi−C and Sb−C bonds.

### Anion binding studies of 2⋅Sb^R^ and 2⋅Bi^R^

**Figure 5:**
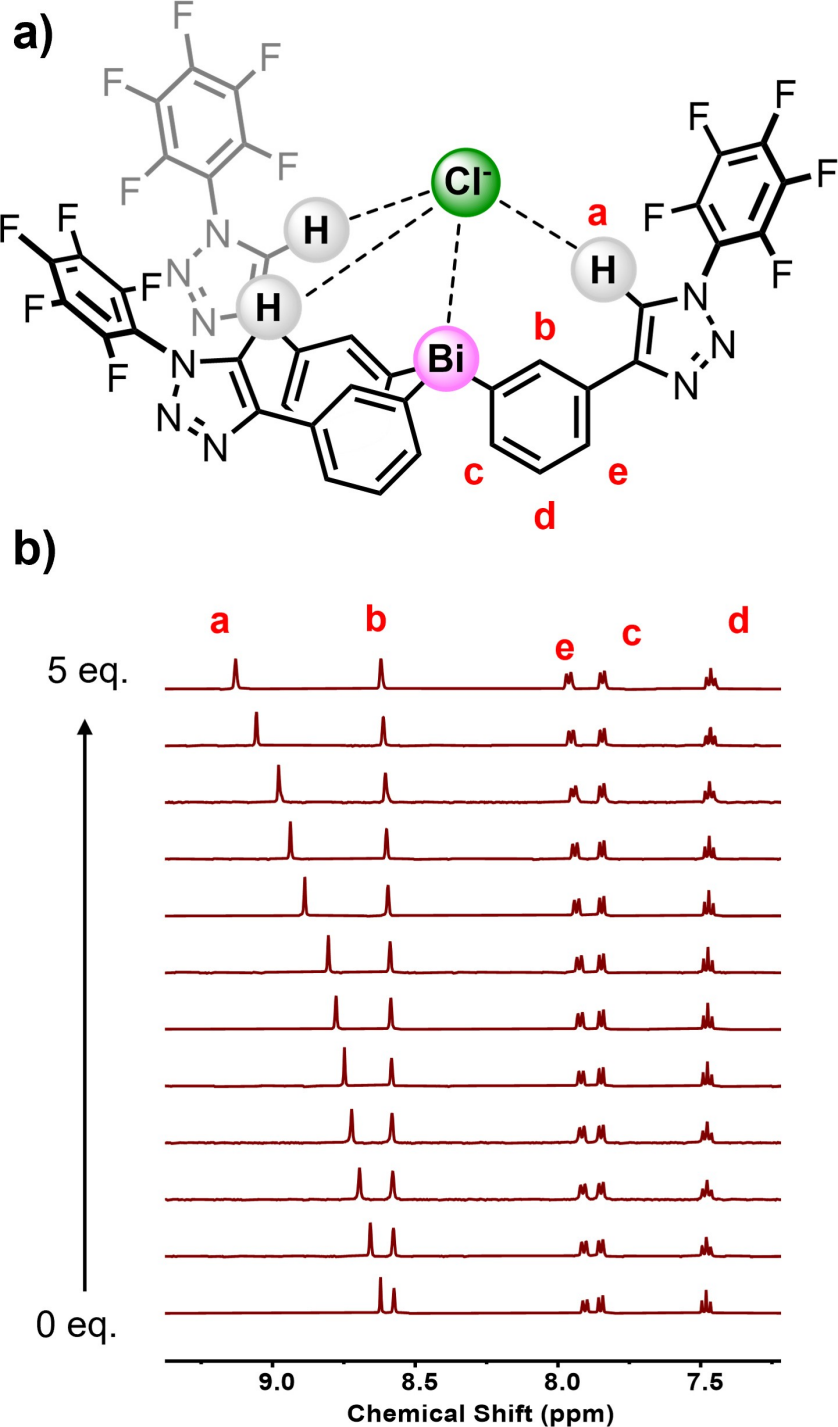
a) Postulated chloride anion binding equilibrium for **2⋅Bi^PFP^**. b) Truncated stacked ^1^H NMR titration spectra for **2⋅Bi^PFP^** with increasing equivalents of TBACl ([D_8_]‐THF, 500 MHz, 298 K).

Having demonstrated the modulation of the Pn(III) centre's anion binding capabilities by appending electron‐withdrawing aryl units, we sought to further exploit the triaryl pnictogen framework not only as a Lewis acidic PnB donor, but also as a functional tripodal scaffold which could be further elaborated with additional cooperative triazole HB donor anion binding sites as in **2⋅Sb^R^** and **2⋅Bi^R^** (Figure 5a). In order to probe this, benchmark TBACl ^1^H NMR titration experiments were conducted with **2⋅Sb^Bz^**, **2⋅Bi^Bz^**, **2⋅Sb^PFP^** and **2⋅Bi^PFP^** in [D_8_]‐THF. As with the first receptor series, the addition of increasing Cl^−^ equivalents is accompanied by chemical shift perturbations of proton signals corresponding to the triaryl framework. In addition, concomitant perturbations are also observed for the triazole C−H signals, which supports an anion binding mode with formation of PnB⋅⋅⋅Cl^−^ and HB⋅⋅⋅Cl^−^ interactions (Figure 5b).

Bindfit analysis of the titration data determined 1 : 1 host:guest stoichiometric association constants for the tripodal series which are summarised in Table 3. Similar to the first receptor series, inspection of the *K_a_* (Cl^−^) values for the tripodal receptor series reveals that the higher electrophilicity of Bi relative to Sb results in larger chloride affinities, by approximately a factor of two. Furthermore, the tripodal receptor series exhibits markedly enhanced chloride affinities relative to the corresponding parent triphenyl antimony (SbPh_3_) and bismuth (BiPh_3_) compounds, which exhibit no measurable chloride anion affinity.^[52]^ Importantly, this result serves to underline that the Cl^−^ binding exhibited by the tripods are indeed as a result of concerted and cooperative PnB and HB formation to the halide anionic guest.^[53]^ In terms of chloride anion binding affinity, the tripodal receptors appended with perfluoraryl groups, **2⋅Sb^PFP^** and **2⋅Bi^PFP^** outperform their benzyl functionalised analogues **2⋅Sb^Bz^** and **2⋅Bi^Bz^**. This is concordant with HB donor abilities typically correlating with C−H acidity, which is anticipated to be enhanced by the strongly electron deficient perfluoroaryl substituents.

**Table 3:** Chloride association constants for the **2⋅Sb^R^** and **2⋅Bi^R^** receptor series.

	Chloride association constants [*K_a_*/M^−1^]^[a]^
**2⋅Sb^Bz^**	25 (1)
**2⋅Sb^PFP^**	58 (1)
**SbPh_3_**	NB^[b]^
**2⋅Bi^Bz^**	63 (1)
**2⋅Bi^PFP^**	115 (1)
**BiPh_3_**	NB^[b]^

[a] Stoichiometric 1 : 1host–guest binding model calculated using Bindfit software in [D_8_]‐THF at 298 K. Errors (±) are in parentheses. [b] No binding.

## Conclusion

In summary, a library of acyclic Sb(III) and Bi(III) based triaryl PnB receptors have been prepared and extensive anion recognition studies undertaken. In the first receptor series **1⋅Sb^R^** and **1⋅Bi^R^**, it is demonstrated that the bismuth analogues consistently outperform their antimony counterparts in terms of anion affinity. Importantly the electrophilicity of the PnB donor is demonstrated to be highly tunable and sensitive to the electronic‐withdrawing nature of the appended aryl groups. Furthermore, the two most potent Sb‐ and Bi‐based PnB receptors: **1⋅Sb^2CF3^** and **1⋅Bi^2CF3^**, exhibit Cl^−^ selectivity relative to the heavier halides and, impressively, to a range of highly basic oxoanions, which is in stark contrast to anion selectivity profiles typically exhibited by traditional acyclic HB donor systems. We also report the synthesis and preliminary chloride anion binding investigations of a series of tripodal tris‐proto‐triazole triaryl pnictogen receptors as the first examples of a mixed PnB‐HB anion host system, wherein the Sb(III) or Bi(III) centre serves not only as a structural component providing a C_3V_ scaffold for C−H HB donor arrays, but also as a functional Lewis acidic PnB donor. In comparison to the corresponding parent triphenyl antimony (SbPh_3_) and bismuth (BiPh_3_) compounds, which are unable to bind Cl^−^ in deuterated THF solvent media, the mixed PnB‐triazole HB host systems display notable halide affinity. Importantly, all of these findings not only demonstrate the electronic substituent tunability of PnB donor anion binding potency, but also the potential for PnB integration into structural multifaceted anion host framework design, the subject of which is continuing in our laboratories.

## Experimental Section

General information as well as further details about compound synthesis, characterisation and X‐ray diffraction data are detailed in the Supporting Information.

Deposition Number(s) 2176084 (for **1⋅Sb** ^**2Cl**^), 2176087 (for **1⋅Sb** ^**3Cl**^), 2176083 (for **1⋅Sb** ^**2CF3**^), 2176085 (for **1⋅Bi** ^**2Cl**^), 2176088 (for **1⋅Bi** ^**3Cl**^), 2176082 (for **1⋅Bi** ^**2CF3**^), 2176089 (for **2⋅Sb** ^**PFP**^) and 2176086 (for **2⋅Bi** ^**PFP**^) contain(s) the supplementary crystallographic data for this paper. These data are provided free of charge by the joint Cambridge Crystallographic Data Centre and Fachinformationszentrum Karlsruhe Access Structures service.

## Conflict of interest

The authors declare no conflict of interest.

1

## Supporting information

As a service to our authors and readers, this journal provides supporting information supplied by the authors. Such materials are peer reviewed and may be re‐organized for online delivery, but are not copy‐edited or typeset. Technical support issues arising from supporting information (other than missing files) should be addressed to the authors.

Supporting Information

## Data Availability

The data that support the findings of this study are available in the supplementary material of this article.
