# Supplementary material for: Anion Recognition with Antimony(III) and Bismuth(III) Triaryl‐Based Pnictogen Bonding Receptors
Source: Chemistry. 2022 Oct 11;28(67):e202201838. doi: 10.1002/chem.202201838 (PMC10092038; doi:10.1002/chem.202201838)
Supplement: Supplementary file 1 — Supporting Information [file CHEM-28-0-s001.pdf]

Chemistry–A European Journal

Supporting Information

Anion Recognition with Antimony(III) and Bismuth(III) Triaryl-Based Pnictogen Bonding Receptors

Heike Kuhn, Andrew Docker, and Paul D. Beer*

1 Materials and Methods 2
2 General Synthetic Procedures 2
2.1 Triaryl Pnictogens 2
2.2 Pnictogen Tripods 2
3 Characterisation 4
3.1 Triaryl Pnictogens 4
3.2 Pnictogen Tripods 13
4 CHN Elemental Analysis 30
5 Titration Data 32
5.1 General Procedure 32
5.2 Bismuth Triaryl Compounds 33
5.3 Antimony Triaryl Compounds 39
5.4 Pnictogen Tripods 45
6 Single Crystal X-Ray Diffraction 50
7 References 54

1 Materials and Methods

Solvents and reagents were purchased from commercial supplier and used as received. Dry solvents were obtained by purging with nitrogen and passing through a MBraun MPSP-800 column. H_2O was de-ionised and micro filtered using a Milli-Q® Millipore machine. Experiments were conducted at room temperature unless otherwise stated. Merck silica gel 60 was used for flash column chromatography. TBA salts were stored in vacuum desiccators prior to use. NMR spectra were either recorded on a Bruker Avance III HD Nanobay NMR equipped with a 9.4T magnet or a Bruker NEO 600 with broadband helium cryoprobe. ^{1}H-NMR titrations were recorded on a Bruker Avance III NMR equipped with a 11.75T magnet. Chemical shifts are quoted in parts per million relative to the residual solvent peak.

2 General Synthetic Procedures

2.1 Triaryl Pnictogens

Compounds **1·Bi^{2F}**, **1·Bi^{3F}**, **1·Bi^{2Cl}**, **1·Bi^{3Cl}** and **1·Bi^{2CF3}** as well as **1·Sb^{2F}, 1·Sb^{3F}, 1·Sb^{2Cl}, 1·Sb^{3Cl}** and **1·Sb^{2CF3}** were prepared according to general procedure 1 (Scheme S1).

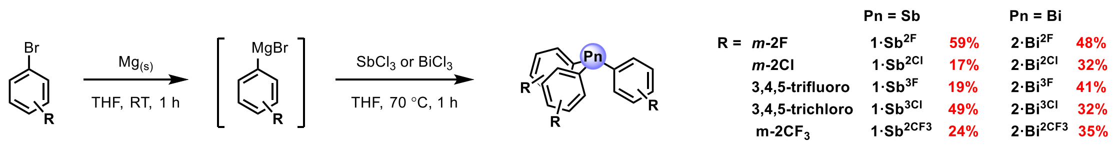

Scheme S1. Synthesis of PnB receptors via Grignard formation

Compounds **1·Bi^{CN}, 1·Bi^{NO2}, 1·Sb^{CN}** and **1·Sb^{NO2}** were prepared according to literature procedures (Scheme S2).[1, 2]

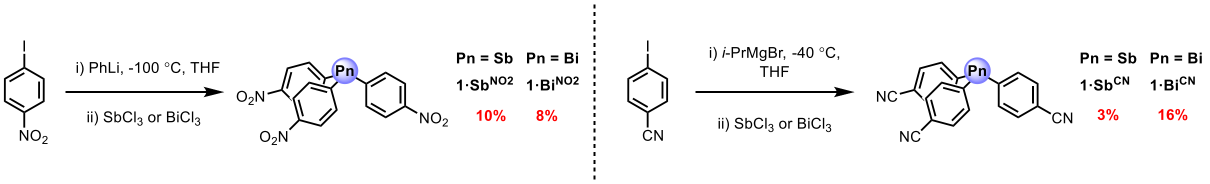

Scheme S2. Synthesis of receptors **1·PnB^{NO2}** *(left) and* **1·PnB^{CN}** *(right) according to literature procedures*[1, 2]

General Procedure 1 (adapted from[3], Scheme S1)
Magnesium turnings (3 eq.) with I_2 (cat.) were briefly heated with a heat gun. Once cooled, dry THF was added. Aryl bromide (3 eq.) was added dropwise either neat or as a concentrated THF solution over half an hour. The mixture was left to stir for 1 h. A solution of $SbCl_3$ or $BiCl_3$ (1 eq.) in THF was added slowly at 0 °C. The mixture was left to stir at room temperature for 1 h and heated at 70 °C for 1 h. After cooling to room temperature, the mixture was diluted with brine and extracted with EtOAc. The combined organic phases were dried over $MgSO_4$, filtered, and concentrated under reduced pressure. The crude products were purified using silica gel flash column chromatography.

2.2 Pnictogen Tripods

Compounds **2·Sb^{Bz}**, **2·Sb^{PFP}, 2·Bi^{Bz}** and **2·Bi^{PFP}** were prepared using general procedures 2 – 4 (Scheme S3). 2-(3-bromophenyl)ethynyltrimethylsilane[4] and perfluorophenyl azide[5] were prepared according to literature procedure and spectroscopic data were consistent with those

reported. Benzyl azide was prepared according to a modified literature procedure.[6] Benzyl bromide was dissolved (15 g, 87.7 mmol, 1 eq.) was dissolved in DMSO and sodium azide (6.8 g, 65.02 mmol, 1.2 eq.) was added. The mixture was stirred at room temperature overnight before diluting it with water and extracting the product with diethyl ether before drying over $MgSO_4$ and concentrating to afford the clear liquid in quantitative yield. Spectroscopic data were consistent with those reported.[6]

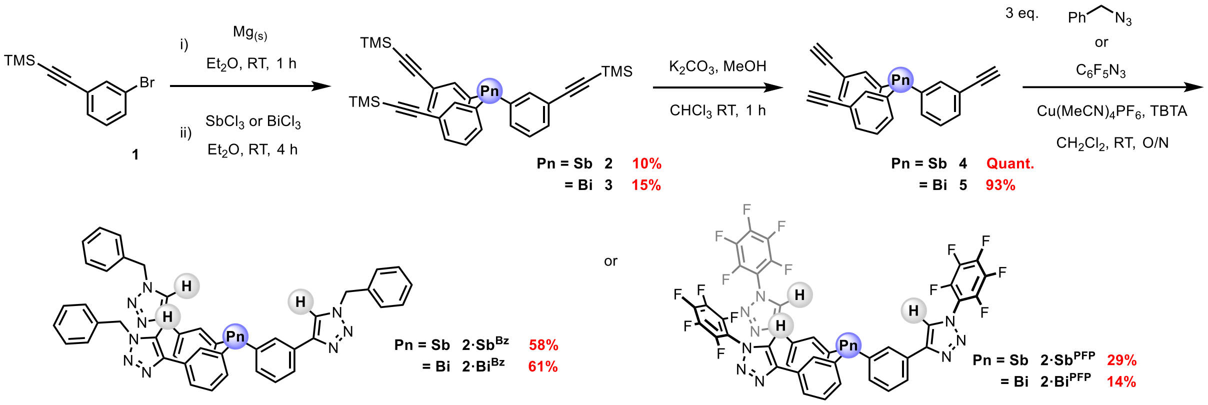

Scheme S3.Formation of pnictogen tripods according to general procedures 2-4

General Procedure 2 (adapted from[7], see Scheme S3)
Dry, degassed THF (5.5 mL) was added to a flask containing magnesium turnings (0.27 g, 11.06 mmol, 1.4 eq.) and iodine (cat.) and the resultant mixture was left to stir for 10 minutes. 2-(3-bromophenyl)ethynyltrimethylsilane (2 g, 7.90 mmol, 1 eq.) was added dropwise over 3 h using a syringe pump. Once the Grignard was fully activated, a suspension of the pnictogen(III) trichloride (2.63 mmol, 1/3 eq.) in THF (5 mL) was added slowly. The resulting reaction mixture was left to stir at room temperature overnight, before being quenched with MeOH (3 mL). The solvents were evaporated under reduced pressure. The crude was dissolved in EtOAc and washed with brine. The organic layer was dried over $MgSO_4$, filtered and concentrated. The product was purified using silica gel flash column chromatography.

General Procedure 3 (see Scheme S3)
TMS protected triaryl pnictogen (0.16 mmol, 1 eq.) was dissolved in a 1:1 mixture of MeOH and $CHCl_3$ with a few drops of H_2O. K_2CO_3 (0.32 g, 2.34 mmol, 15 eq.) was added and the mixture stirred at room temperature for 5 h until TLC (5% DCM in Hexane) showed full conversion of the starting material. The solvent was removed under reduced pressure and the crude mixture dissolved in $CHCl_3$ and washed with brine. The organic layer was dried over $MgSO_4$, filtered and concentrated.

General Procedure 4 (see Scheme S3)
$[Cu(MeCN)_4]PF_6$ (32 mg, 0.087 mmol, 0.3 eq.) and TBTA (46 mg, 0.087 mmol, 0.3 eq.) were complexed in dry, degassed DCM (3 mL) for 30 minutes. The respective pnictogen(III) triaryl alkyne was added (0.29 mmol, 1 eq.) in dry, degassed DCM (2 mL) before the addition of either perfluorophenyl azide (0.20 g, 0.95 mmol, 3.3 eq.) or benzyl azide (0.13 g, 0.95 mmol, 3.3 eq.). The resulting mixture was stirred at room temperature overnight. The crude was diluted with DCM and washed with an EDTA/$NH_4OH_{(aq)}$ solution followed by brine before drying the organic layer over $MgSO_4$, filtering and concentrating under reduced pressure. Products were purified using silica gel flash column chromatography.

3 Characterisation

3.1 Triaryl Pnictogens

Spectroscopic data of **1·Bi**$^{\mathbf{CN}}$ [1], **1·Bi**$^{\mathbf{NO2}}$ [1], **1·Bi**$^{\mathbf{2F}}$ [3], **1·Bi**$^{\mathbf{3F}}$ [8], **1·Sb**$^{\mathbf{CN}}$ [2], **1·Sb**$^{\mathbf{NO2}}$ [2], **1·Sb**$^{\mathbf{3F}}$ [8], **1·Sb**$^{\mathbf{2Cl}}$ [9], **1·Sb**$^{\mathbf{2CF3}}$ [10] were consistent with those reported in the literature.

1·Bi$^{\mathbf{2Cl}}$

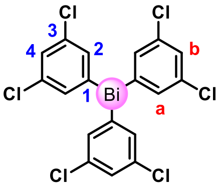

The compound was synthesised according to general procedure 1 and purified using silica gel flash column chromatography (5% EtOAc/Hexane) to afford a white solid in 32% yield. **^{1}H NMR** (400 MHz, Acetone-d_6) δ 7.84 (d, J = 1.9 Hz, 6H, H_a), 7.45 (t, J = 1.9 Hz, 3H, H_b). **^{13}C NMR-{^{1}H}** (101 MHz, Acetone-d_6) δ 165.98 (s, C_1), 138.90 (s, C_3), 136.45 (s, C_2), 128.95 (s, C_4). The compound was further characterised by XRD.

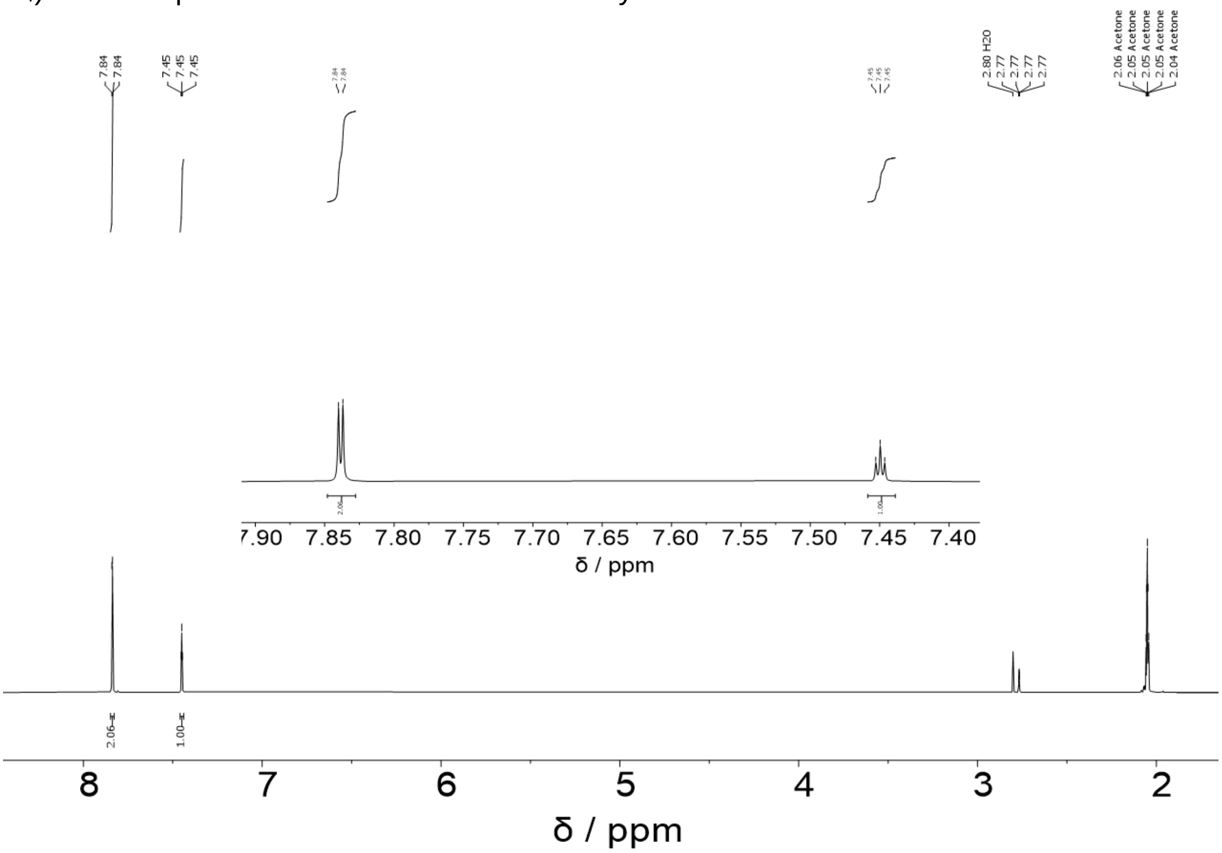

*Figure S1. ^{1}H NMR spectrum of **1·Bi**$^{\mathbf{2Cl}}$ (400 MHz, Acetone-d_6, 298K)*

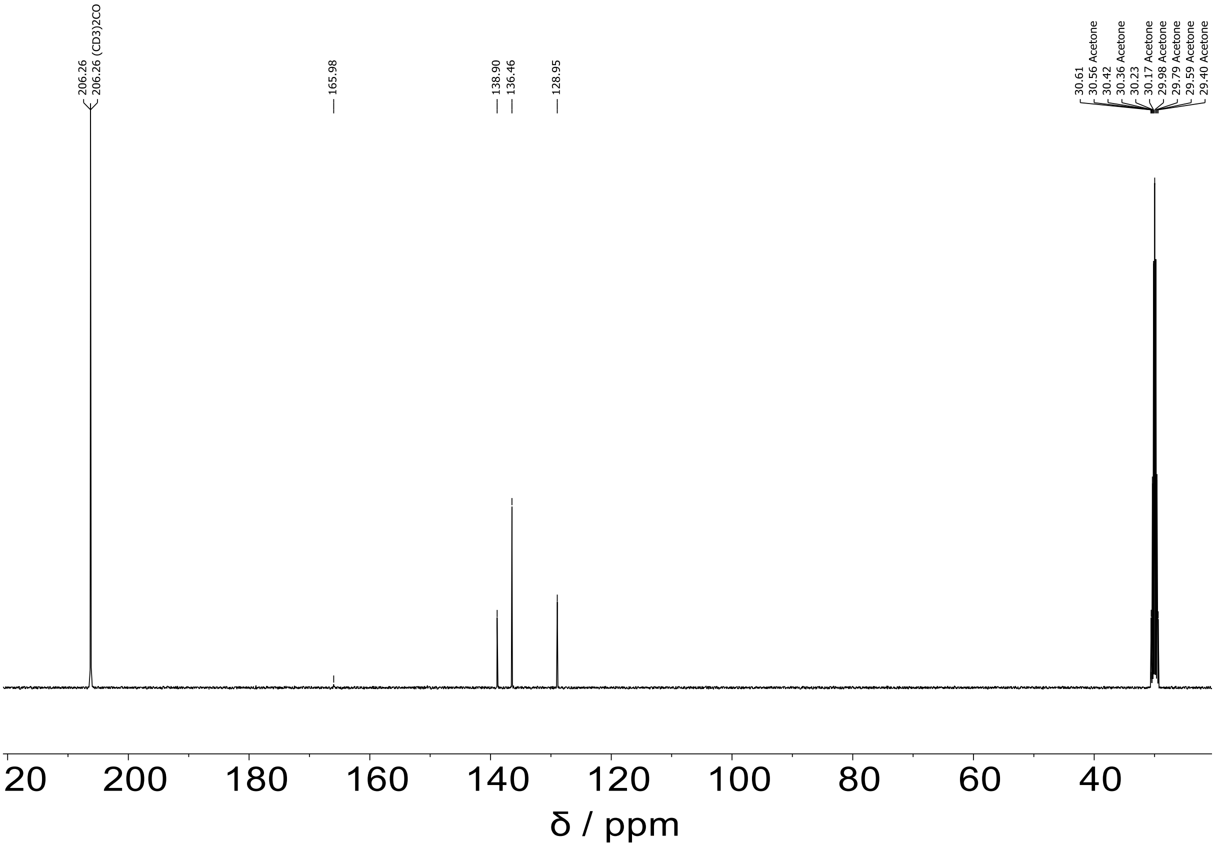

*Figure S2. ^{13}C NMR spectrum of **1·Bi^{2Cl}** (101 MHz, Acetone-d$_6$, 298K)*

1·Bi^{3Cl}

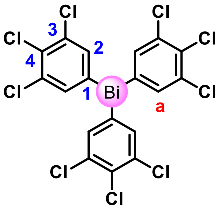

The compound was synthesised according to general procedure 1 and purified using silica gel flash column chromatography (5% EtOAc/Hexane) to afford a white solid in 32% yield. **^{1}H NMR** (600 MHz, Acetone-d$_6$) δ 8.05 (s, 6H, H$_a$). **^{13}C NMR-{^{1}H}** (151 MHz, Acetone-d$_6$) δ 137.61 (s, C$_3$), 136.79 (s, C$_2$), 130.34 (s, C$_4$). Limited solubility of the compound combined with relaxation effects resulted in the inability to observe the peak corresponding to carbon adjacent to the central Bi atom. The compound was further characterised by XRD.

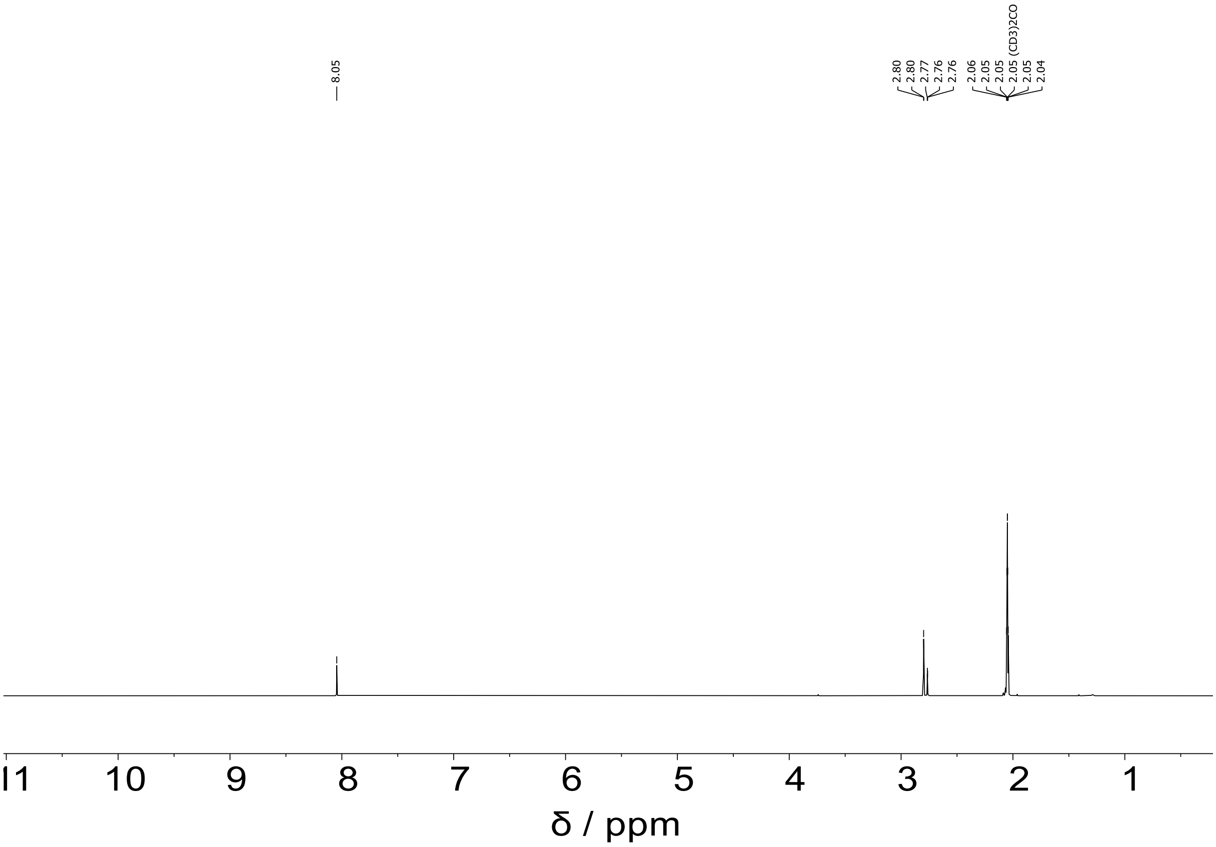

*Figure S3. ^{1}H NMR spectrum of **1·Bi^{3Cl}** (600 MHz, Acetone-d$_6$, 298K)*

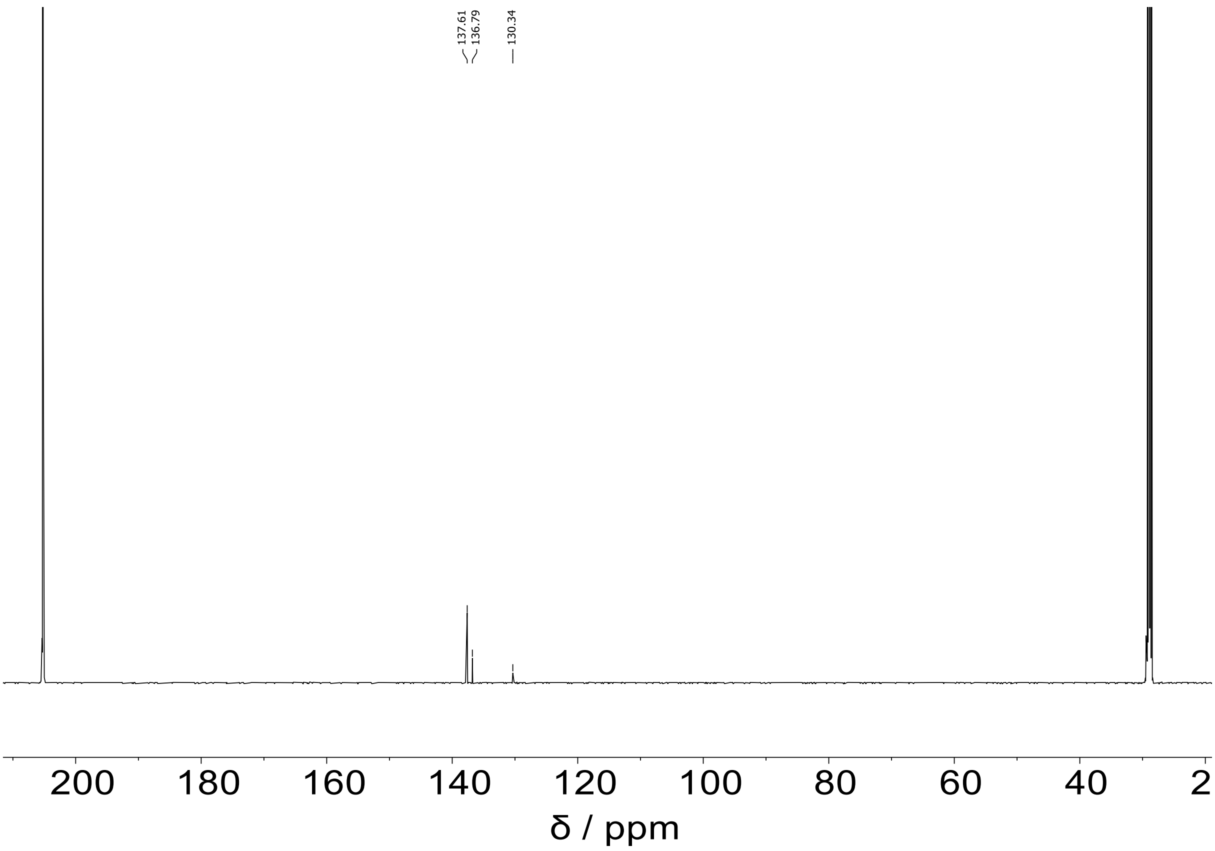

*Figure S4. ^{13}C NMR spectrum of **1·Bi^{3Cl}** (151 MHz, Acetone-d$_6$, 298K)*

1·Bi[2CF3]

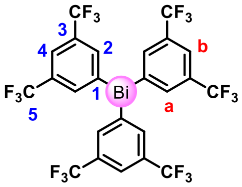

The compound was synthesised according to general procedure 1 and purified using silica gel flash column chromatography (5% EtOAc/Hexane) to afford a yellow oil which was recrystalised from hexane to afford the product as a pale-yellow solid in 35% yield. **^{1}H NMR** (600 MHz, Acetone-d_6) δ 8.55 (s, 6H, H*a*), 8.02 (s, 3H, H*b*). **^{13}C NMR-{^{1}H}** (151 MHz, Acetone-d_6) δ 167.25 (s, C*1*), 139.20 (q, *J* = 3.8 Hz, C*2*), 133.57 (q, *J* = 32.6 Hz, C*3*), 124.98 (q, *J* = 272.5 Hz, C*5*), 122.79 (hept, *J* = 4.1 Hz, C*4*). **^{19}F NMR** (377 MHz, Acetone-d_6) δ -63.46. The compound was further characterised by XRD.

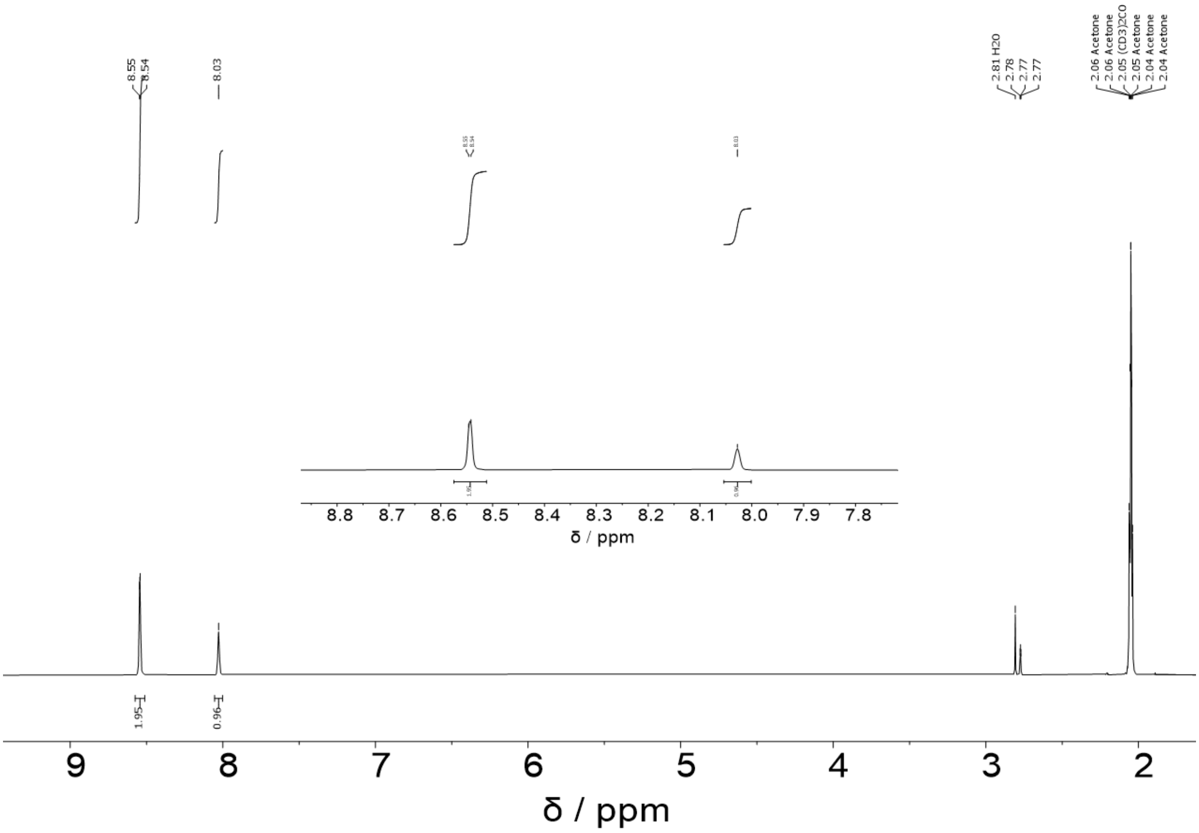

*Figure S5. ^{1}H-NMR spectrum of **1·Bi[2CF3]** (600 MHz, Acetone-d_6, 298K)*

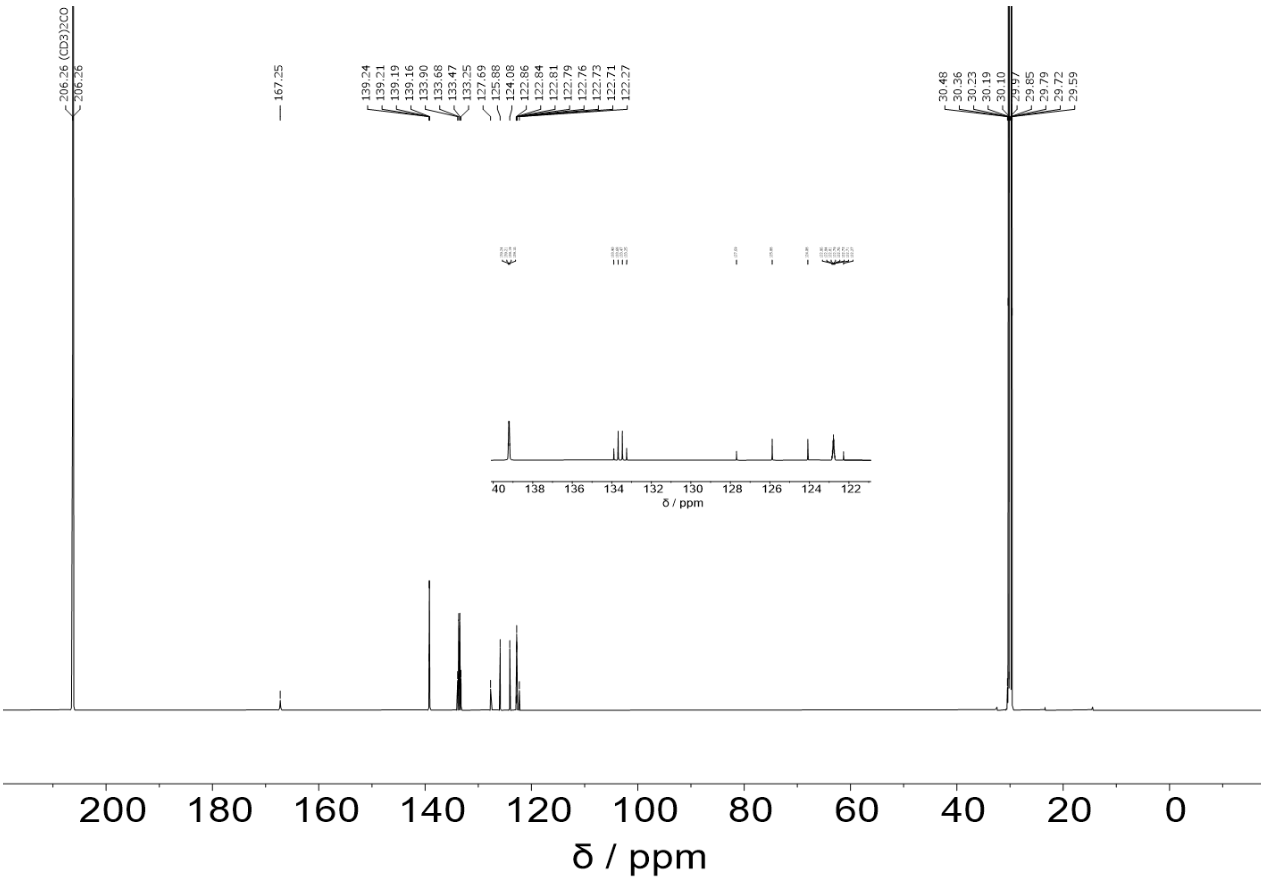

Figure S6. *^{13}C-NMR spectrum of* ***1·Bi^{2CF3}*** *(151 MHz, Acetone-d$_6$, 298K)*

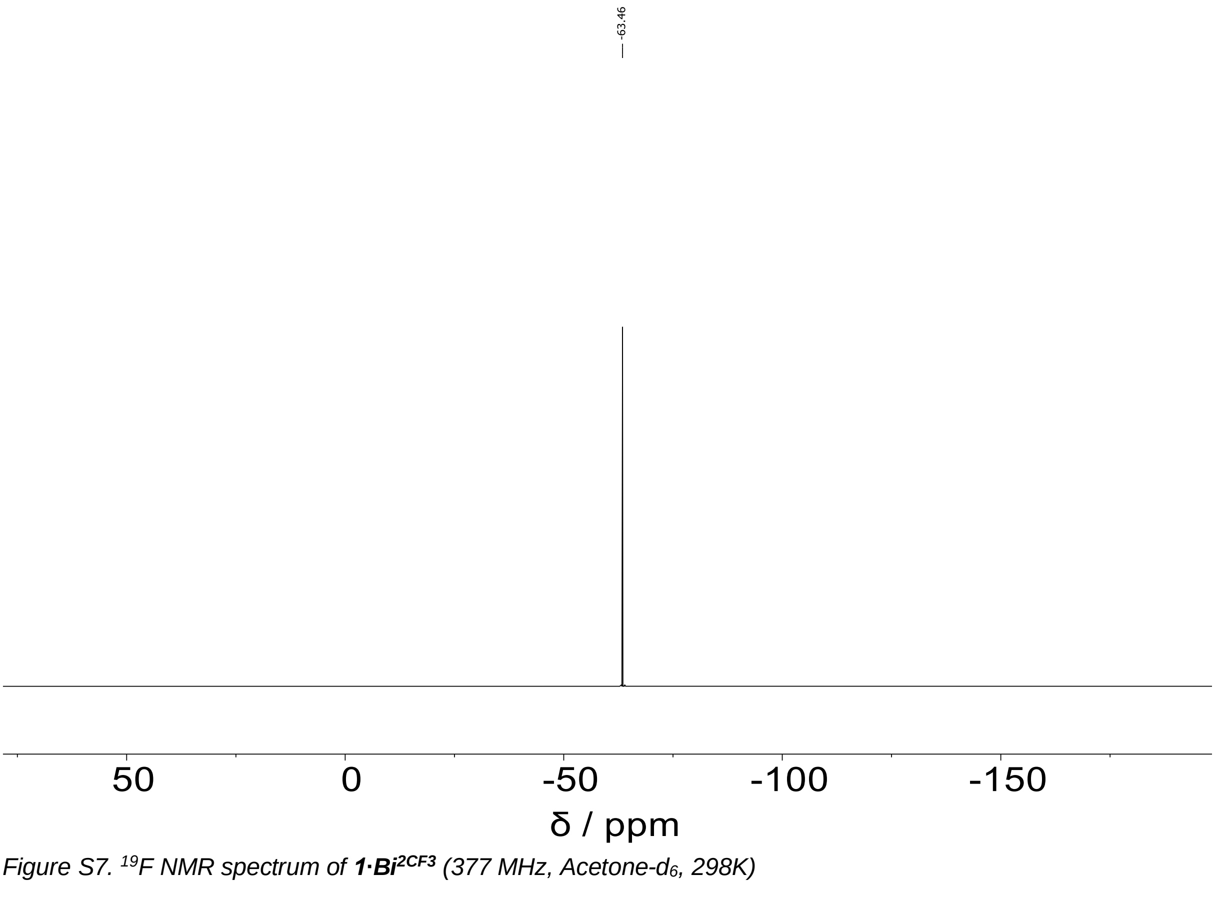

Figure S7. *^{19}F NMR spectrum of* ***1·Bi^{2CF3}*** *(377 MHz, Acetone-d$_6$, 298K)*

1·Sb^{2F}

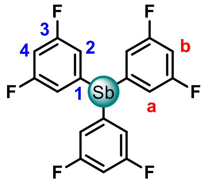

The compound was synthesised according to general procedure 1 and purified using silica gel flash column chromatography (9:1 Hexane:DCM) to afford a white solid in 59% yield. **^{1}H NMR** (400 MHz, Acetone-d$_6$) δ 7.20 – 7.11 (m, 6H, H$_a$), 7.07 (tt, J = 9.3, 2.3 Hz, 3H, H$_b$). **^{13}C NMR-{^{1}H}** (101 MHz, Acetone-d$_6$) δ 164.35 (dd, J = 253.3, 10.4 Hz, C$_3$), 143.57 (t, J = 4.2 Hz, C$_1$), 119.47 (dd, J = 14.7, 5.7 Hz, C$_2$), 105.71 (t, J = 25.5 Hz, C$_4$). **^{19}F NMR** (377 MHz, Acetone-d$_6$) δ -110.34 – -110.39 (m).

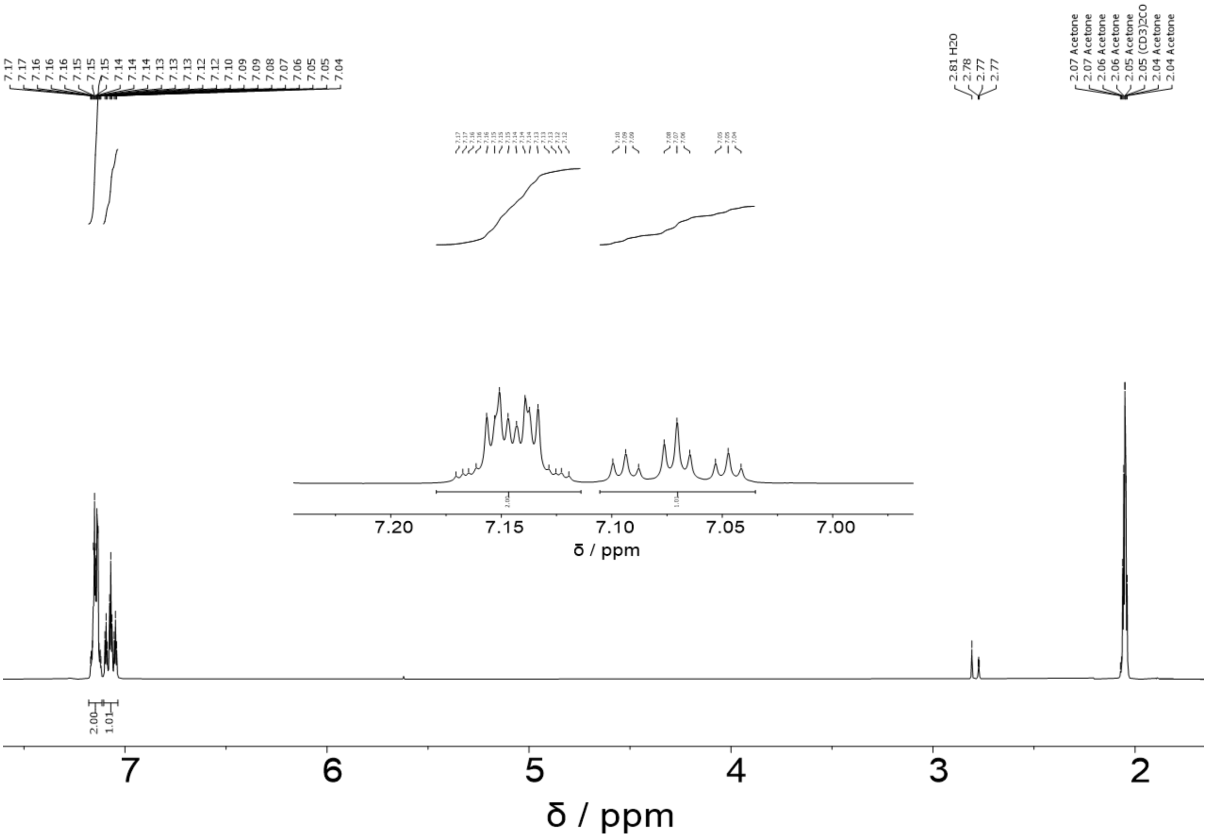

*Figure S8. ^{1}H NMR spectrum of **1·Sb^{2F}** (400 MHz, Acetone-d$_6$, 298 K)*

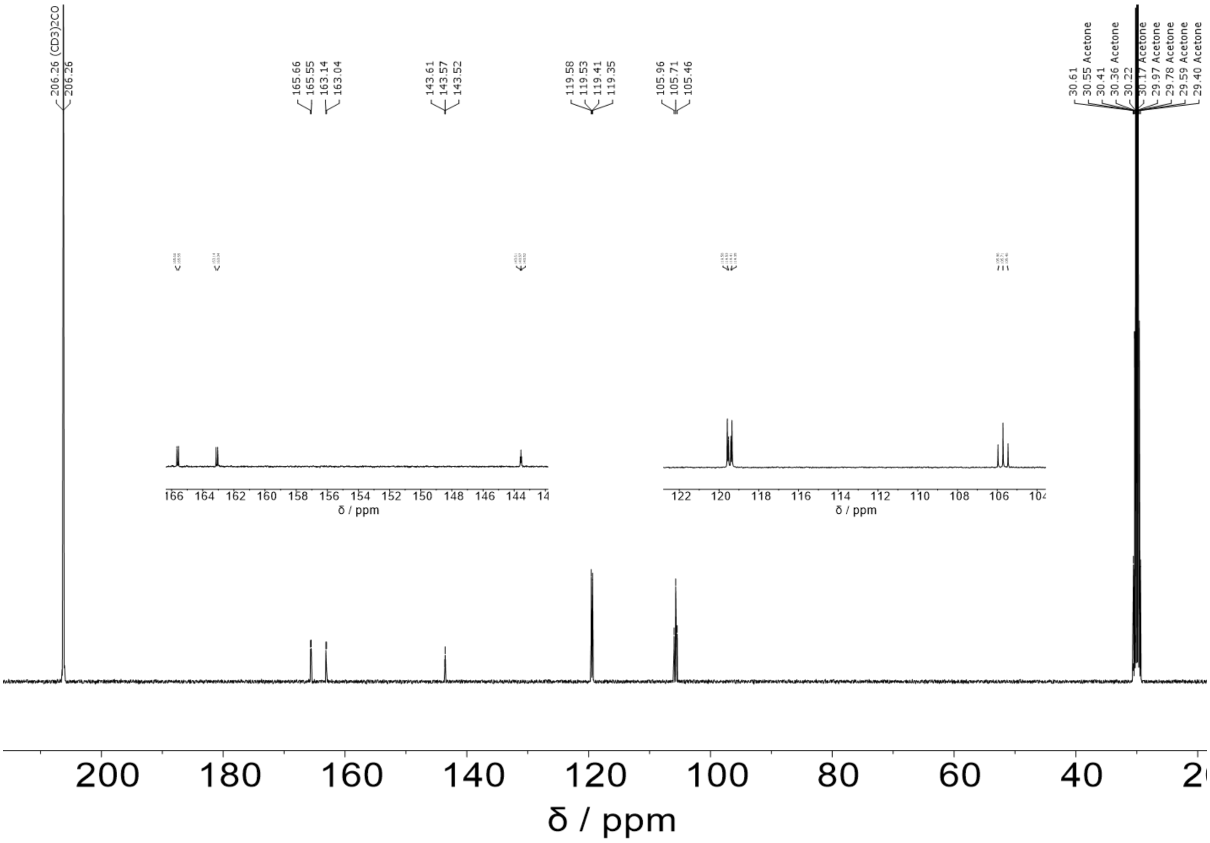

*Figure S9. ^{13}C NMR spectrum of **1·Sb^{2F}** (101 MHz, Acetone-d$_6$, 298K)*

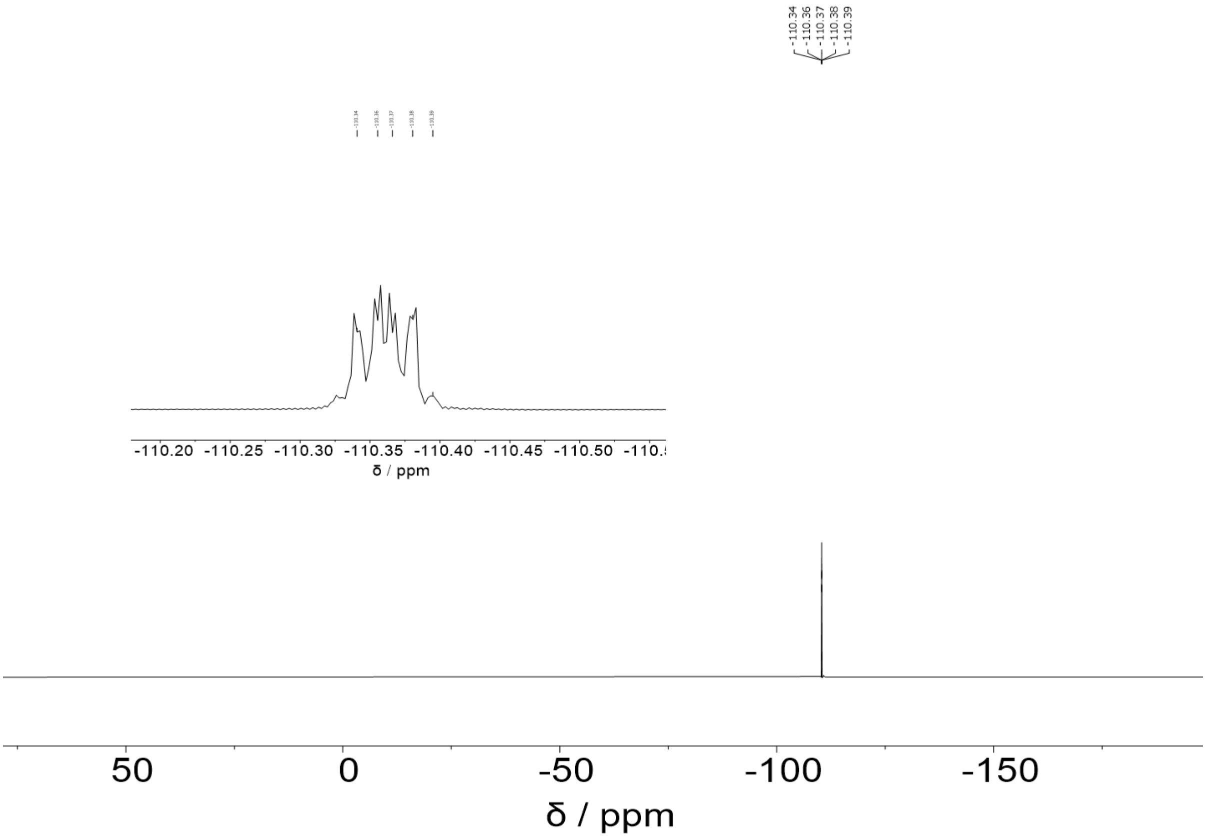

*Figure S10. ^{19}F NMR spectrum of **1·Sb^{2F}** (377 MHz, Acetone-d$_6$, 298K)*

1·Sb^3Cl^

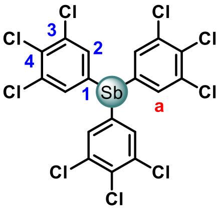

The compound was synthesised according to general procedure 1 and purified using silica gel flash column chromatography (9:1 Hexane:DCM) to afford a white solid in 49% yield. **^{1}H NMR** (600 MHz, Acetone-d_6) δ 7.72 (s, 6H, H*a*). **^{13}C NMR-{^{1}H}** (151 MHz, Acetone-d_6) δ 140.97 (s, C*1*), 137.04 (s, C*3*), 135.51 (s, C*2*), 132.74 (s, C*4*). The compound was further characterised by XRD.

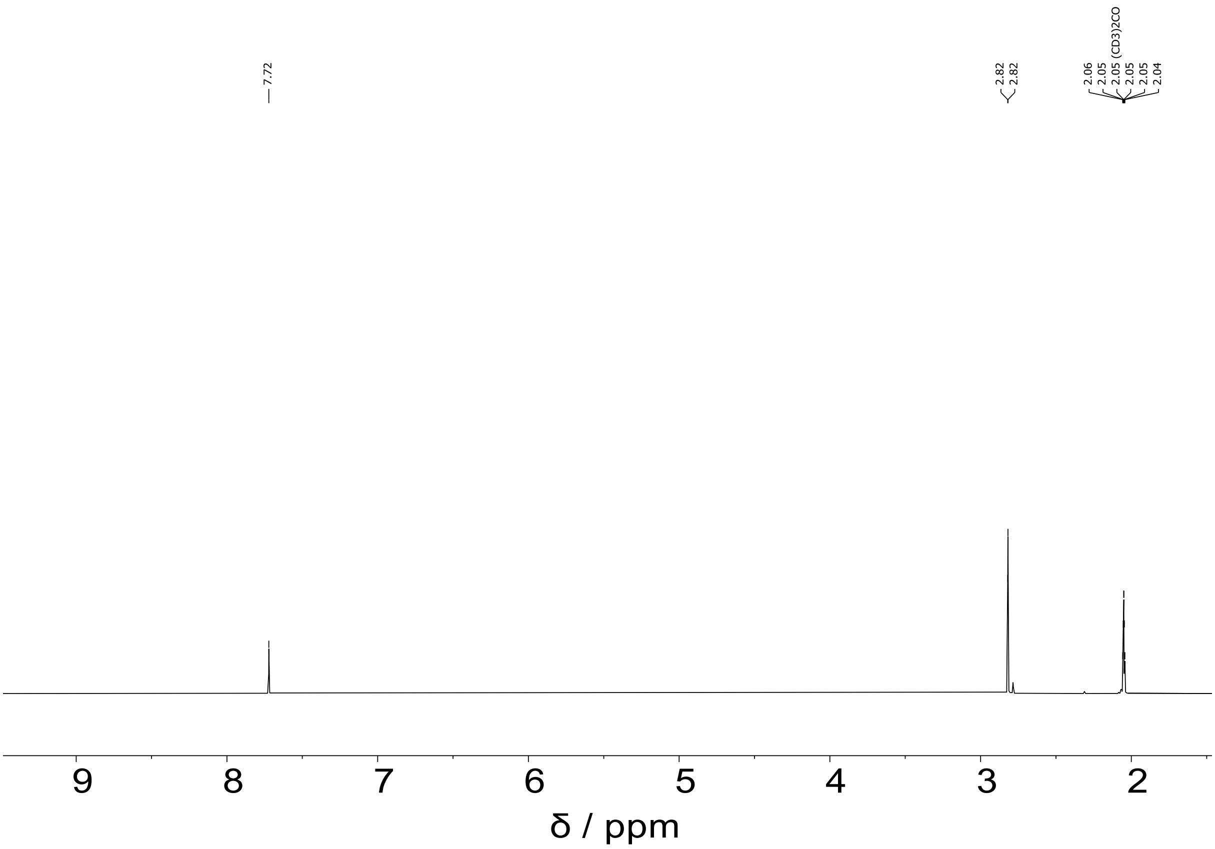

*Figure S11. ^{1}H-NMR spectrum of **1·Sb^{3Cl}** (600 MHz, Acetone-d_6, 298K)*

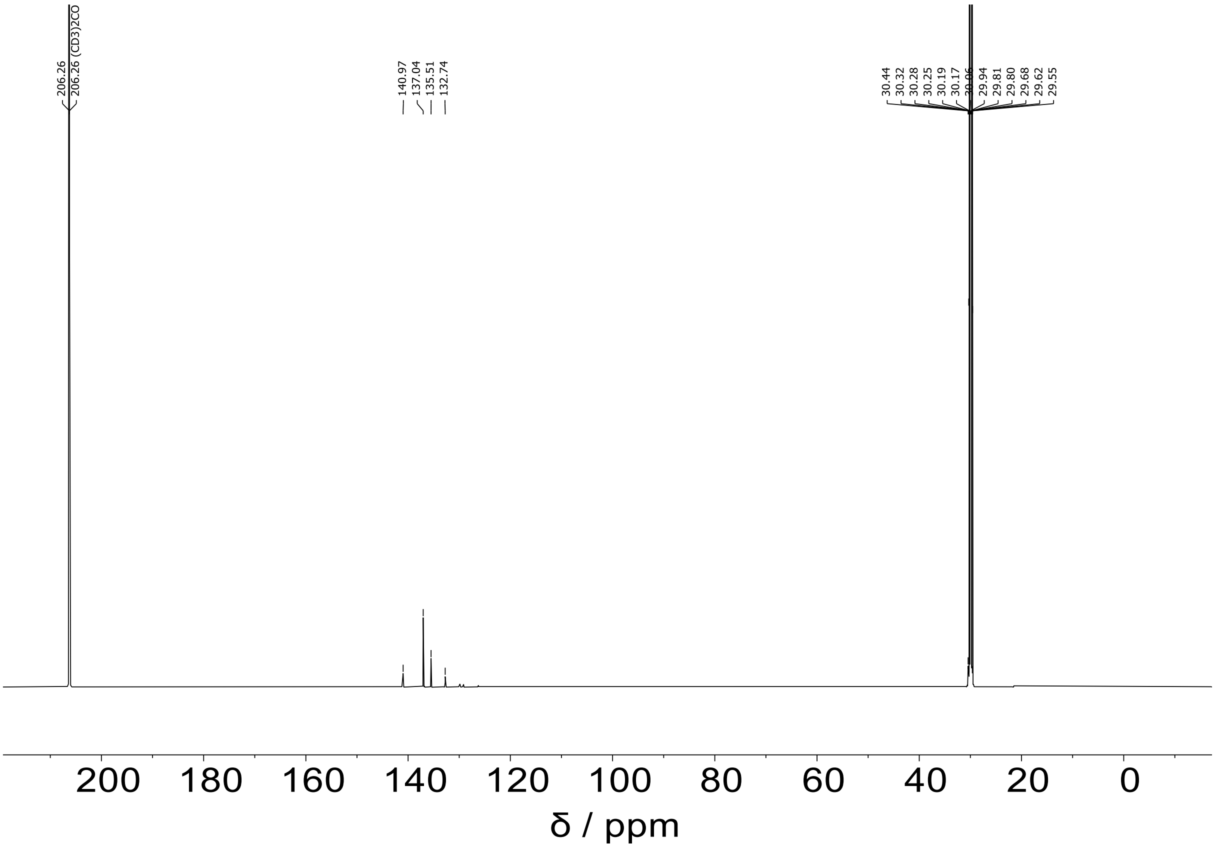

*Figure S12. ^{13}C NMR of **1·Sb^{3Cl}** (151 MHz, Acetone-d$_6$, 298K)*

3.2 Pnictogen Tripods

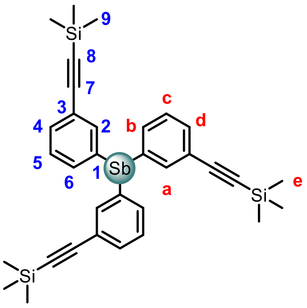

Compound **2** was prepared according to general procedure 2 and purified using silica gel flash column chromatography (5% DCM in Hexane). If required, the product can further be purified by dissolving it in minimal amounts of EtOAc and then adding MeOH, causing the pure product to crush out, which can then be isolated by vacuum filtration as a white solid (0.51 g, 0.79 mmol, 10%). **^{1}H NMR** (600 MHz, Acetone-d$_6$) δ 7.57 (q, J = 1.1 Hz, 1H, H$_{a}$), 7.51 – 7.35 (m, 3H, H$_{b\text{-}d}$), 0.20 (s, 9H, H$_{e}$). **^{13}C NMR-{^{1}H}** (151 MHz, Acetone-d$_6$) δ 140.11 (s, C$_{2}$), 139.59 (s, C$_{1}$), 137.26 (s, C$_{6}$), 133.34 (s, C$_{4}$), 130.11 (s, C$_{5}$), 125.07 (s, C$_{3}$), 105.86 (s, C$_{7}$), 95.65 (s, C$_{8}$), 0.13 (s, C$_{9}$).

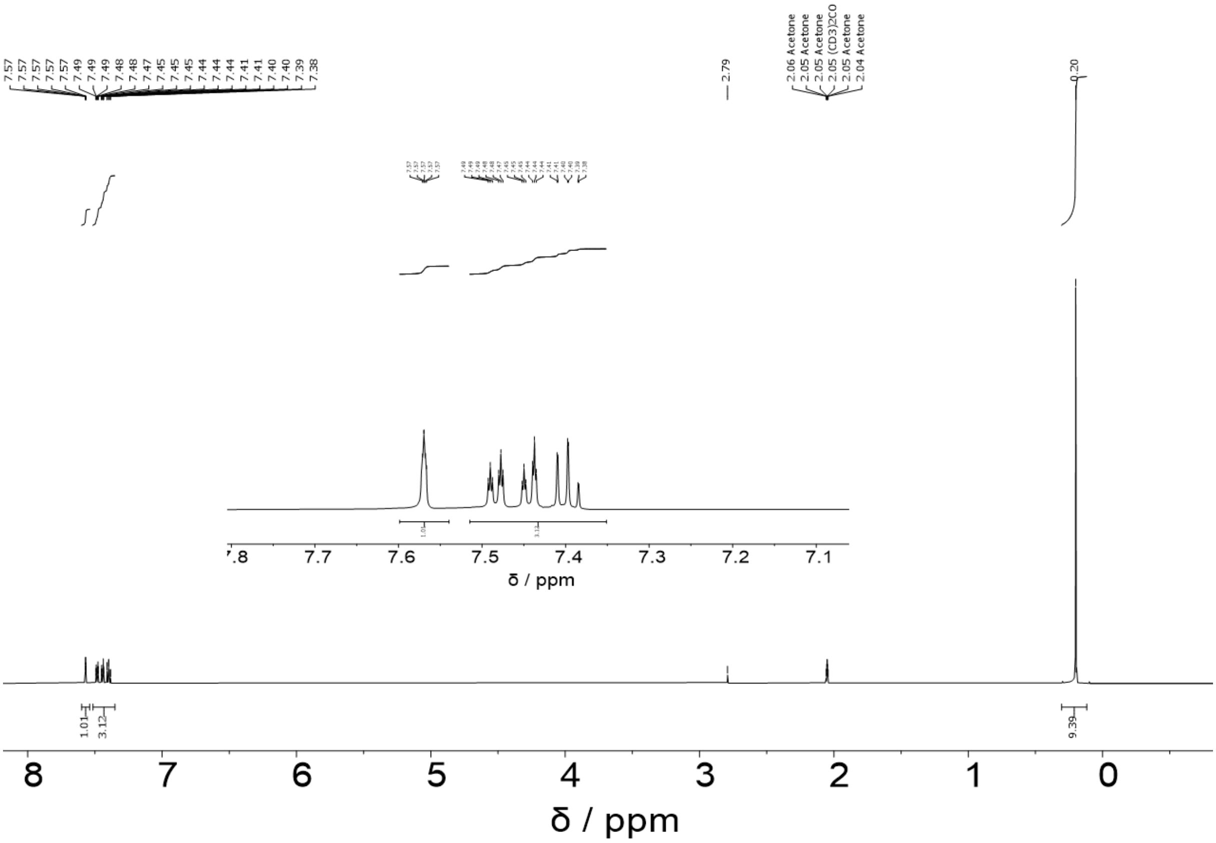

Figure S13. ^{1}H NMR of **2** (600 MHz, Acetone-d_6, 298K)

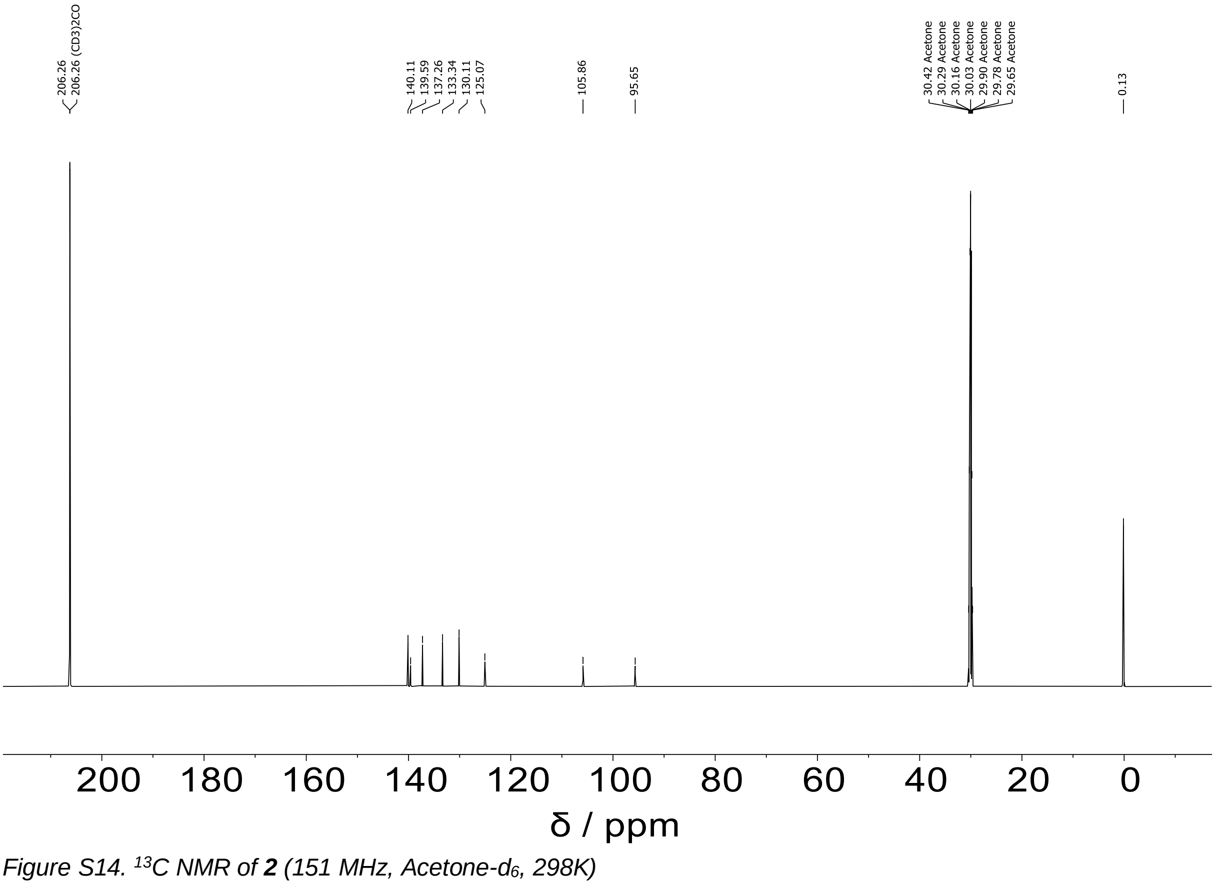

Figure S14. ^{13}C NMR of **2** (151 MHz, Acetone-d_6, 298K)

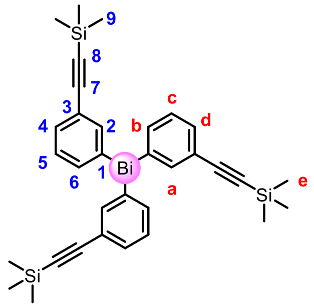

Compound **3** was prepared according to general procedure 2 and purified using silica gel flash column chromatography (5% DCM in Hexane). If required, the product can further be purified by dissolving it in minimal amounts of EtOAc and then adding MeOH, causing the pure product to crush out, which can then be isolated by vacuum filtration as a white solid (0.86 g, 1.19 mmol, 15%). **^{1}H NMR** (400 MHz, Acetone-d_6) δ 7.92 – 7.91 (m, 1H, H_a), 7.79 – 7.76 (m, 1H, H_b), 7.49 – 7.38 (m, 2H, $H_{c,d}$), 0.19 (s, 9H, H_e). **^{13}C NMR-{^{1}H}** (101 MHz, Acetone-d_6) δ 158.45 (s, C_1), 141.58 (s, C_2), 138.76 (s, C_6), 132.22 (s, C_4), 131.45 (s, C_5), 126.52 (s, C_3), 106.39 (s, C_7), 95.28 (s, C_8), 0.17 (s, C_9).

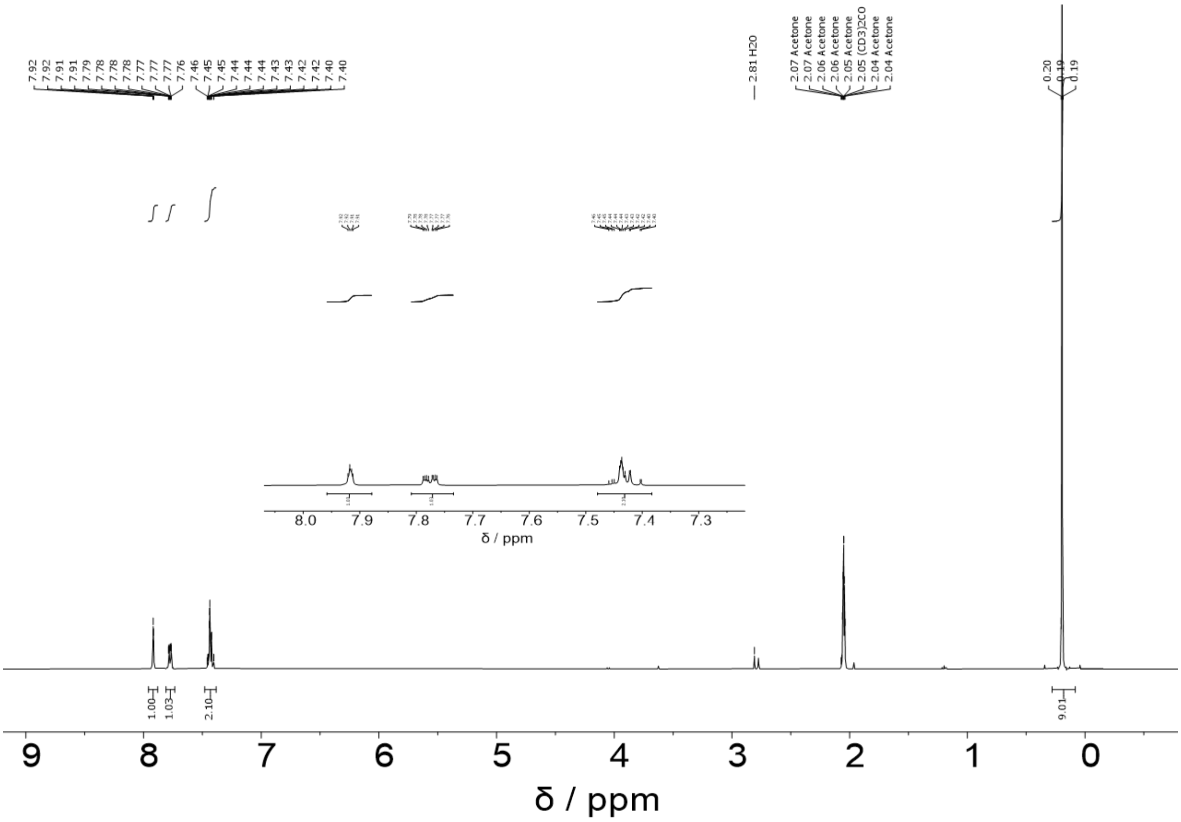

*Figure S15. ^{1}H NMR of **3** (400 MHz, Acetone-d_6, 298K)*

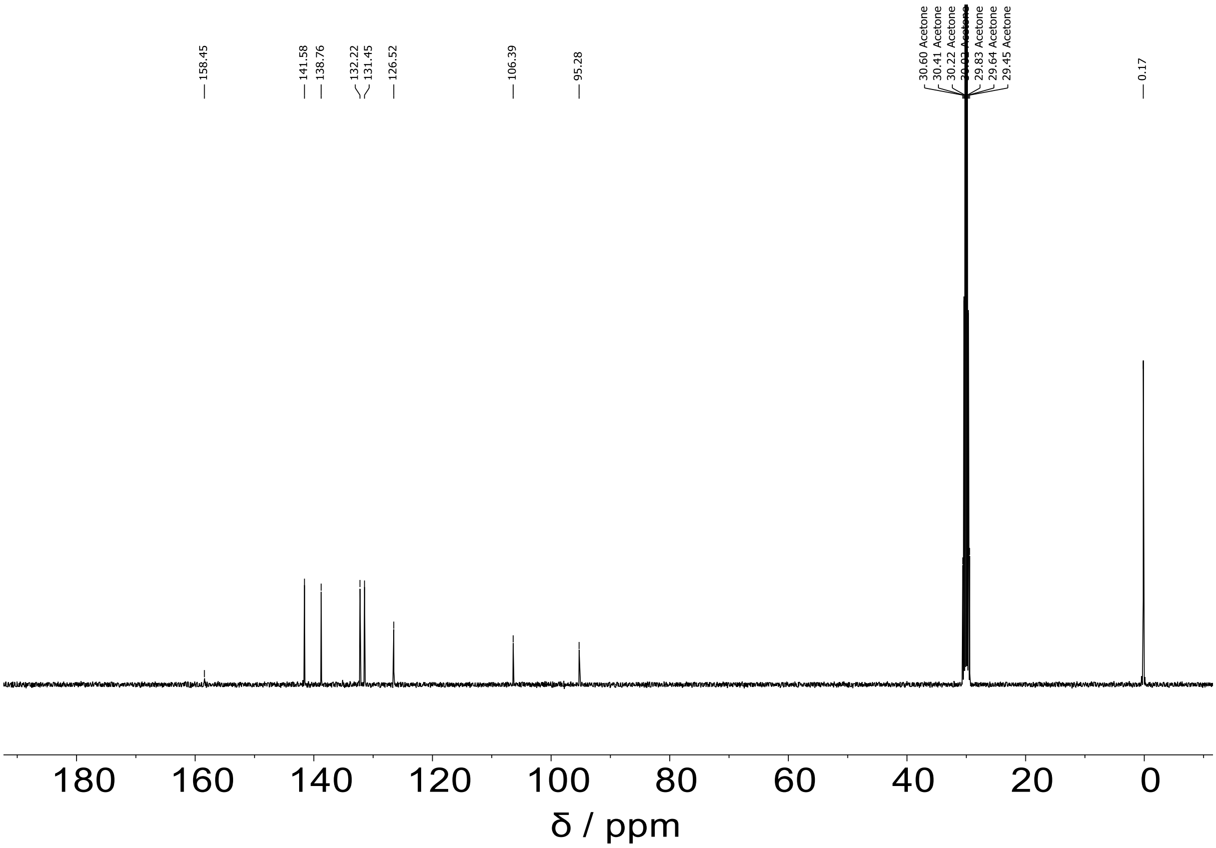

*Figure S16. ^{13}C NMR of **3** (101 MHz, Acetone-d$_6$, 298K)*

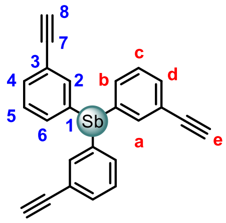

Compound **4** was prepared according to general procedure 3 to afford a white solid (0.70 g, 0.16 mmol, quantitative). **^{1}H NMR** (400 MHz, Acetone-d$_6$) δ 7.58 (t, J = 1.6 Hz, 1H, H$_{a}$), 7.53 – 7.48 (m, 2H, H$_{b,d}$), 7.42 (t, J = 7.5 Hz, 1H, H$_{c}$), 3.66 (s, 1H, H$_{e}$).**^{13}C NMR-{^{1}H}** (151 MHz, Acetone-d$_6$) δ 140.16 (s, C$_{2}$), 139.52 (s, C$_{1}$), 137.31 (s, C$_{6}$), 133.40 (s, C$_{4}$), 130.05 (s, C$_{5}$), 124.24 (s, C$_{3}$), 84.02 (s, C$_{7}$), 80.11 (s, C$_{8}$).

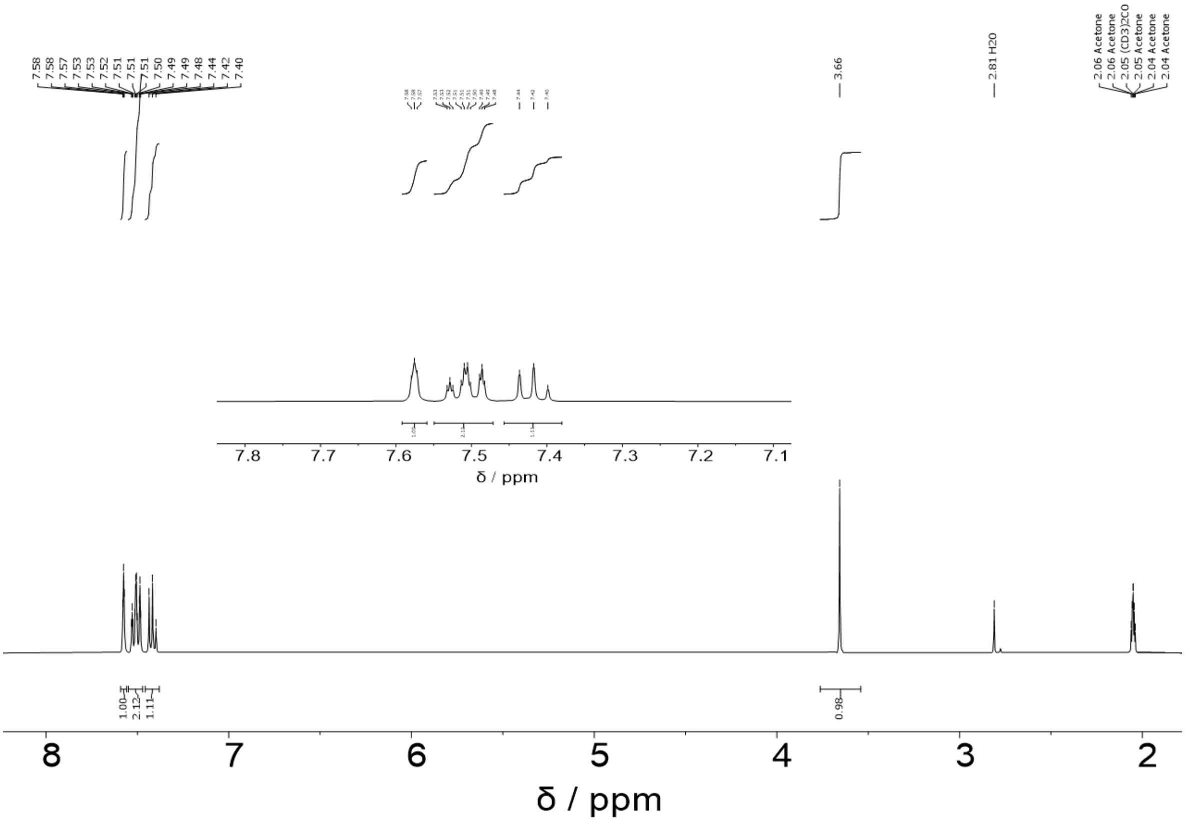

*Figure S17. ^{1}H NMR of **4** (400 MHz, Acetone-d$_6$, 298K)*

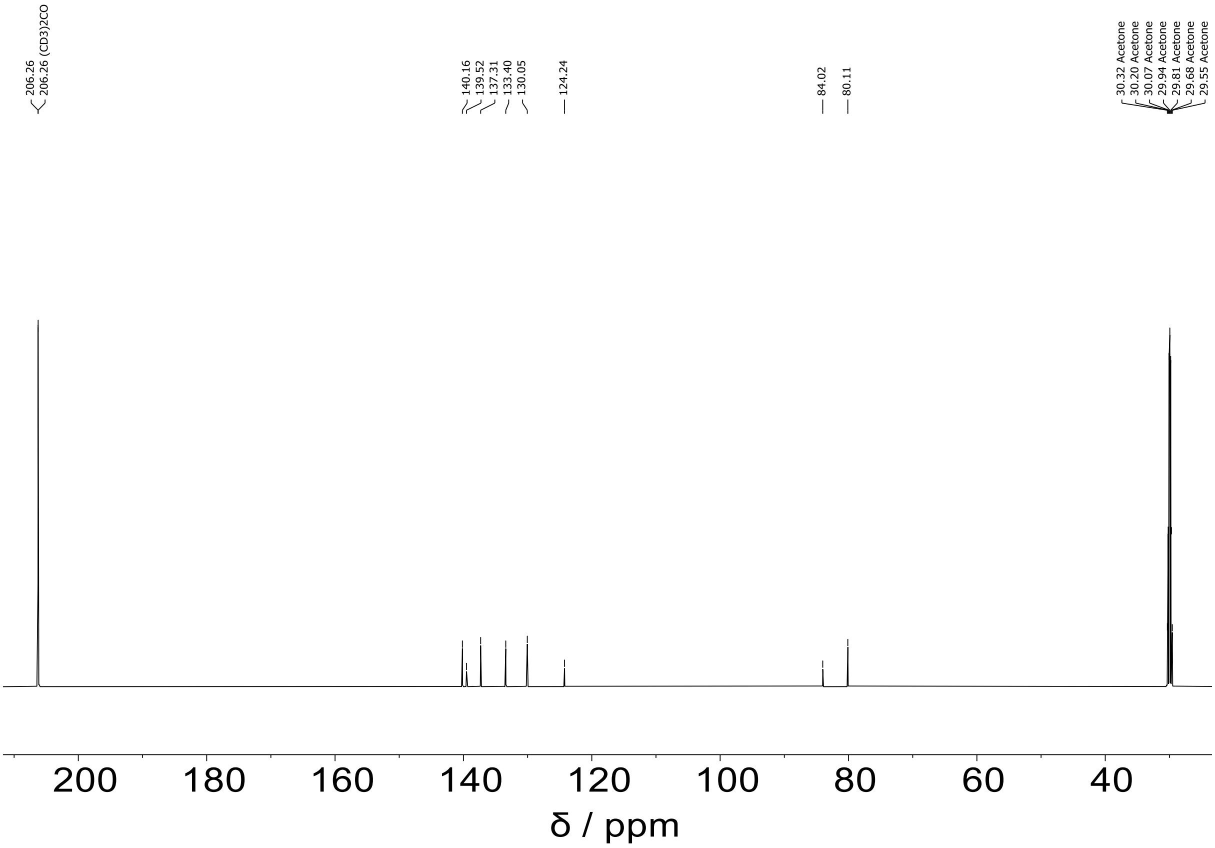

*Figure S18. ^{13}C NMR of **4** (151 MHz, Acetone-d$_6$, 298K)*

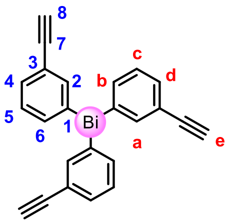

Compound **5** was prepared according to general procedure 3 on a 3.37 mmol (1 eq.) scale to afford a white solid (1.592 g, 3.12 mmol, 93 %). **^{1}H NMR** (400 MHz, Acetone) δ 7.94 (broad s, 1H, H_{a}), 7.81 (dt, J = 7.0, 1.4 Hz, 1H, H_{b}), 7.50 – 7.40 (m, 2H, $H_{c,d}$), 3.62 (s, 1H, H_{e}). **^{13}C NMR-{^{1}H}** (101 MHz, Acetone-d_6) δ 158.46 (s, C_{1}), 141.74 (s, C_{2}), 138.75 (s, C_{6}), 132.34 (s, C_{4}), 131.39 (s, C_{5}), 125.81 (s, C_{3}), 84.53 (s, C_{7}), 79.89 (s, C_{8}).

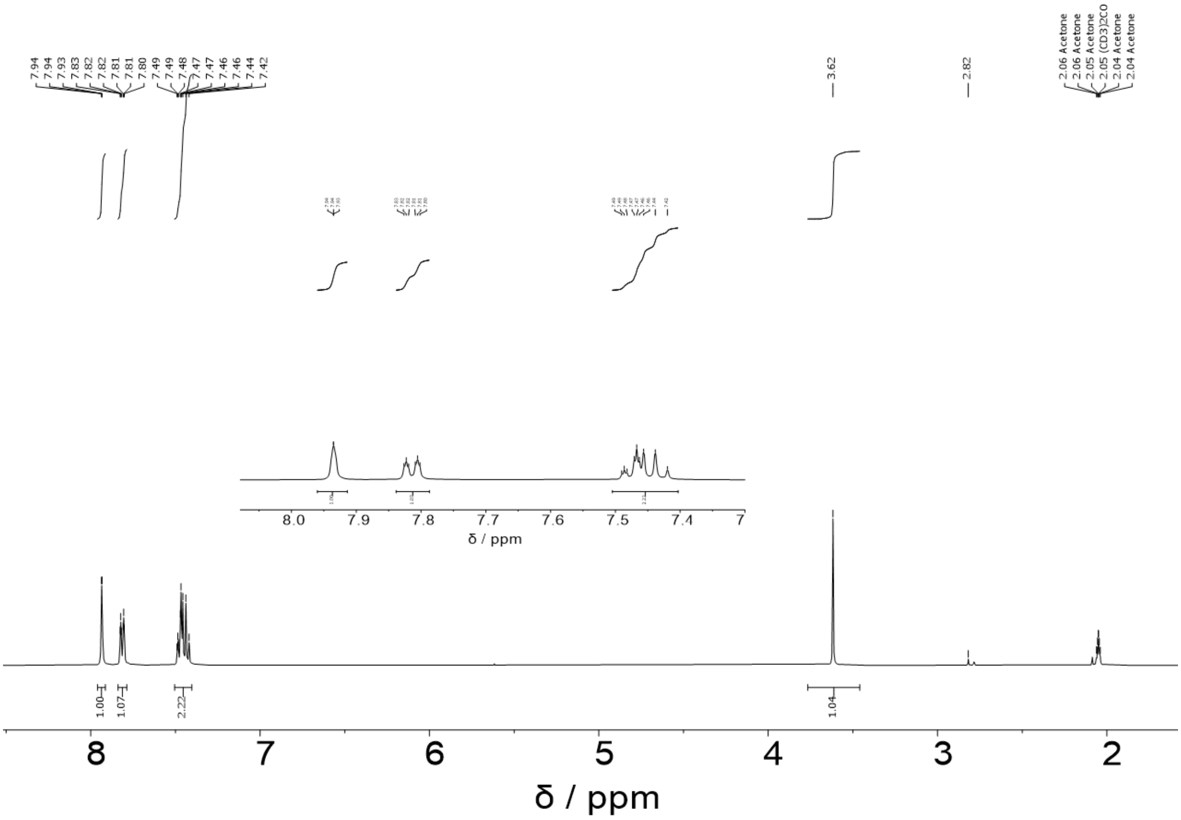

*Figure S19. ^{1}H NMR of **5** (400 MHz, Acetone-d_6, 298K)*

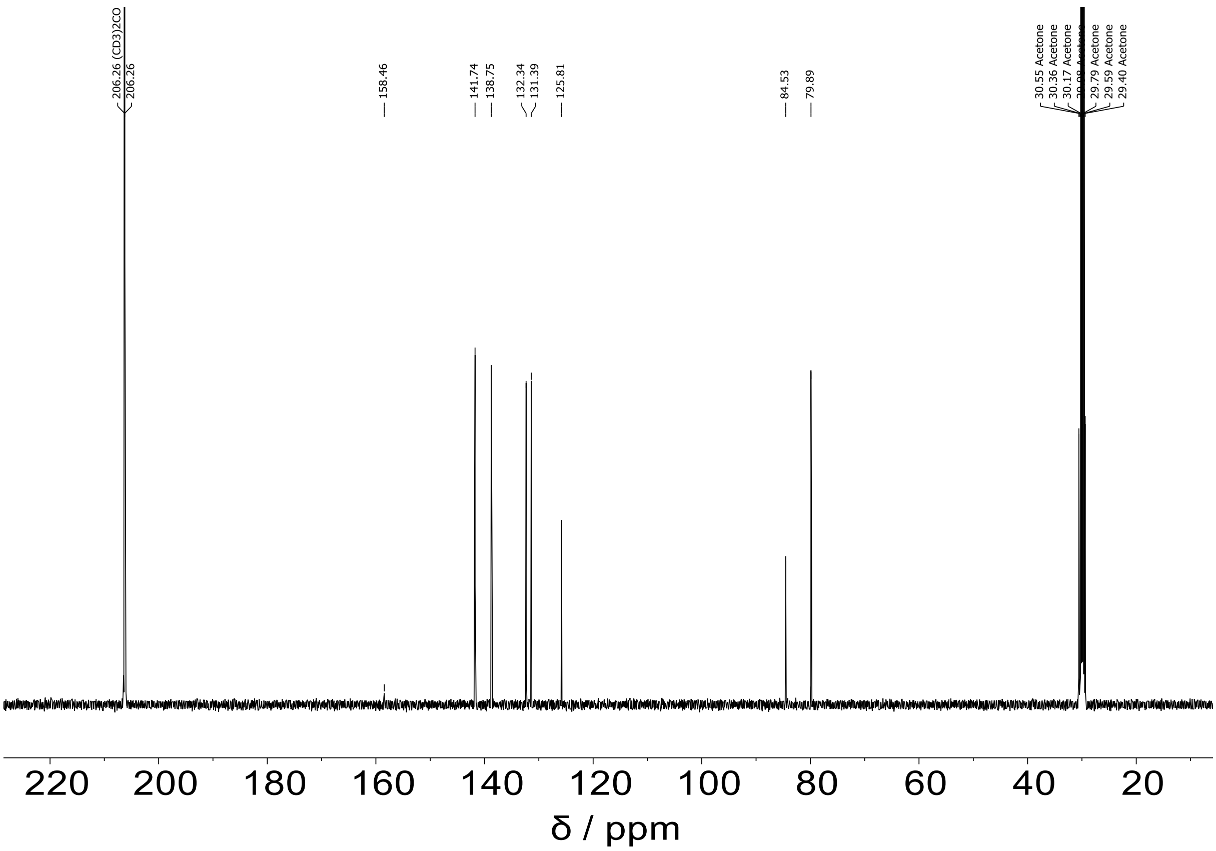

*Figure S20. ^{13}C NMR of **5** (101 MHz, Acetone-d$_6$, 298K)*

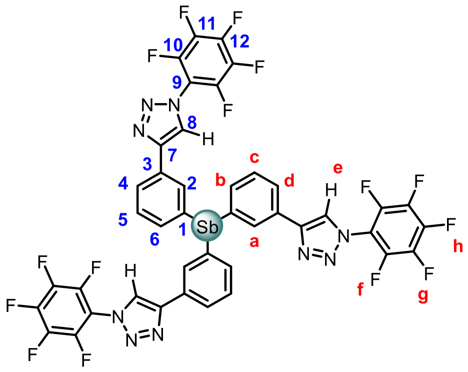

2·Sb^{PFP} was prepared according to general procedure 4 and purified using silica gel flash column chromatography (DCM) to yield a white solid (0.087 g, 0.083 mmol, 29 %). **^{1}H NMR** (400 MHz, Acetone-d$_6$) δ 8.83 (s, 3H, H$_e$), 8.32 (t, J = 1.6 Hz, 3H, H$_a$), 8.01 (dt, J = 7.5, 1.6 Hz, 3H, H$_d$), 7.62 – 7.49 (m, 6H, H$_{b,c}$). **^{13}C NMR-{^{1}H, ^{19}F}** (126 MHz, Acetone-d$_6$) δ 148.43 (s, C$_7$), 143.74 (s, C$_{10}$), 143.38 (s, C$_{12}$), 140.36 (s, C$_1$), 139.18 (s, C$_6$), 137.28 (s, C$_{11}$), 134.46 (s, C$_2$), 131.51 (s, C$_3$), 130.66 (s, C$_5$), 127.43 (s, C$_8$), 124.67 (s, C$_4$), 114.21 (s, C$_9$). **^{19}F NMR** (377 MHz, Acetone-d$_6$) δ -148.28 – -148.43 (m, 2F, F$_f$), -153.58 (t, J = 21.1 Hz, 1F, F$_h$), -162.93 – -163.13 (m, 2F, F$_g$). **HRESI-MS** (pos.) m/z 1074.0112, 1076.0121, 1077.0148 [MNa$^+$] calc. for [$C_{42}H_{15}F_{15}N_9Sb\cdot Na^+$] 1074.0165, 1076.0177, 1077.0202.

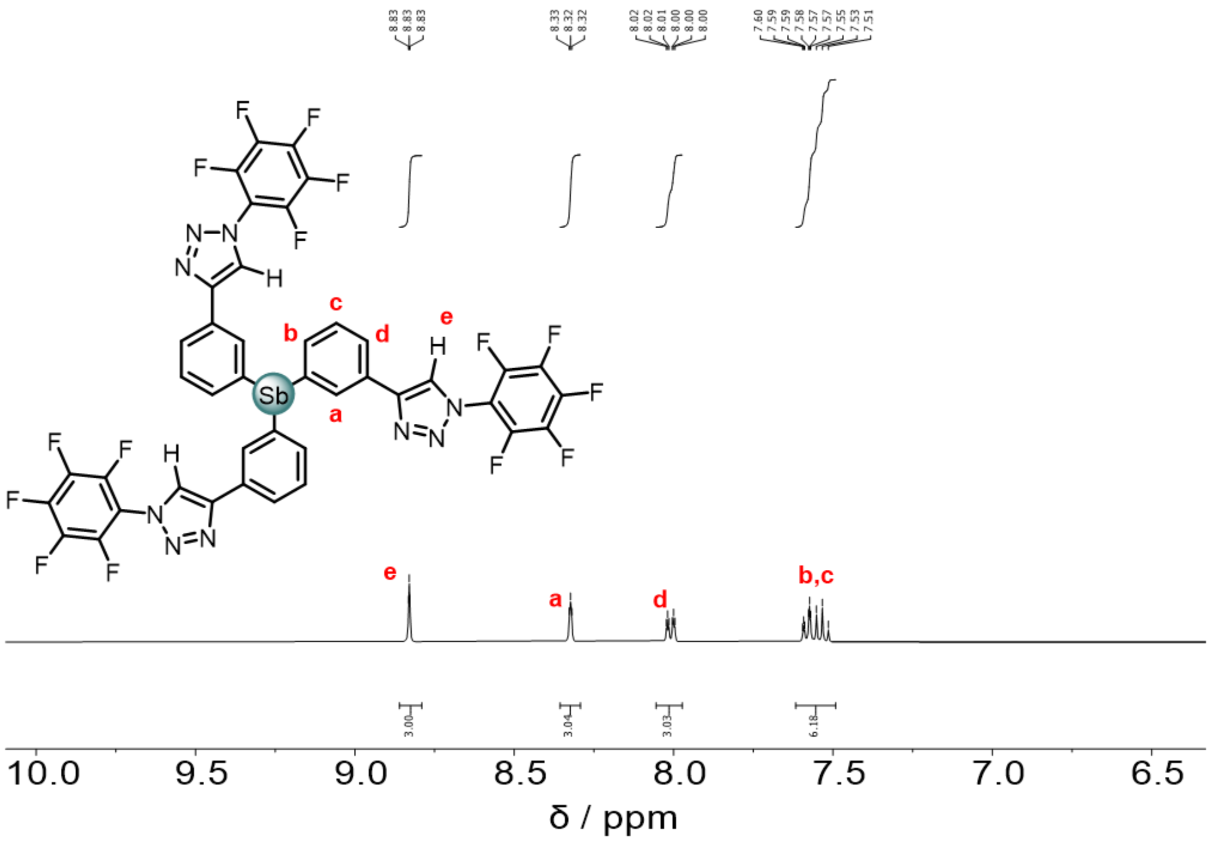

*Figure S21. ^{1}H NMR of **2·Sb^{PFP}** (400 MHz, Acetone-d$_6$, 298K)*

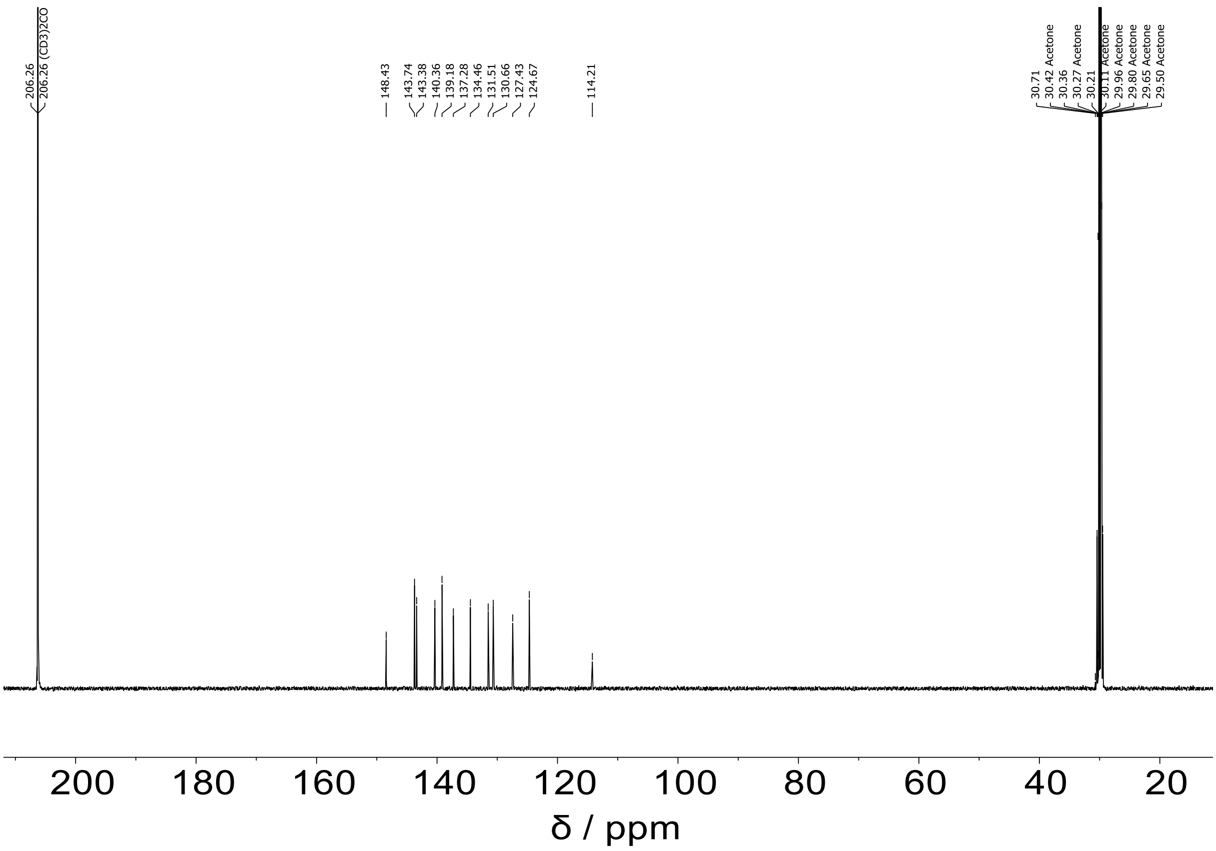

*Figure S22. ^{13}C NMR of **2·Sb^{PFP}** (126 MHz, Acetone-d$_6$, 298K)*

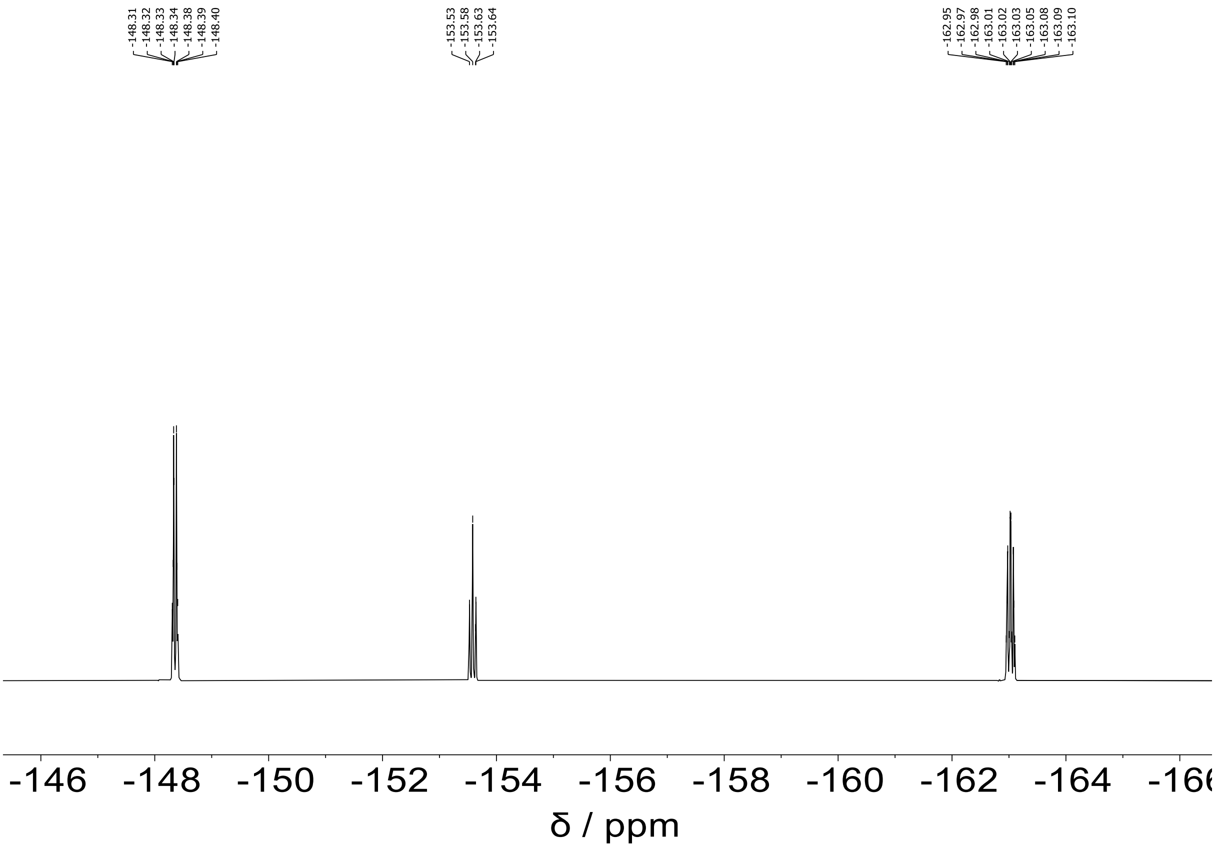

*Figure S23. ^{19}F NMR of **2·Sb^{PFP}** (377 MHz, Acetone-d$_6$, 298K)*

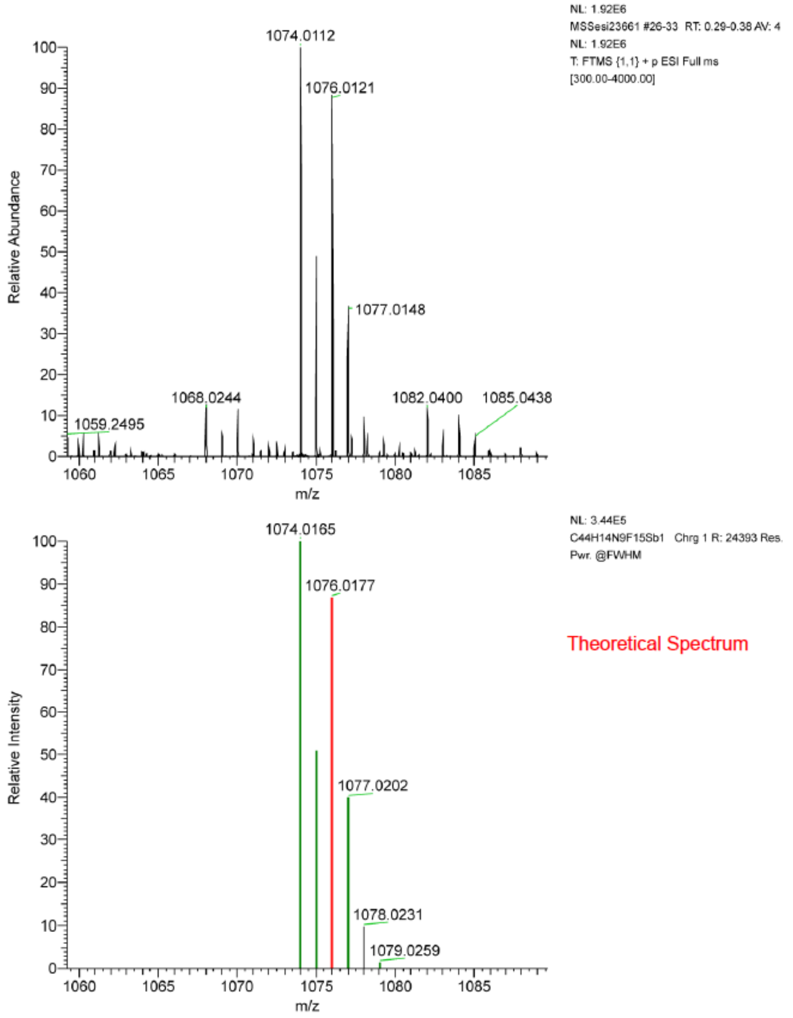

Figure S24. HRESI-MS spectrum of ***2·Sb^{PFP}***

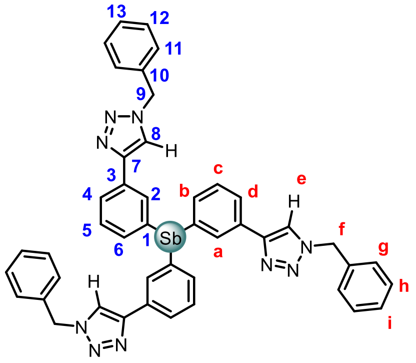

2·Sb^{Bz} was prepared according to general procedure 4 and purified using silica gel flash column chromatography (10% EtOAc in DCM) to yield a pale-yellow solid (0.139 g, 0.17 mmol, 58 %). **^{1}H NMR** (400 MHz, Acetone-d$_6$) δ 8.34 (s, 3H, H$_e$), 8.17 – 8.12 (m, 3H, H$_a$), 7.88 – 7.86 (m, 3H, H$_d$), 7.44 – 7.28 (m, 21H, H$_{b,c,g,h,i}$), 5.63 (s, 6H, H$_f$). **^{13}C NMR-{^{1}H}** (151 MHz, CDCl$_3$) δ 148.07 (s, C$_7$), 138.91 (s, C$_1$), 136.01 (s, C$_6$), 134.79 (s, C$_2$), 133.65 (s, C$_3$), 131.16 (s, C$_{10}$), 129.52 (s, C$_{11}$), 129.27 (s, C$_5$), 128.90 (s, C$_{12}$), 128.23 (s, C$_{13}$), 126.35 (s, C$_4$), 119.99 (s, C$_8$), 54.35 (s, C$_9$). **HRESI-MS** (pos.) m/z 824.2200, 826.2205, 827.2237, 826.2271 [MH$^+$] calc. for [$C_{45}H_{36}N_9Sb\cdot H^+$] 824.2205, 826.2216, 827.2242, 828.2270.

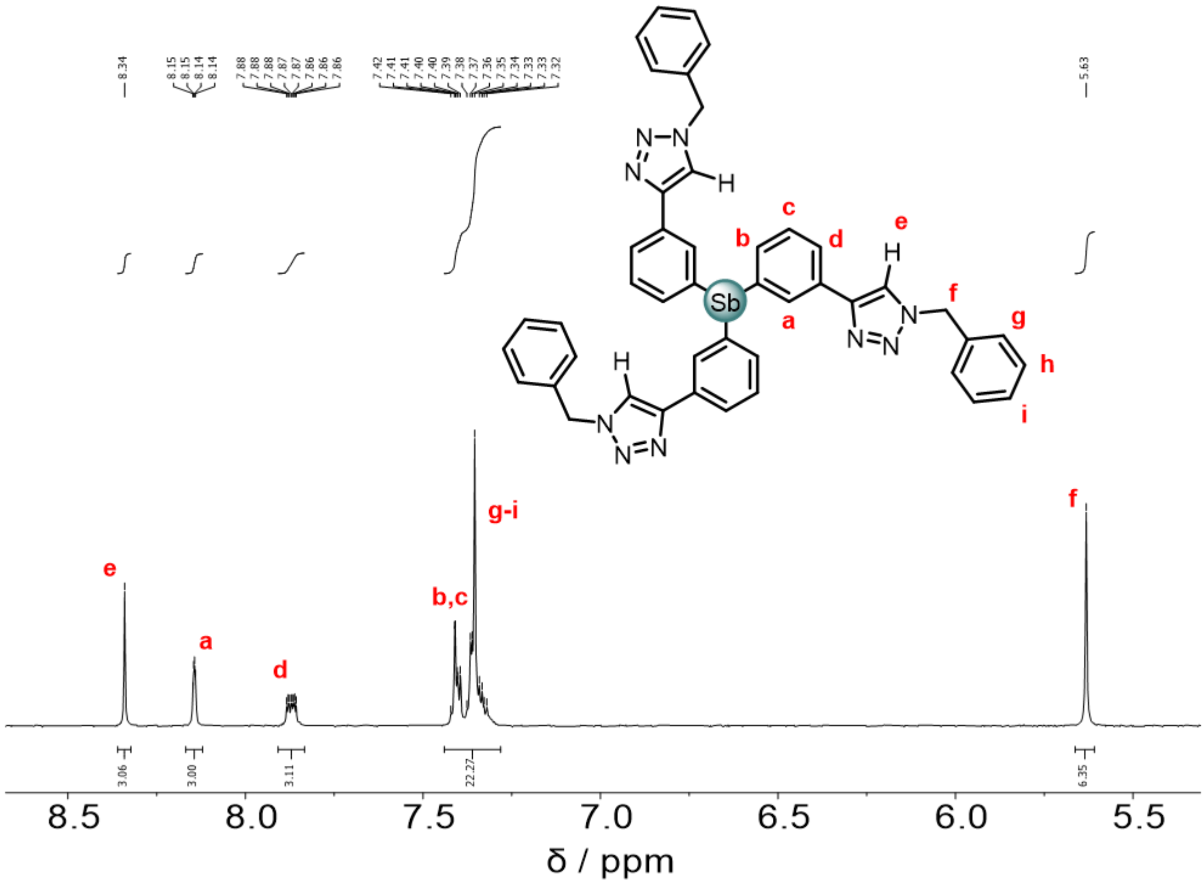

*Figure S25. ¹H NMR of **2·Sb^Bz** (400 MHz, Acetone-d₆, 298K)*

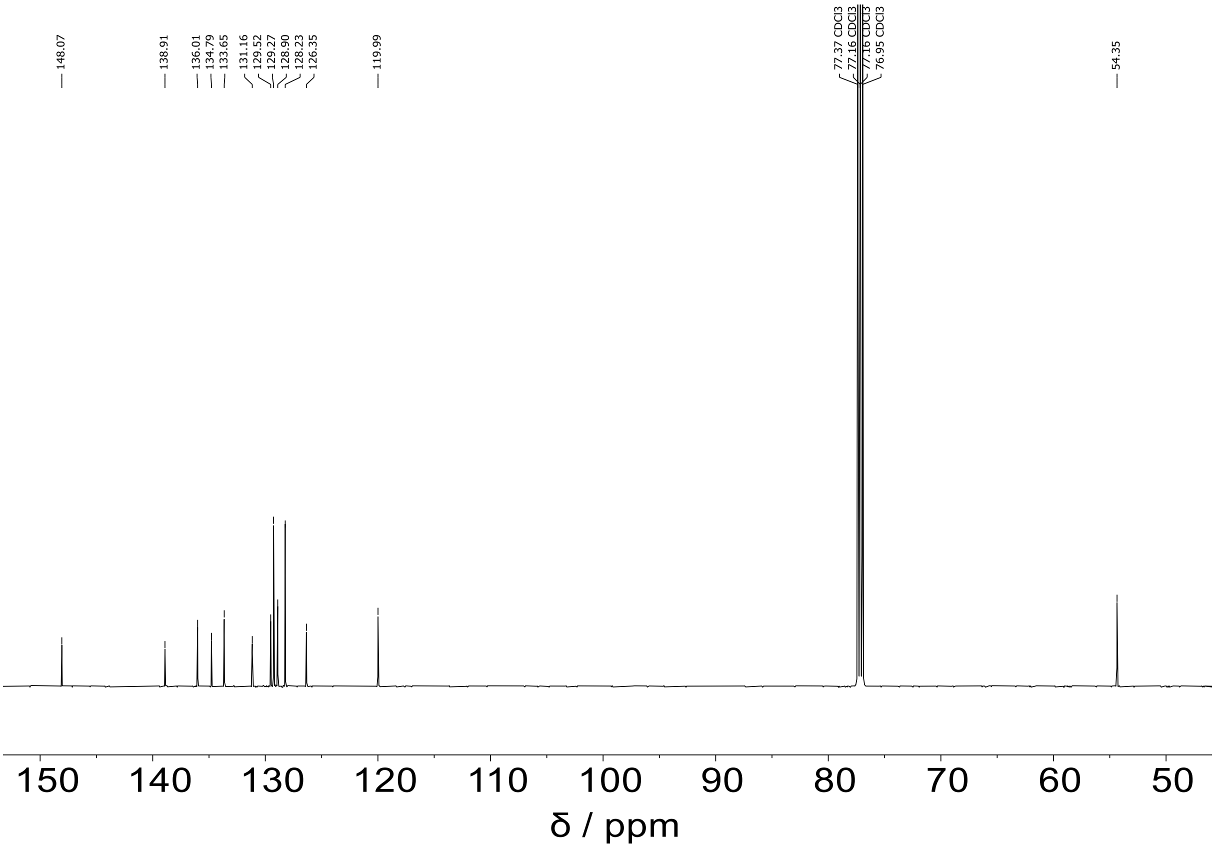

*Figure S26. ¹³C NMR of **2·Sb^Bz** (151 MHz, CDCl₃, 298K)*

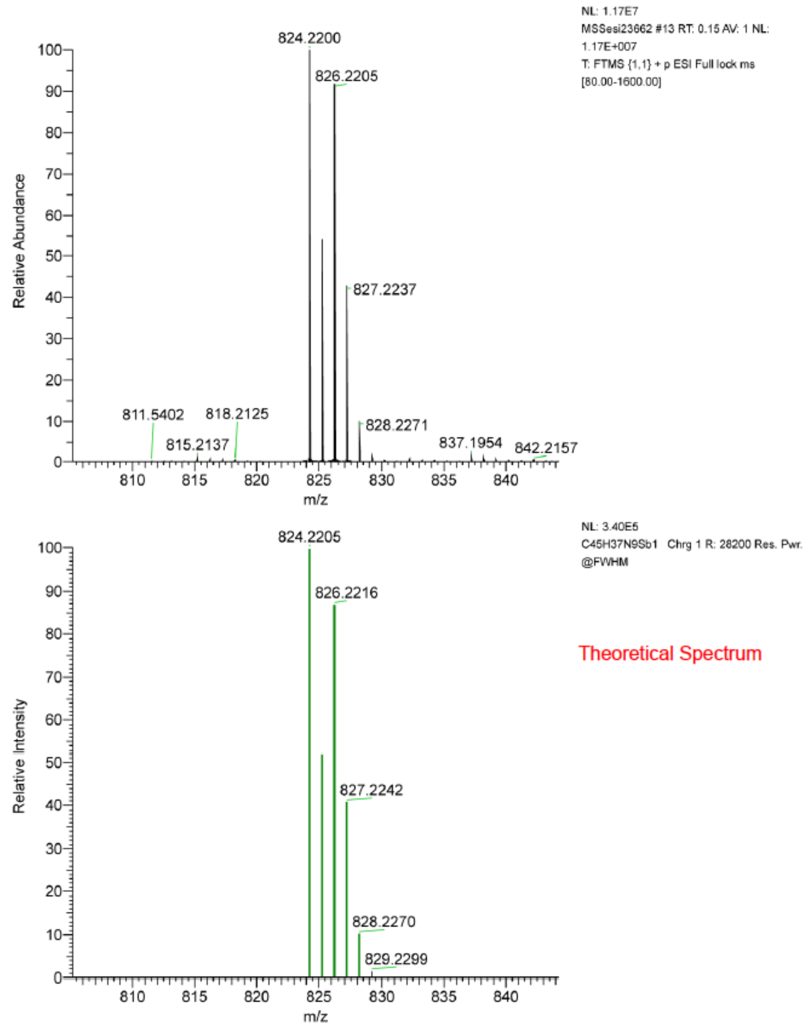

*Figure S27. HRESI-MS spectrum of **2·Sb**^{Bz}*

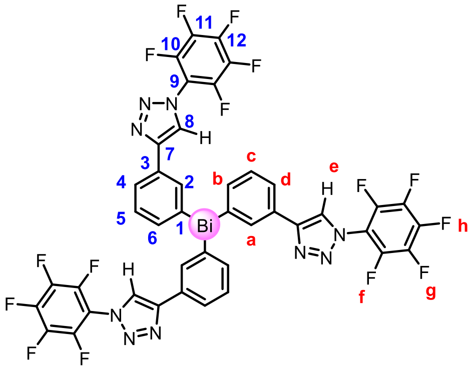

2·Bi^{PFP} was prepared according to general procedure 4 and purified using silica gel flash column chromatography (DCM) to yield a white solid (0.046 g, 0.040 mmol, 14 %). ^{1}**H NMR** (400 MHz, Acetone-d$_6$) δ 8.80 (s, 3H, H$_e$), 8.63 (t, J = 1.4 Hz, 3H, H$_a$), 7.97 – 7.91 (m, 6H, H$_{b,d}$), 7.56 (t, J = 7.6 Hz, 3H, H$_c$). ^{13}**C NMR-{**^{1}**H,** ^{19}**F}** (126 MHz, $CDCl_3$) δ 156.33 (s, C$_1$), 148.52 (s, C$_7$), 142.57 (s, C$_{10}$), 142.45 (s, C$_{12}$), 138.27 (s, C$_6$), 138.22 (s, C$_{11}$), 134.97 (s, C$_2$), 131.62 (s, C$_3$), 131.34 (s, C$_5$), 126.06 (s, C$_8$), 122.52 (s, C$_4$), 113.09 (s, C$_9$). ^{19}**F NMR** (377 MHz, Acetone-d$_6$) δ -148.28 – -148.43 (m, 2F, F$_f$), -153.66 (t, J = 21.1 Hz, 1F, F$_h$), -162.96 – -163.16 (m, 2F, F$_g$). **HRESI-MS** (pos.) m/z 1140.1079, 1141.1108, 1142.1140 [MH$^+$] calc. for [$C_{42}H_{15}F_{15}N_9Bi\cdot H^+$] 1140.1087, 1141.117, 1142.1147.

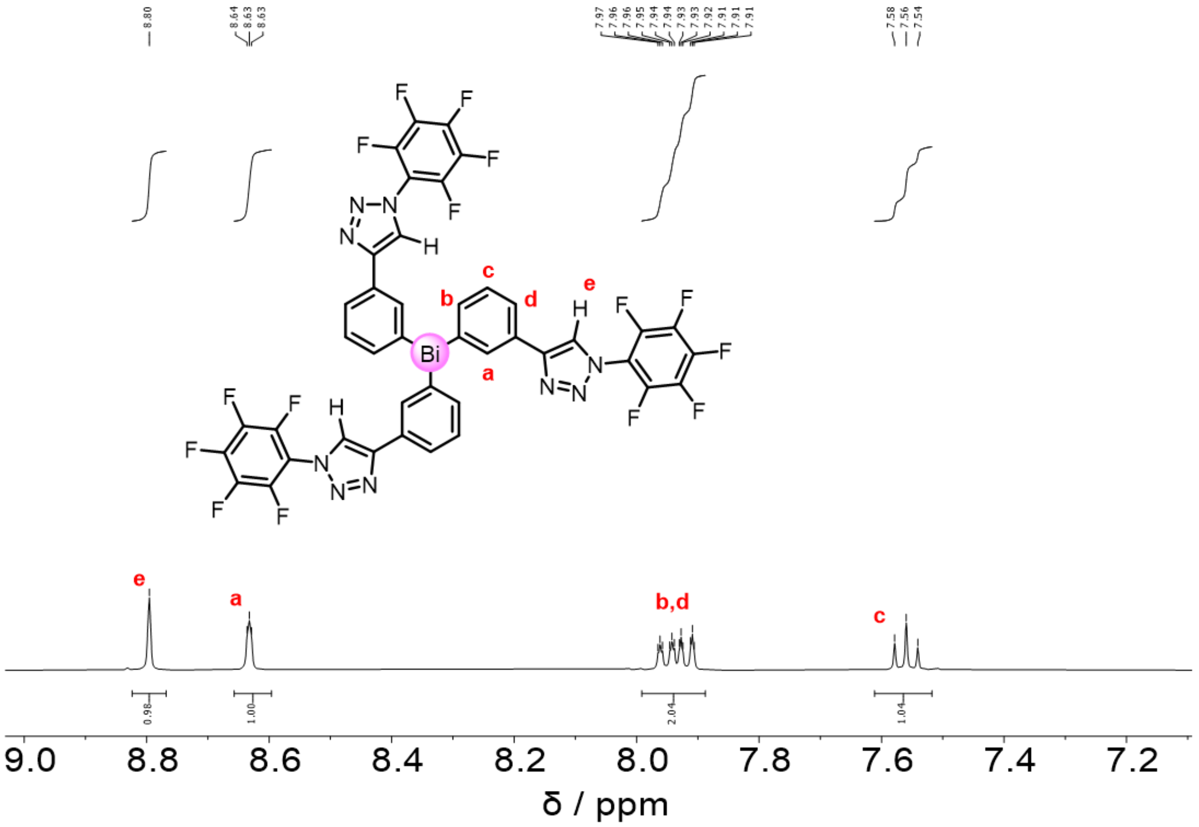

Figure S28. ^{1}*H NMR of* ***2·Bi***$^{\mathbf{PFP}}$ *(400 MHz, Acetone-d*$_6$*, 298K)*

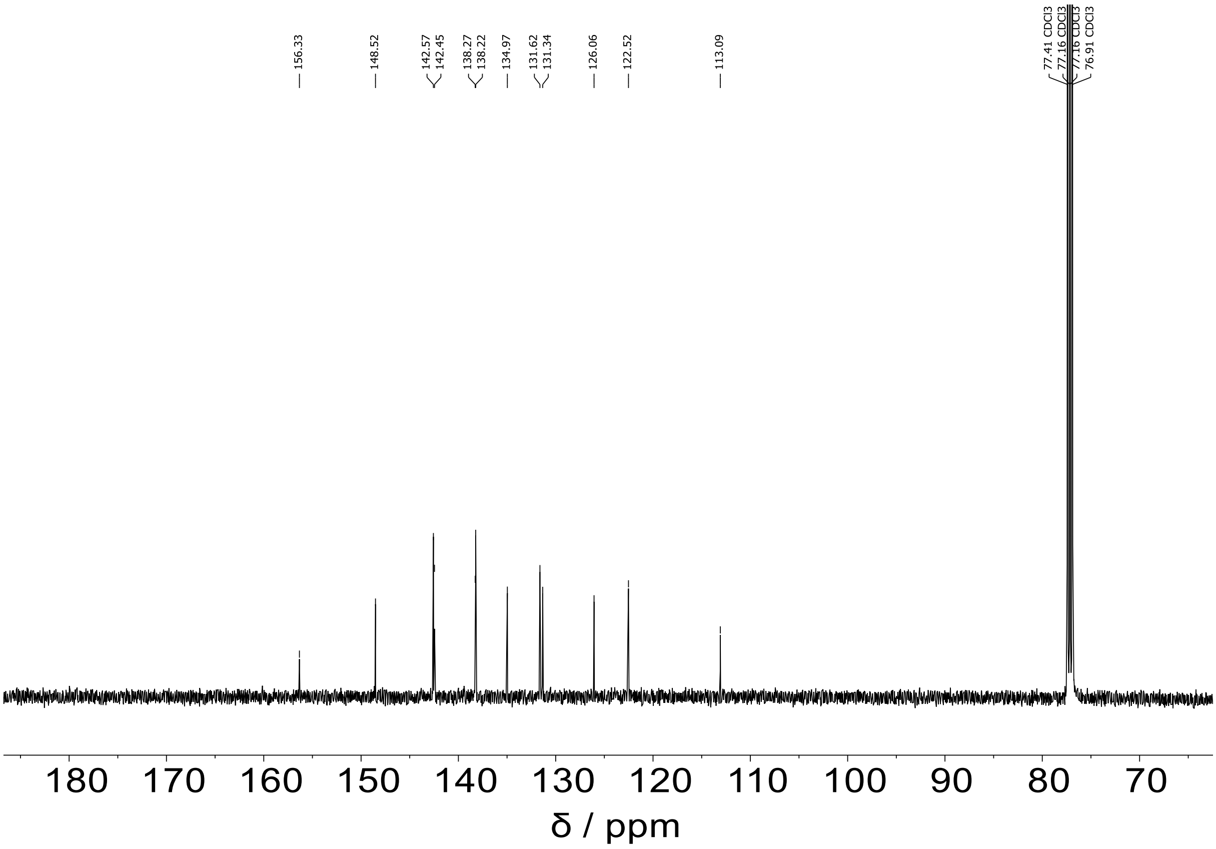

Figure S29. ^{13}*C NMR of* ***2·Bi***$^{\mathbf{PFP}}$ *(126 MHz, CDCl*$_3$*, 298K)*

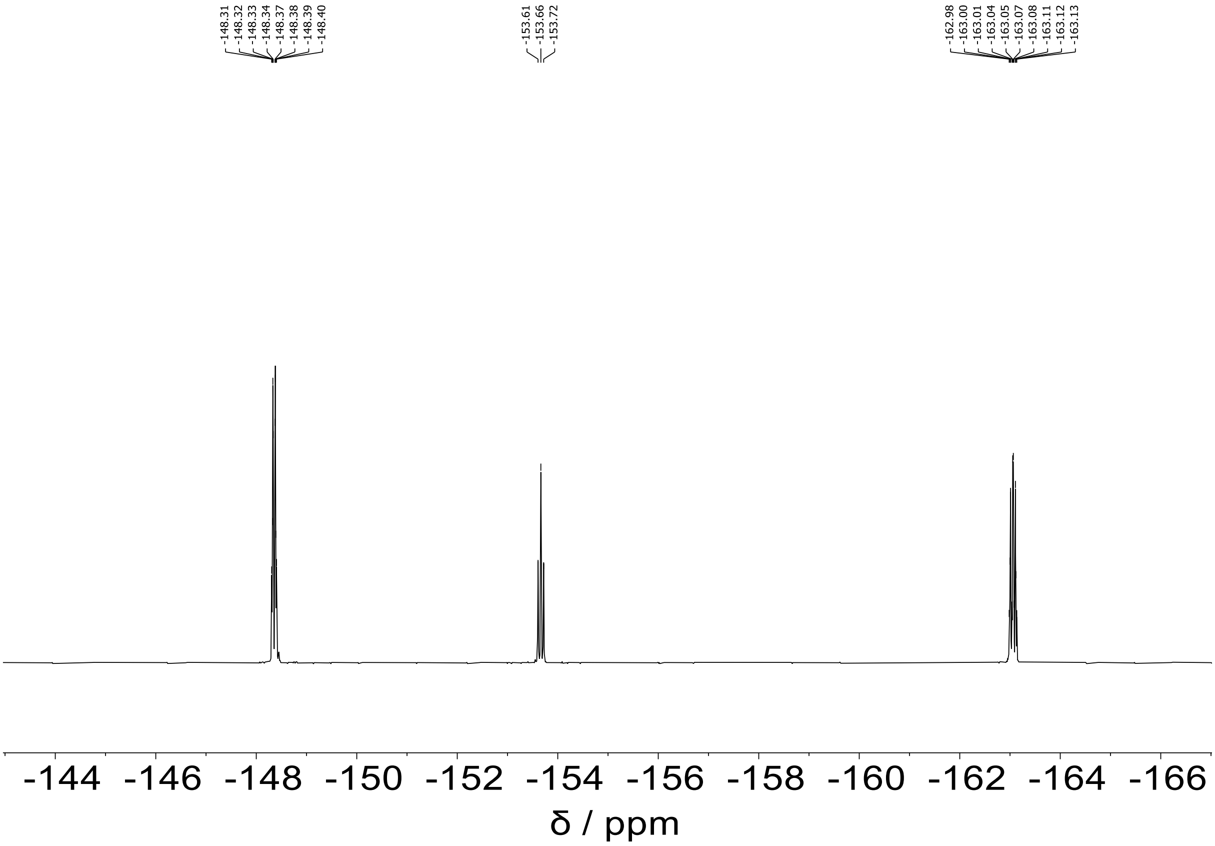

Figure S30. ^{19}*F NMR of* ***2·Bi***^{PFP} *(377 MHz, Acetone-*d_6*, 298K)*

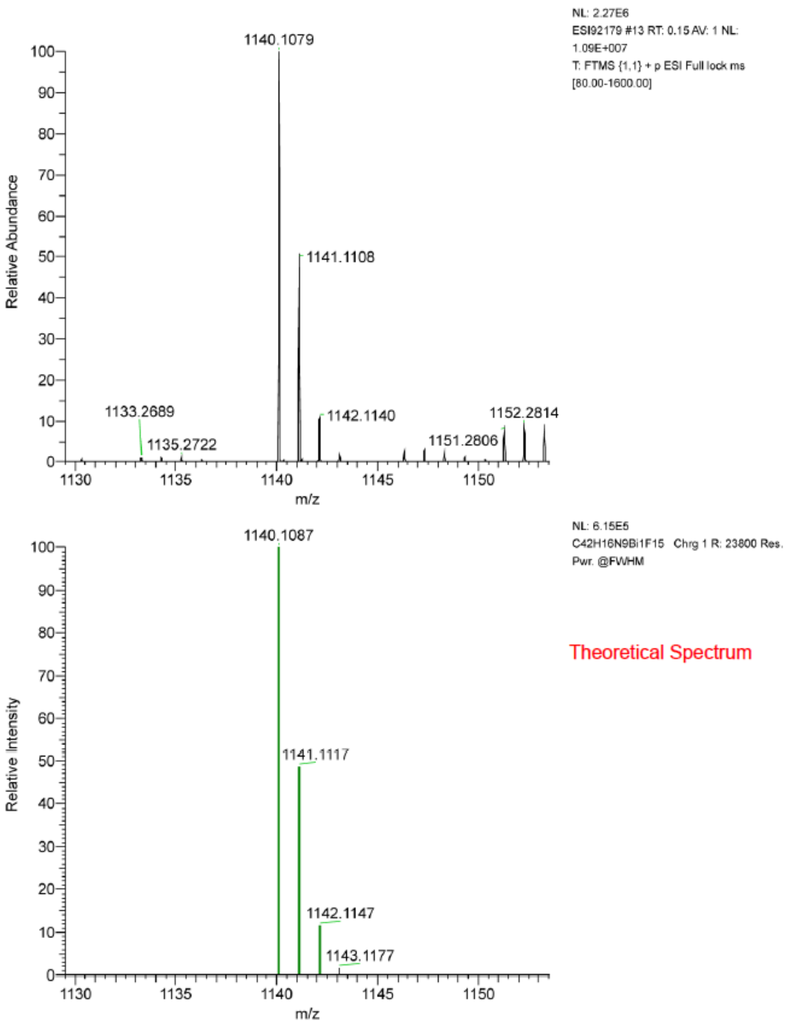

Figure S31. HRESI-MS spectrum of ***2·Bi***$^{\boldsymbol{PFP}}$

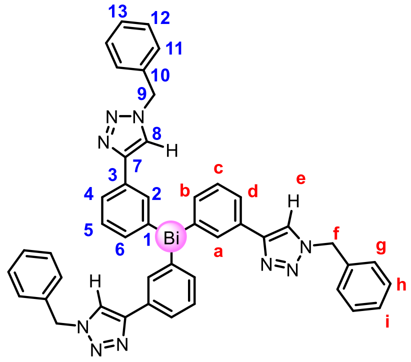

2·Bi$^{\mathbf{Bz}}$ was prepared according to general procedure 4 and purified using silica gel flash column chromatography (10 – 20 % EtOAc in DCM) to yield a white solid (0.16 g, 0.18 mmol, 61 %). ^{1}**H NMR** (400 MHz, Acetone) δ 8.44 (t, J = 1.4 Hz, 3H, H_a), 8.31 (s, 3H, H_e), 7.82 (dt, J = 7.8, 1.5 Hz, 3H, H_d), 7.75 (dt, J = 7.4, 1.2 Hz, 3H, H_b), 7.43 (t, J = 7.5 Hz, 3H, H_c), 7.38 – 7.28 (m, 15H, H_{g-i}), 5.63 (s, 6H, H_f). ^{13}**C NMR-{**^{1}**H}** (101 MHz, CDCl3) δ 155.97 (s, C_1), 148.30 (s, C_7), 137.51 (s, C_6), 134.81 (s, C_2), 134.72 (s, C_3), 132.56 (s, C_{10}), 131.14 (s, C_{11}), 129.22 (s, C_5), 128.84 (s, C_{12}), 128.20 (s, C_{13}), 125.45 (s, C_4), 120.03 (s, C_8), 54.29 (s, C_9). **HRESI-MS** (pos.) m/z 912.2953, 913.2988, 144.3031 [MH^+] calc. for [$C_{45}H_{36}N_9Bi{\cdot}H^+$] 912.2970, 913.3000, 914.3031.

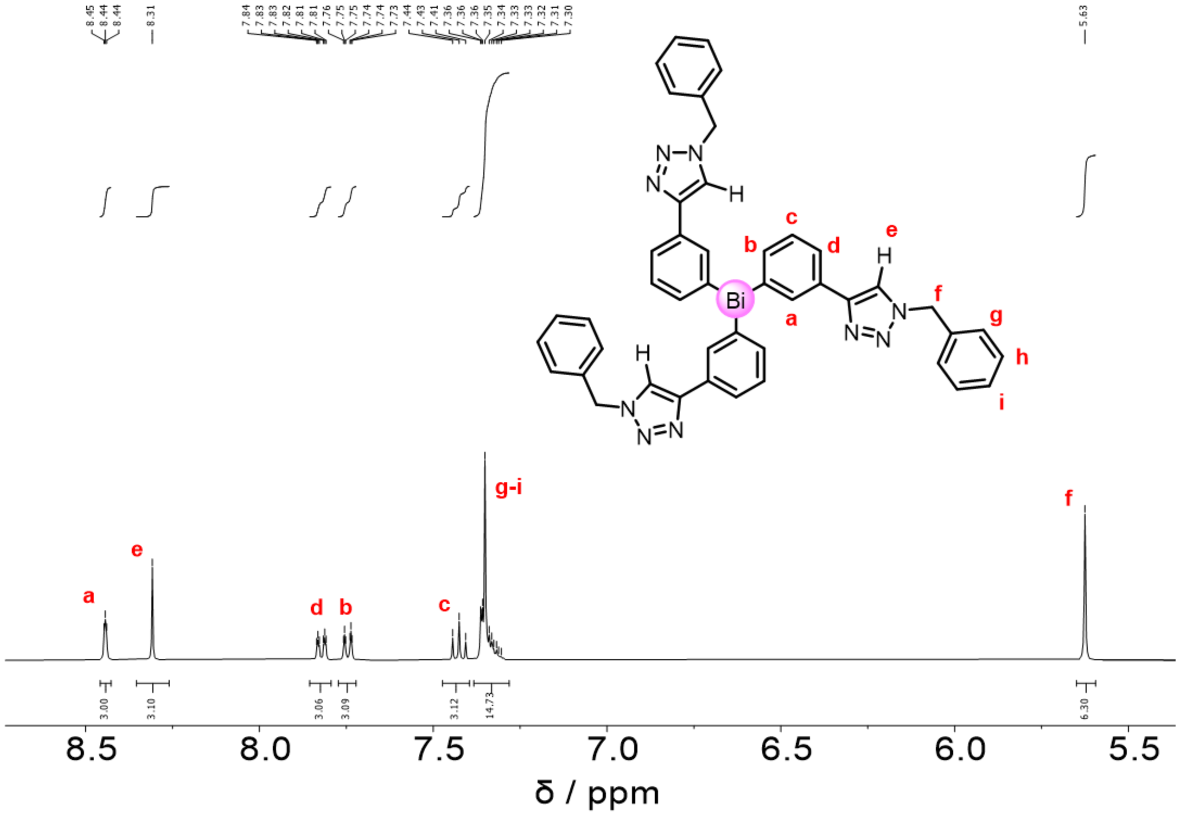

*Figure S32. ^{1}H NMR of **2·Bi^{Bz}** (400 MHz, Acetone-d$_6$, 298K)*

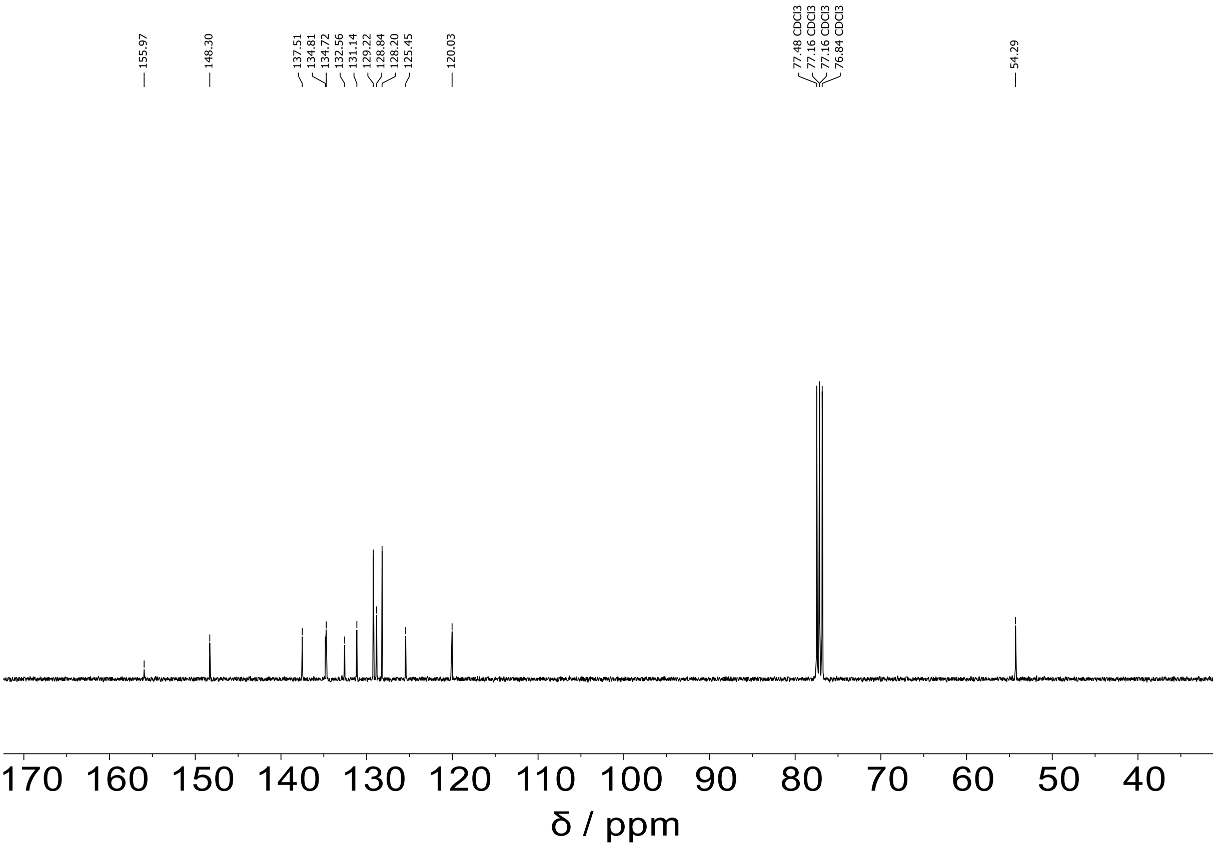

*Figure S33. ^{13}C NMR of **2·Bi^{Bz}** (101 MHz, Acetone-d$_6$, 298K)*

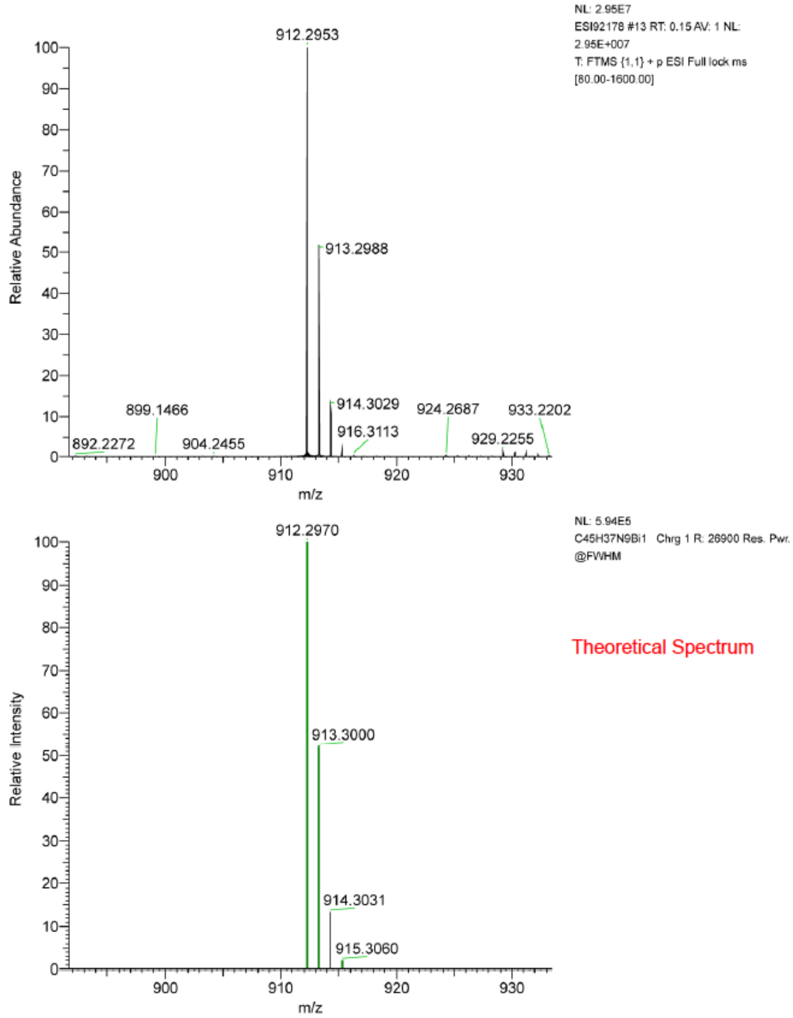

*Figure S34. HRESI-MS spectra of **2·Bi^{Bz}***

4 CHN Elemental Analysis

*Table S1 Summarised CHN analysis for **1·Sb^{R}** and **2·Sb^{R}** receptor series.*

**Analysis - 1·Sb^{2F}**			
**Element**	**Expected %**	**Found (1)**	**Found (2)**
**Carbon**	46.90	47.18	47.23
**Hydrogen**	1.97	1.73	1.72
**Nitrogen**	0	0	0
**Analysis - 1·Sb^{3F}**			
**Element**	**Expected %**	**Found (1)**	**Found (2)**
**Carbon**	41.98	42.18	42.22
**Hydrogen**	1.17	0.95	0.94
**Nitrogen**	0	0	0
**Analysis - 1·Sb^{2Cl}**			
**Element**	**Expected %**	**Found (1)**	**Found (2)**
**Carbon**	38.63	38.84	38.83
**Hydrogen**	1.62	1.44	1.51
**Nitrogen**	0	0	0
**Analysis - 1·Sb^{3F}**			
**Element**	**Expected %**	**Found (1)**	**Found (2)**
**Carbon**	32.61	33.72	33.65
**Hydrogen**	0.91	0.93	1.15
**Nitrogen**	0	0	0
**Analysis - 1·Sb^{2CF3}**			
**Element**	**Expected %**	**Found (1)**	**Found (2)**
**Carbon**	37.88	38.14	38.11
**Hydrogen**	1.19	1.06	1.08
**Nitrogen**	0	0	0
**Analysis - 1·Sb^{NO2}**			
**Element**	**Expected %**	**Found (1)**	**Found (2)**
**Carbon**	44.30	44.74	44.81
**Hydrogen**	2.48	2.35	2.30
**Nitrogen**	8.61	8.41	8.42
**Analysis - 1·Sb^{CN}**			
**Element**	**Expected %**	**Found (1)**	**Found (2)**
**Carbon**	58.92	59.49	59.48
**Hydrogen**	2.83	2.76	2.68
**Nitrogen**	9.82	9.35	9.45
**Analysis - 2·Sb^{Bz}**			
**Element**	**Expected %**	**Found (1)**	**Found (2)**
**Carbon**	65.55	64.82	65.12
**Hydrogen**	4.40	4.24	4.17
**Nitrogen**	15.29	14.93	14.91
**Analysis - 2·Sb^{PFP}**			
**Element**	**Expected %**	**Found (1)**	**Found (2)**
**Carbon**	47.94	48.77	48.59
**Hydrogen**	1.44	1.70	1.59
**Nitrogen**	11.98	11.60	11.61

*Table S2 Summarised CHN analysis for **1·Bi^{R}** and **2·Bi^{R}** receptor series.*

Analysis - 1·Bi^{2F}			
**Element**	**Expected %**	**Found (1)**	**Found (2)**
**Carbon**	39.43	39.65	39.64
**Hydrogen**	1.65	1.44	1.44
**Nitrogen**	0	0	0
**Analysis - 1·Bi^{3F}**			
**Element**	**Expected %**	**Found (1)**	**Found (2)**
**Carbon**	35.90	36.38	36.84
**Hydrogen**	1.00	0.80	0.68
**Nitrogen**	0	0	0
**Analysis - 1·Bi^{2Cl}**			
**Element**	**Expected %**	**Found (1)**	**Found (2)**
**Carbon**	33.42	34.62	34.56
**Hydrogen**	1.40	1.46	1.42
**Nitrogen**	0	0	0
**Analysis - 1·Bi^{3Cl}**			
**Element**	**Expected %**	**Found (1)**	**Found (2)**
**Carbon**	28.82	29.20	29.22
**Hydrogen**	0.81	0.80	0.71
**Nitrogen**	0	0	0
**Analysis - 1·Bi^{2CF3}**			
**Element**	**Expected %**	**Found (1)**	**Found (2)**
**Carbon**	33.98	34.21	34.27
**Hydrogen**	1.07	1.01	0.90
**Nitrogen**	0	0	0
**Analysis - 1·Bi^{NO2}**			
**Element**	**Expected %**	**Found (1)**	**Found (2)**
**Carbon**	37.58	-[a]	-[a]
**Hydrogen**	2.10	-[a]	-[a]
**Nitrogen**	7.30	-[a]	-[a]
**Analysis - 1·Bi^{CN}**			
**Element**	**Expected %**	**Found (1)**	**Found (2)**
**Carbon**	48.95	49.03	49.98
**Hydrogen**	2.35	2.19	2.35
**Nitrogen**	8.15	8.00	8.09
**Analysis - 2·Bi^{Bz}**			
**Element**	**Expected %**	**Found (1)**	**Found (2)**
**Carbon**	59.28	59.56	59.78
**Hydrogen**	3.98	3.98	4.04
**Nitrogen**	13.83	13.43	13.48
**Analysis - 2·Bi^{PFP}**			
**Element**	**Expected %**	**Found (1)**	**Found (2)**
**Carbon**	44.27	44.24	44.28
**Hydrogen**	1.33	1.16	1.21
**Nitrogen**	11.06	11.01	10.93

[a] Reliable data could not be obtained due to instability of the compound.

5 Titration Data

5.1 General Procedure

^{1}H-NMR titrations were recorded on a Bruker Avance III NMR equipped with a 11.75T magnet. Chemical shifts are quoted in parts per million relative to the residual solvent peak. All ^{1}H-NMR anion titrations were performed in THF-d_8 at 298 K. Aliquots of the respective anion were added to a solution of the receptor, such that the receptor solution concentration remained constant. Spectra were recorded at 0.00, 0.20, 0.39, 0.59, 0.78, 0.96, 1.15, 1.33, 1.50, 1.68, 1.85, 2.27, 2.68, 3.45, 4.17, 5.47, 7.14 and 9.38 equivalents.

For TBACl and TBABr ^{1}H-NMR titrations, the host concentration was 1mM with a TBACl or TBABr concentration of 25 mM. All other ^{1}H-NMR titrations were performed at host concentrations of 0.5 mM with TBA salt concentrations of 12.5 mM. Titration isotherms were analysed using Bindfit.[11, 12]

An example experimental titration procedure for **2·Bi**$^{\mathbf{PFP}}$ is detailed below;

i. Required [**2·Bi**$^{\mathbf{PFP}}$] = 1 mM and [TBACl] = 25 mM
ii. Weighed amount of **2·Bi**$^{\mathbf{PFP}}$: 2.01 mg → Dissolve in 1.76 ml of THF-d_8 → 0.5 ml syringed into NMR tube.
iii. Weighed amount of TBACl : 8.24 mg → Dissolve in 1.19 ml of [**2·Bi**$^{\mathbf{PFP}}$] = 1 mM in THF-d_8 → aliquots of this [TBACl] = 25 mM solution added to the [**2·Bi**$^{\mathbf{PFP}}$] = 1 mM solution in NMR tube and spectra recorded.

Bindfit software operates (1:1 host guest stoichiometry model) using the following equation where [HG] is the concentration of host guest complex, $[G]_0$ and $[H]_0$ are the total concentrations of the guest and host respectively and K_a is the equilibrium constant:

$$[HG] = \frac{1}{2}\left([G]_0 + [H]_0 + \frac{1}{K_a}\right) - \sqrt{\left([G]_0 + [H]_0 + \frac{1}{K_a}\right)^2 + 4[H]_0[G]_0}$$

For NMR experiments the observed change in chemical shift, Δδ, is directly proportional to the change $\delta_{\Delta HG}$ in the NMR resonances between the host guest complex (δ_{HG}) and the free host (δ_H) multiplied by the fraction of the complex HG:

$$\Delta\delta = \delta_{\Delta HG}\left(\frac{[HG]}{[H]_0}\right)$$

The expected changes from a titration experiment can therefore be described by two known ($[G]_0$ and $[H]_0$) and two unknown (K_a and $\delta_{\Delta HG}$) parameters. The two unknown parameters are obtained from non-linear regression of the experimental data, wherein initial guesses of K_a and $\delta_{\Delta HG}$ are used to calculate Δδ and compare it to the experimentally obtained value and are varied until a good fit is obtained.

5.2 Bismuth Triaryl Compounds

Table S3. Chloride anion association constants for bismuth receptors. K_a(M^{-1}) values calculated using Bindfit software using a 1:1 stoichiometric host-guest binding model. Errors (±) are in parentheses. Solvent = THF-d_8. T = 298 K.

	**K_a / M^{-1}**
**1·Bi^{CN}**	76(1)
**1·Bi^{2F}**	152(2)
**1·Bi^{2Cl}**	213(1)
**1·Bi^{NO2}**	227(3)
**1·Bi^{3F}**	580(7)
**1·Bi^{3Cl}**	716(8)

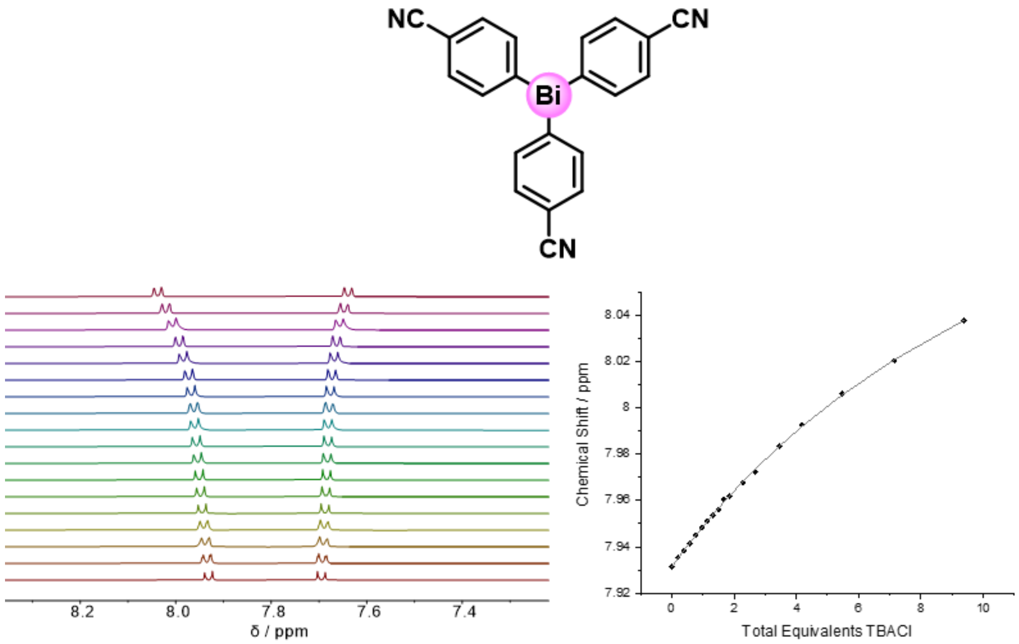

*Figure S35. Stacked ^{1}H-NMR TBACl titration (left) and chloride binding isotherm with dots representing experimental data and solid lines representing the fitted binding isotherm (right) for **1·Bi^{CN}**. Solvent = THF-d_8. T = 298 K.*

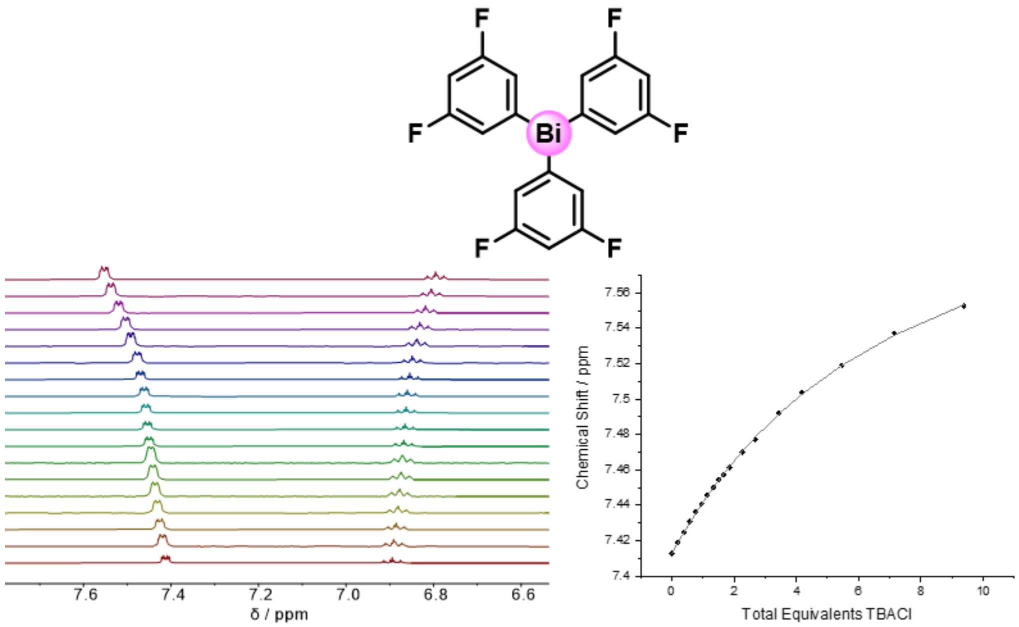

*Figure S36. Stacked ^{1}H-NMR TBACl titration (left) and chloride binding isotherm with dots representing experimental data and solid lines representing the fitted binding isotherm (right) for **1·Bi^{2F}**. Solvent = THF-d_8. T = 298 K.*

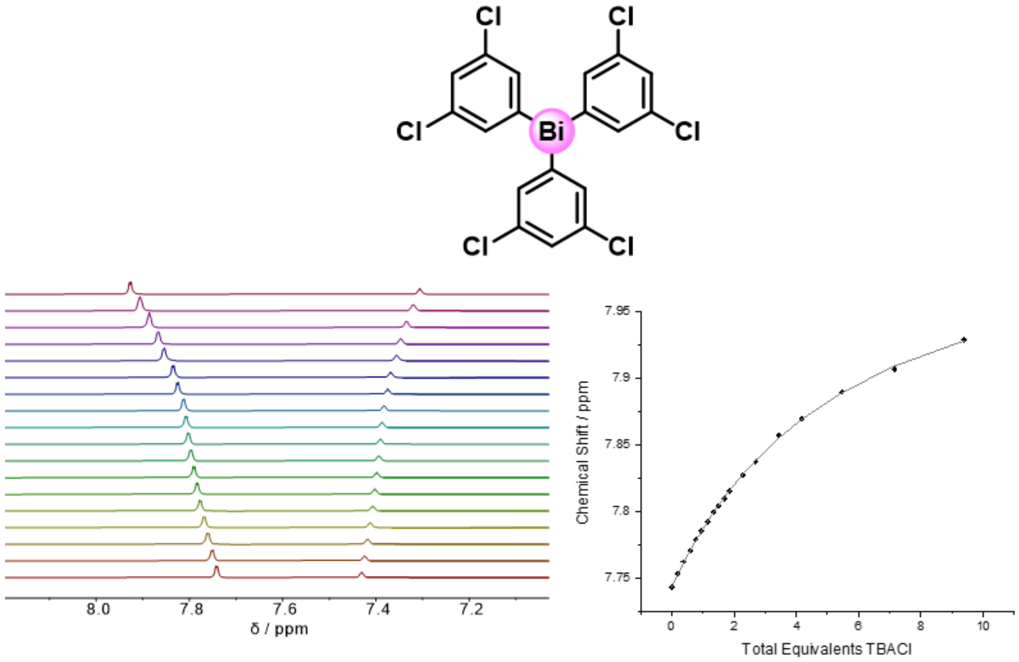

*Figure S37. Stacked ^{1}H-NMR TBACl titration (left) and chloride binding isotherm with dots representing experimental data and solid lines representing the fitted binding isotherm (right) for **1·Bi^{2Cl}**. Solvent = THF-d_8. T = 298 K.*

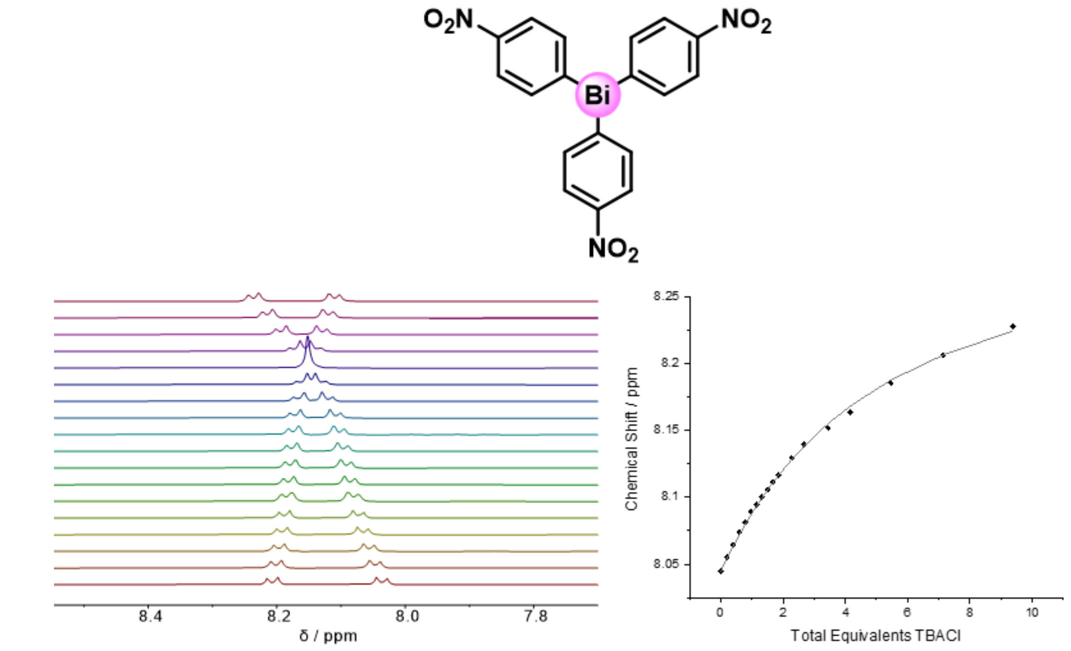

*Figure S38. Stacked ^{1}H-NMR TBACl titration (left) and chloride binding isotherm with dots representing experimental data and solid lines representing the fitted binding isotherm (right) for **1·Bi**^{NO2}. Solvent = THF-d_8. T = 298 K.*

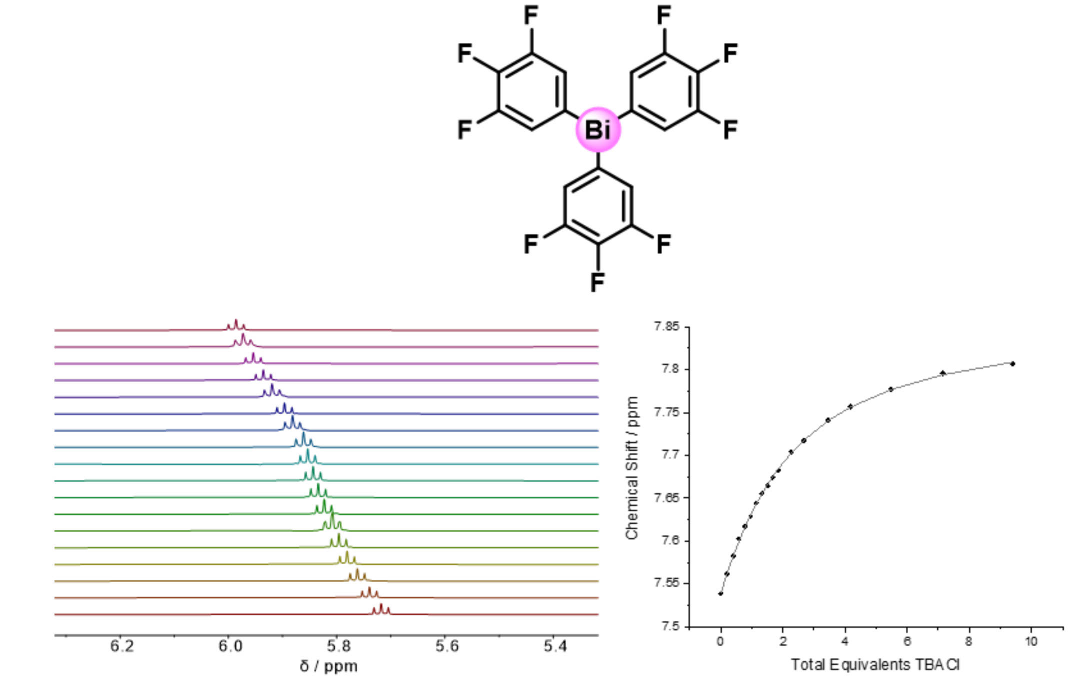

*Figure S39. Stacked ^{1}H-NMR TBACl titration (left) and chloride binding isotherm with dots representing experimental data and solid lines representing the fitted binding isotherm (right) for **1·Bi**^{3F}. Solvent = THF-d_8. T = 298 K.*

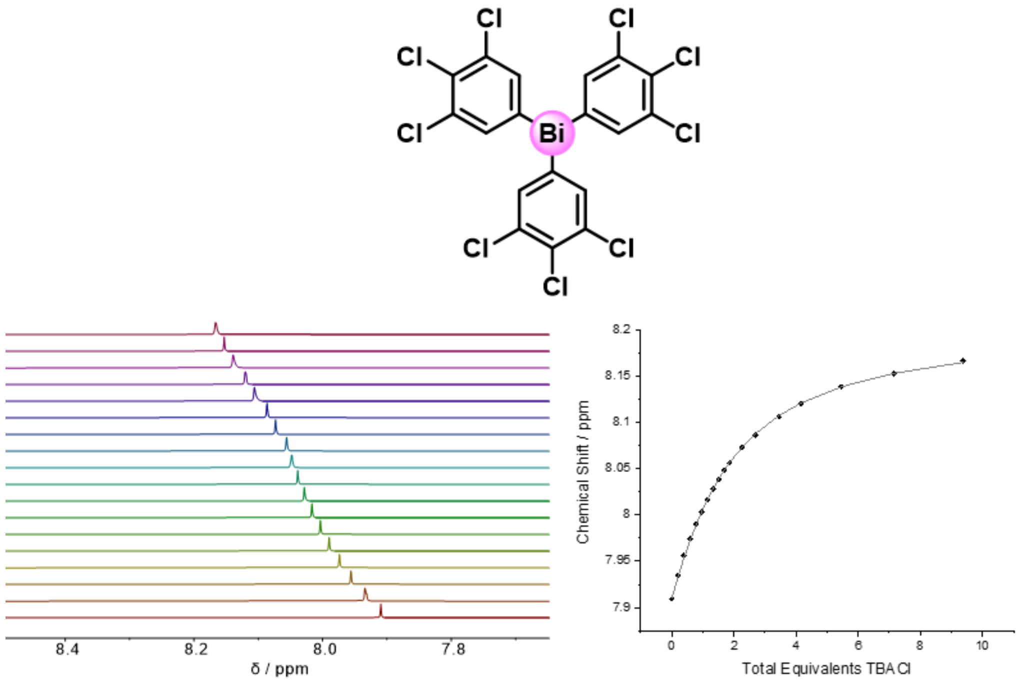

Figure S40. Stacked ^{1}H-NMR TBACl titration (left) and chloride binding isotherm with dots representing experimental data and solid lines representing the fitted binding isotherm (right) for ***1·Bi^{3Cl}****. Solvent = THF-d_8. T = 298 K.*

*Table S4. Anion association constants for **1·Bi^{2CF3}** receptor. Ka(M^{-1}) values calculated using Bindfit software using a 1:1 stoichiometric host-guest binding model. All using TBA salts. Errors (±) are in parentheses. Solvent = THF-d_8. T = 298 K*

**1·Bi^{2CF3}**	***K_a* / M^{-1}**
Cl^-	1300(43)
Br^-	432(8)
I^-	192(2)
OCN^-	344(12)
NO_2^-	332(6)
NO_3^-	124(4)

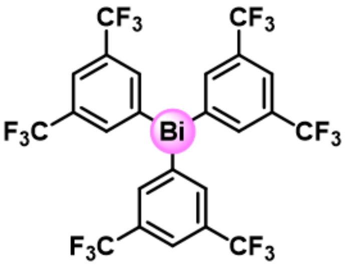

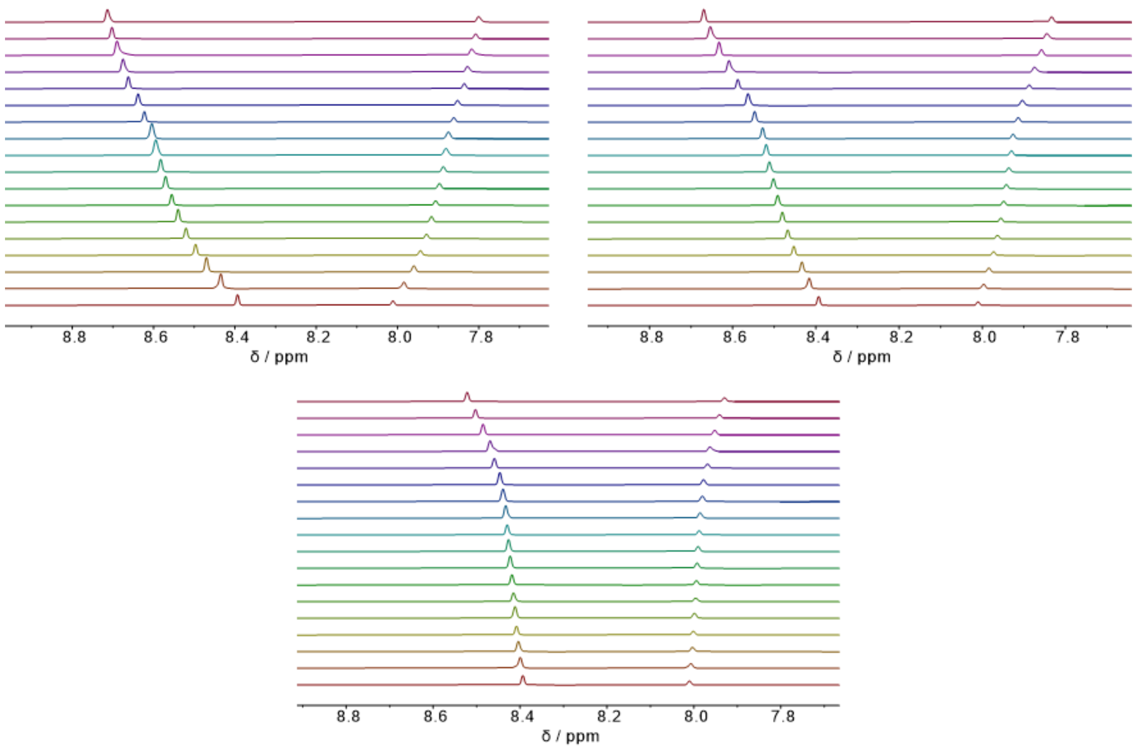

*Figure S41. Stacked ^{1}H-NMR titrations for TBACl (top left), TBABr (top right) and TBAI (bottom) for **1·Bi^{2CF3}**. Solvent = THF-d_8. T = 298 K.*

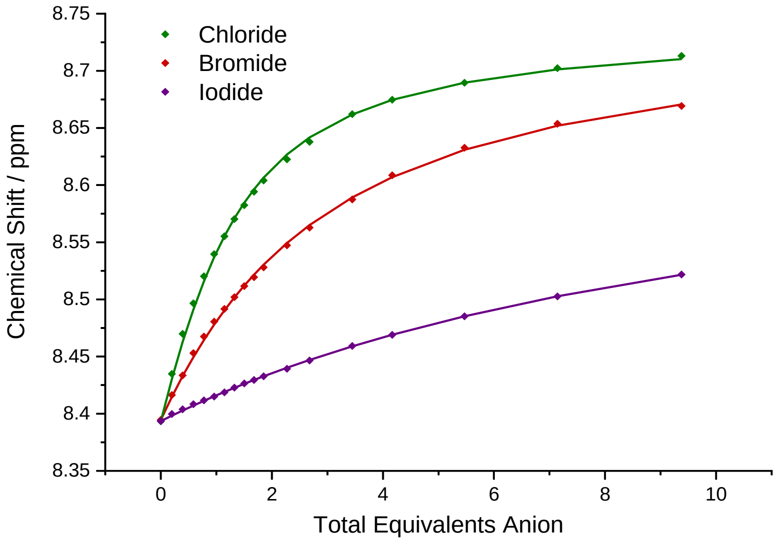

*Figure S42. Binding isotherms for halide anions with dots representing experimental data and solid lines representing the fitted binding isotherm for **1·Bi^{2CF3}***

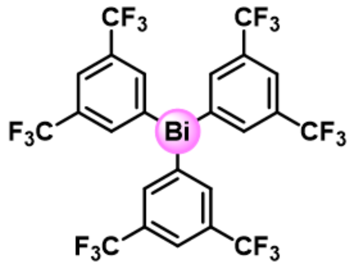

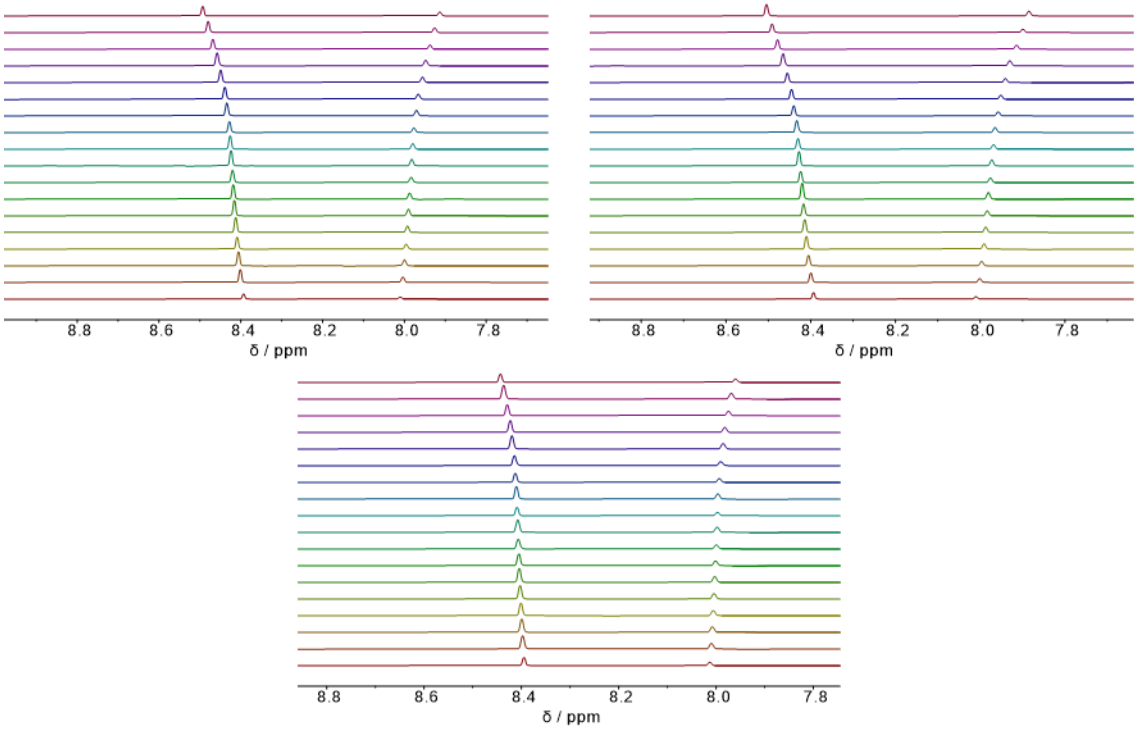

*Figure S43. Stacked ^{1}H-NMR titrations for TBAOCN (top left), TBANO$_2$ (top right) and TBANO$_3$ (bottom) for **1·Bi^{2CF3}**. Solvent = THF-d$_8$. T = 298 K.*

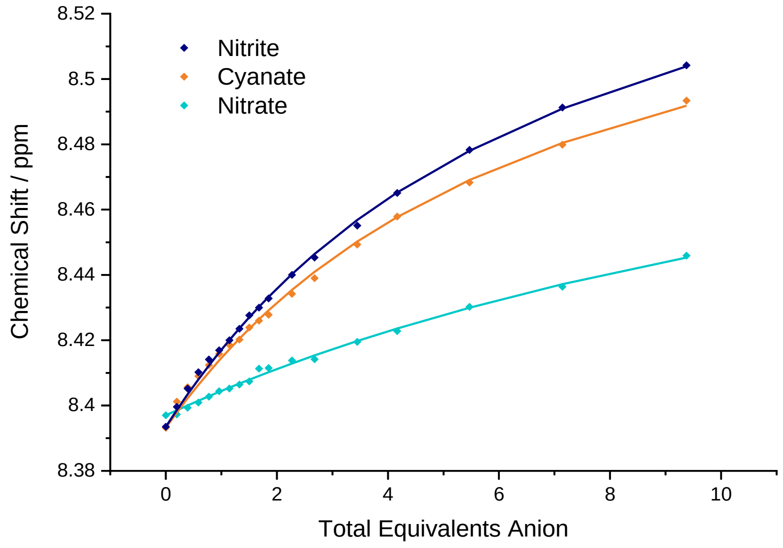

*Figure S44. Binding isotherms for oxo-anions with dots representing experimental data and solid lines representing the fitted binding isotherm for **1·Bi^{2CF3}***

5.3 Antimony Triaryl Compounds

Table S5. Chloride anion association constants for antimony receptors. K_a(M^{-1}) values calculated using Bindfit software using a 1:1 stoichiometric host-guest binding model. Errors (±) are in parentheses. Solvent = THF-d_8. T = 298 K.

	K_a / M^{-1}
**1·Sb^{CN}**	36(2)
**1·Sb^{2F}**	61(2)
**1·Sb^{2Cl}**	52(1)
**1·Sb^{NO2}**	94(2)
**1·Sb^{3F}**	253(3)
**1·Sb^{3Cl}**	341(6)

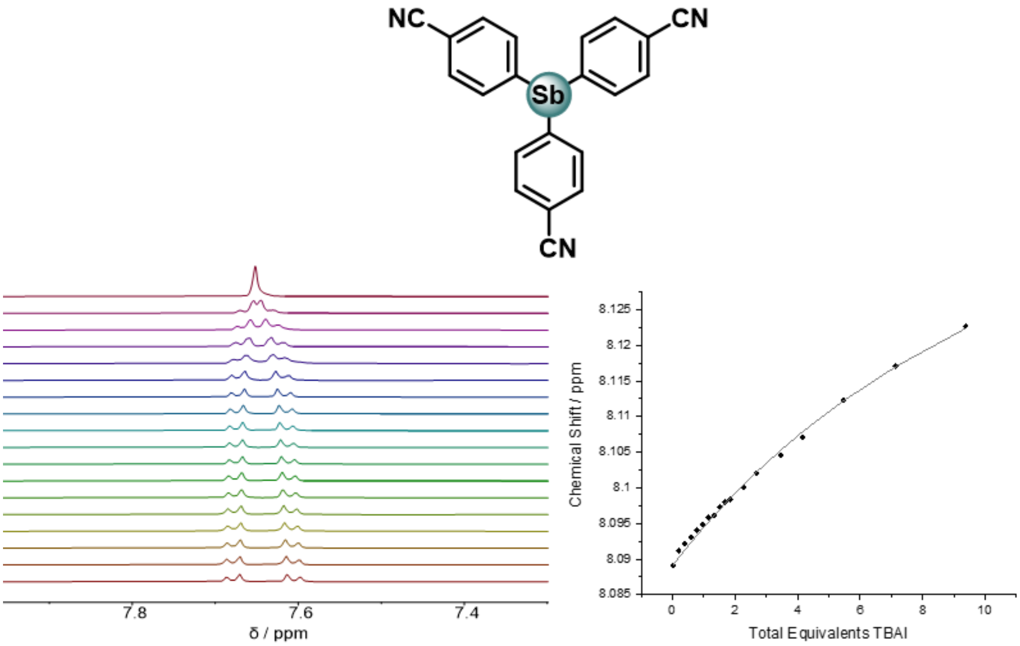

*Figure S45. Stacked ¹H-NMR TBACl titration (left) and chloride binding isotherm with dots representing experimental data and solid lines representing the fitted binding isotherm (right) for **1·Sb^{CN}**. Solvent = THF-d_8. T = 298 K.*

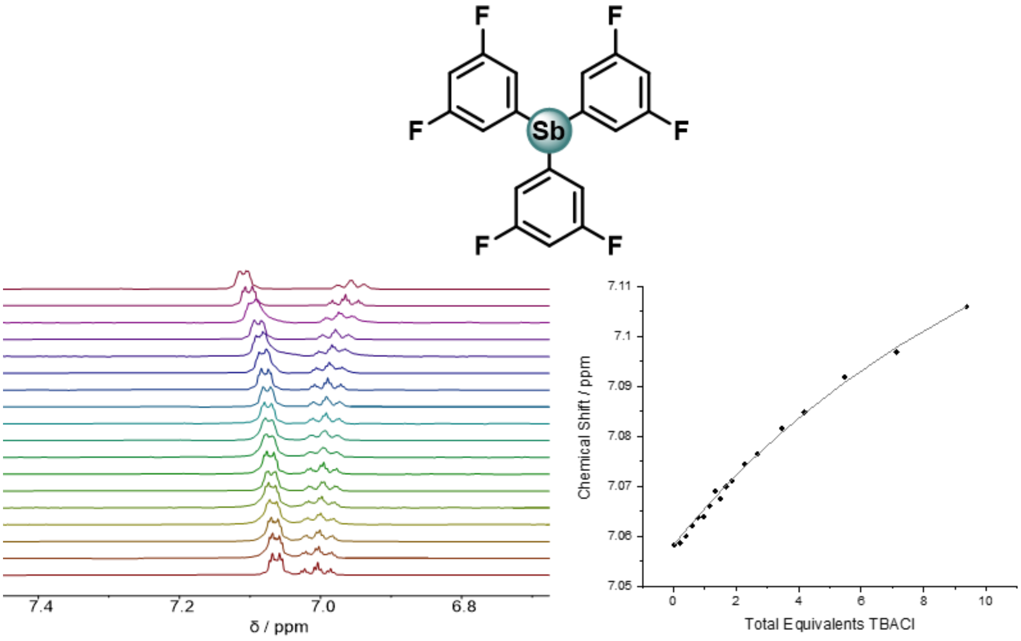

*Figure S46. Stacked ¹H-NMR TBACl titration (left) and chloride binding isotherm with dots representing experimental data and solid lines representing the fitted binding isotherm (right) for **1·Sb^{2F}**. Solvent = THF-d_8. T = 298 K.*

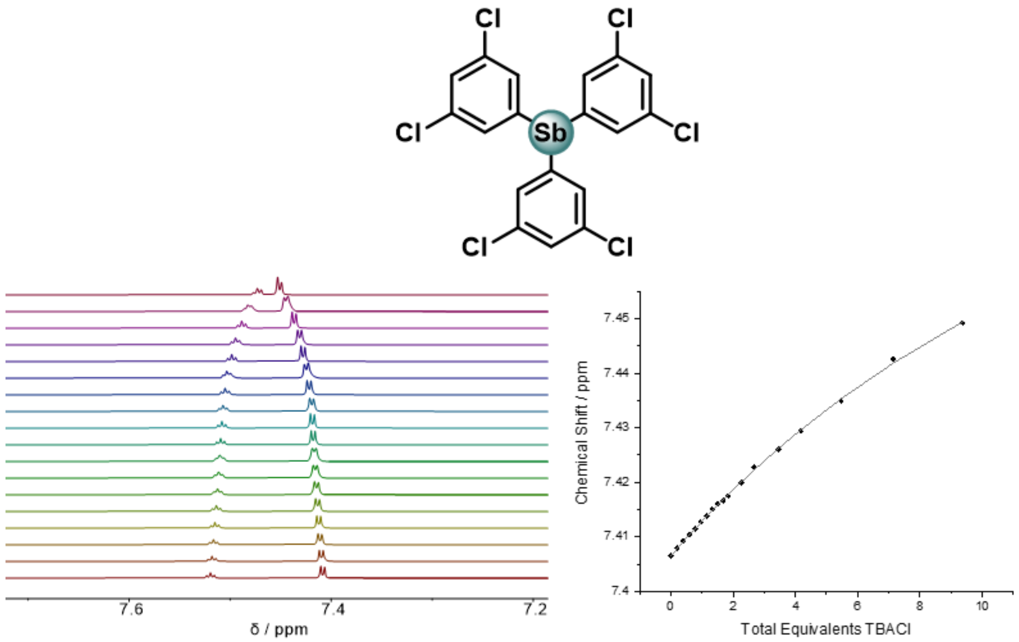

*Figure S47. Stacked ^{1}H-NMR TBACl titration (left) and chloride binding isotherm with dots representing experimental data and solid lines representing the fitted binding isotherm (right) for **1·Sb^{2Cl}**. Solvent = THF-d_8. T = 298 K.*

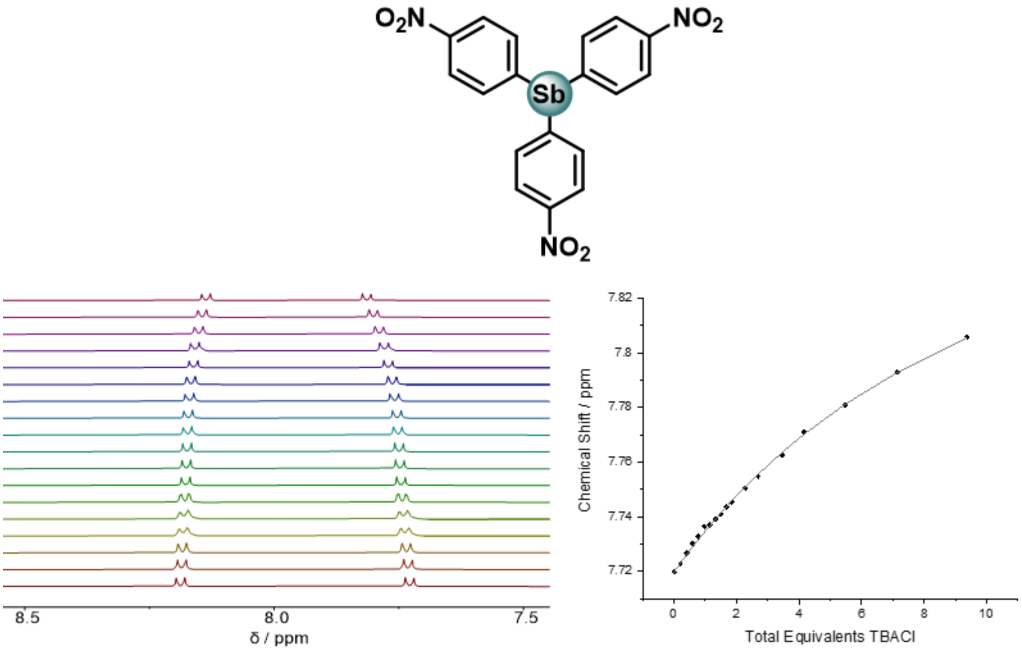

*Figure S48. Stacked ^{1}H-NMR TBACl titration (left) and chloride binding isotherm with dots representing experimental data and solid lines representing the fitted binding isotherm (right) for **1·Sb^{NO2}**. Solvent = THF-d_8. T = 298 K.*

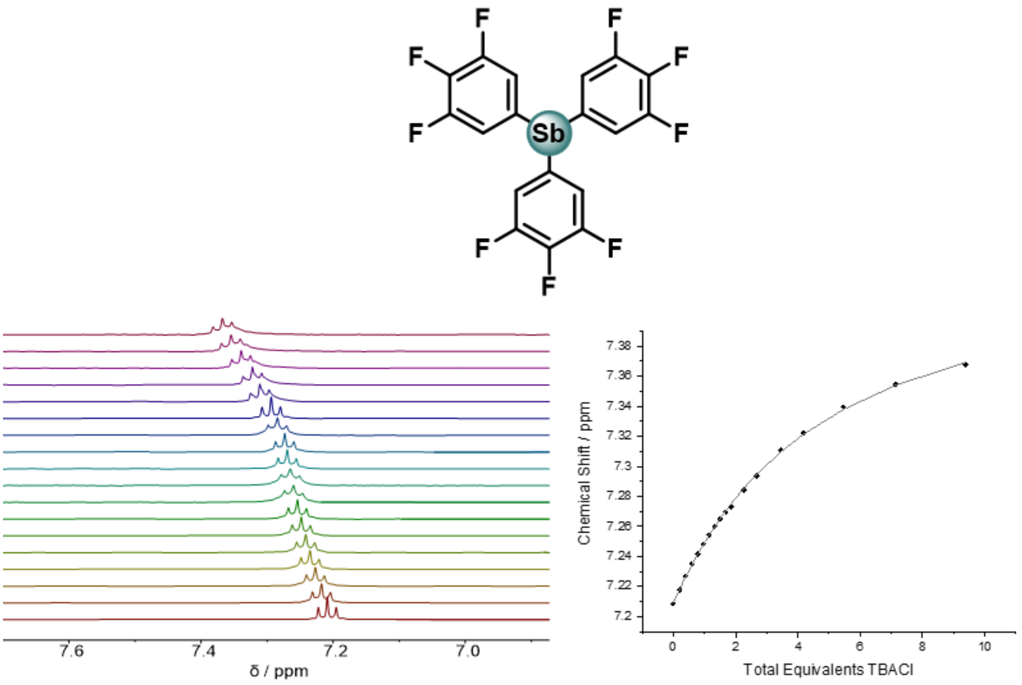

*Figure S49. Stacked ^{1}H-NMR TBACl titration (left) and chloride binding isotherm with dots representing experimental data and solid lines representing the fitted binding isotherm (right) for **1·Sb^{3F}**. Solvent = THF-d_8. T = 298 K.*

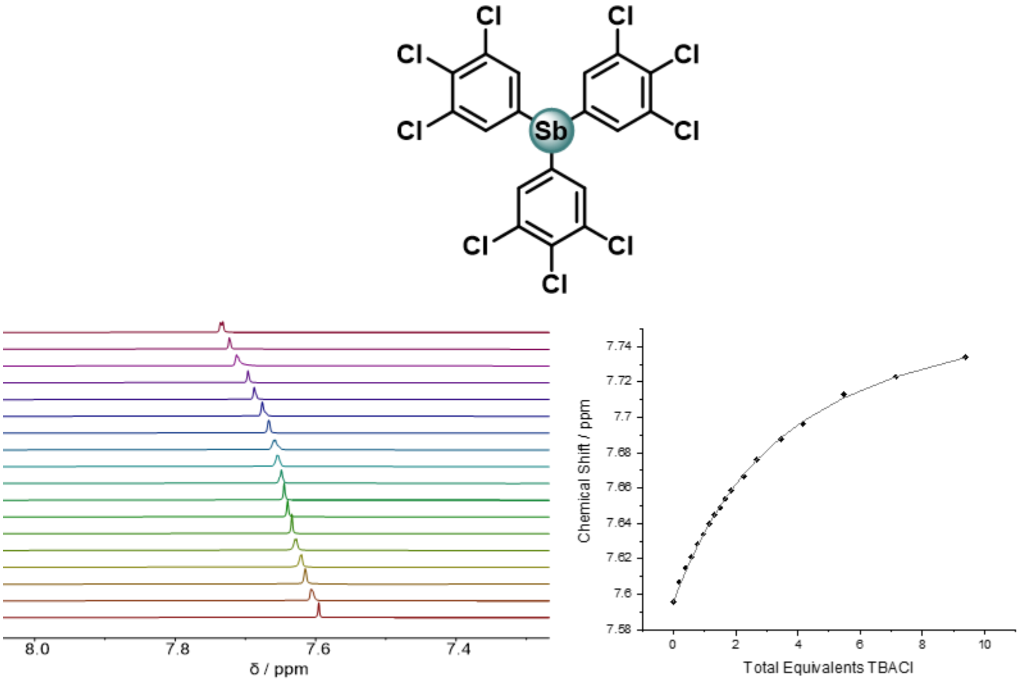

*Figure S50. Stacked ^{1}H-NMR TBACl titration (left) and chloride binding isotherm with dots representing experimental data and solid lines representing the fitted binding isotherm (right) for **1·Sb^{3Cl}**. Solvent = THF-d_8. T = 298 K.*

Table S6. Anion association constants for ***1·Sb***$^{\mathit{2CF3}}$ *receptor.* $K_a(M^{-1})$ *values calculated using Bindfit software using a 1:1 stoichiometric host-guest binding model. All using TBA salts. Errors (±) are in parentheses. Solvent = THF-*d_8*. T = 298 K*

**1·Sb^{2CF3}**	K_a **/ M^{-1}**
Cl^-	702(16)
Br^-	207(3)
I^-	147(4)
CH_3OCO^-	268(15)
OCN^-	263(11)
NO_2^-	125(4)
NO_3^-	79(3)

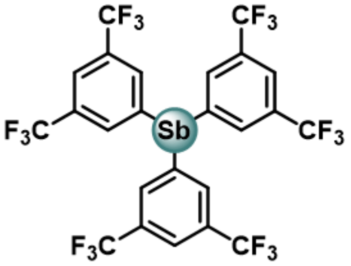

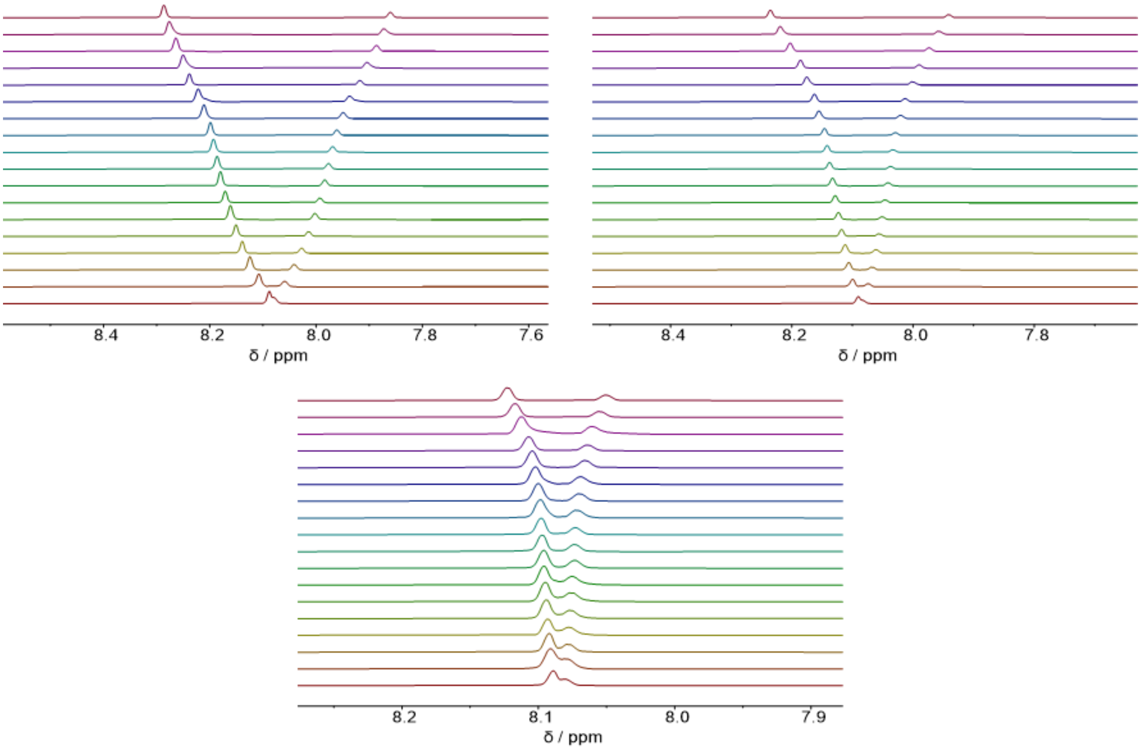

Figure S51. Stacked ^{1}*H-NMR titrations for TBACl (top left), TBABr (top right) and TBAI (bottom) for* ***1·Sb***$^{\mathit{2CF3}}$*. Solvent = THF-*d_8*. T = 298 K.*

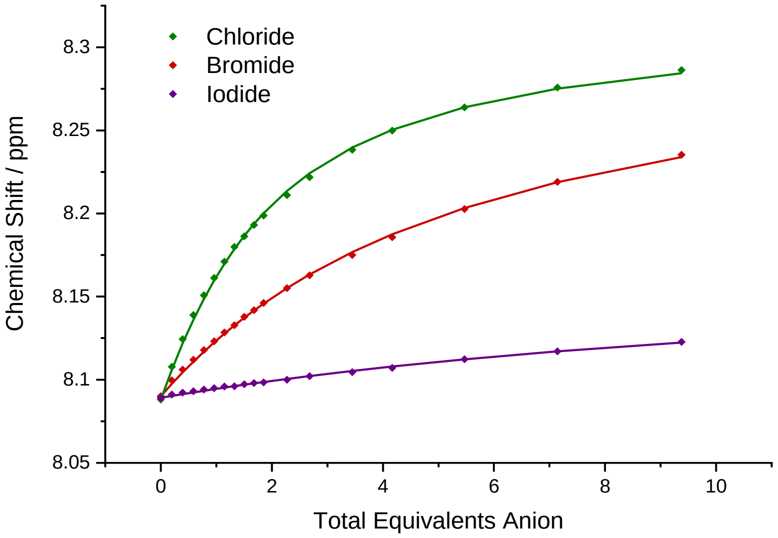

*Figure S52. Binding isotherms for halide anions with dots representing experimental data and solid lines representing the fitted binding isotherm for **1·Sb**^{2CF3}. Solvent = THF-d$_8$. T = 298 K.*

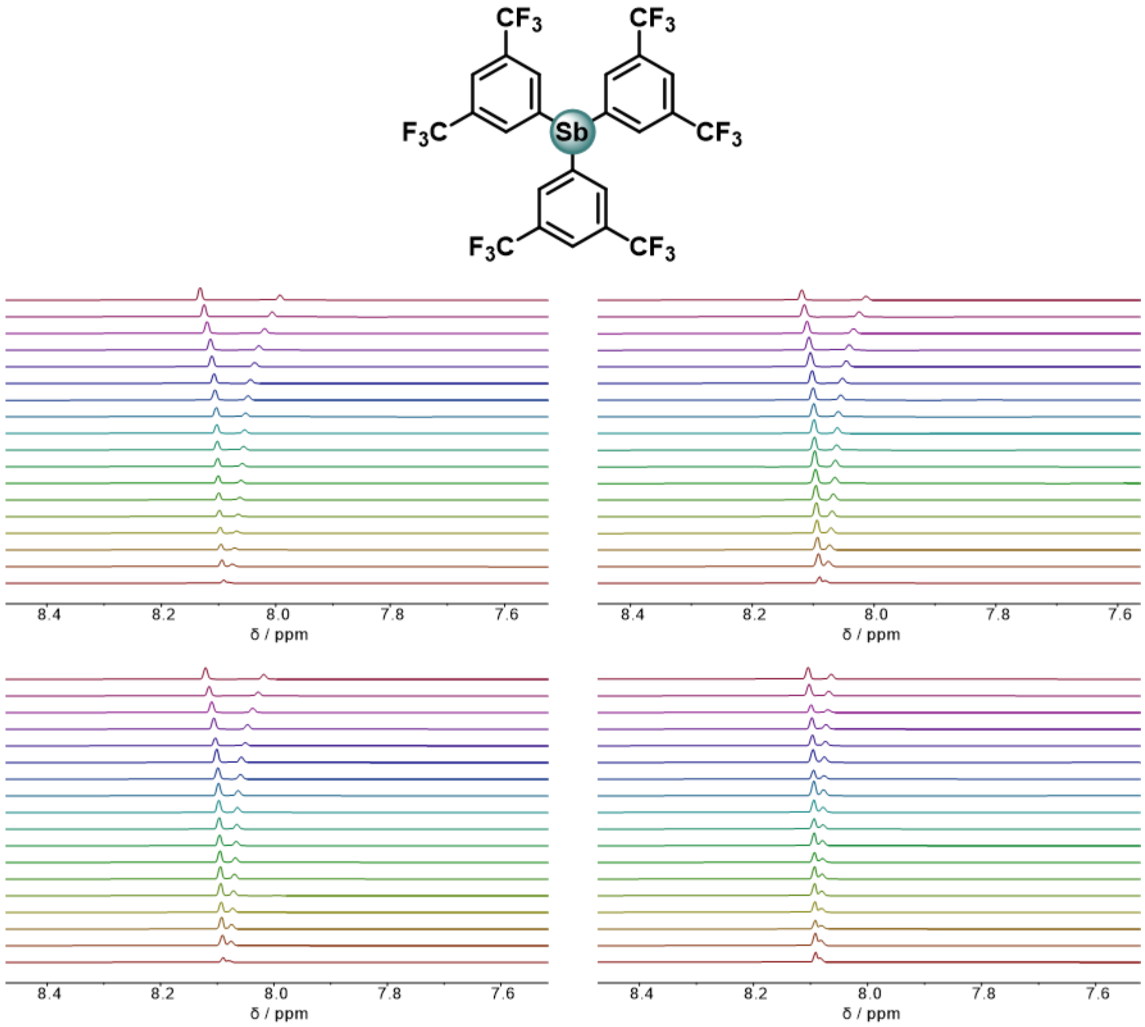

*Figure S53. Stacked ^{1}H-NMR titrations for TBAOCOCH$_3$ (top left), TBAOCN (top right), TBANO$_2$ (bottom left) and TBANO$_3$ (bottom right) for **1·Sb**^{2CF3}. Solvent = THF-d$_8$. T = 298 K.*

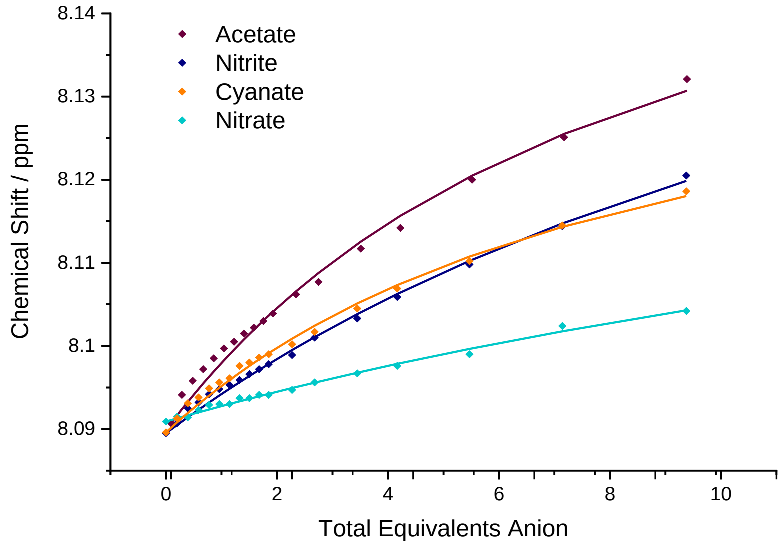

*Figure S54. Binding isotherms for oxo-anions with dots representing experimental data and solid lines representing the fitted binding isotherm for **1·Sb^{2CF3}***

5.4 Pnictogen Tripods

Table S7. Chloride anion association constants for PnB receptors. K_a(M^{-1}) values calculated using Bindfit software using a 1:1 stoichiometric host-guest binding model. Errors (±) are in parentheses. Solvent = THF-d_8. T = 298 K.

	K_a / M^{-1}
**2·Bi^{Bz}**	63(1)
**2·Bi^{PFP}**	115(1)
**2·Sb^{Bz}**	25(1)
**2·Sb^{PFP}**	58(1)

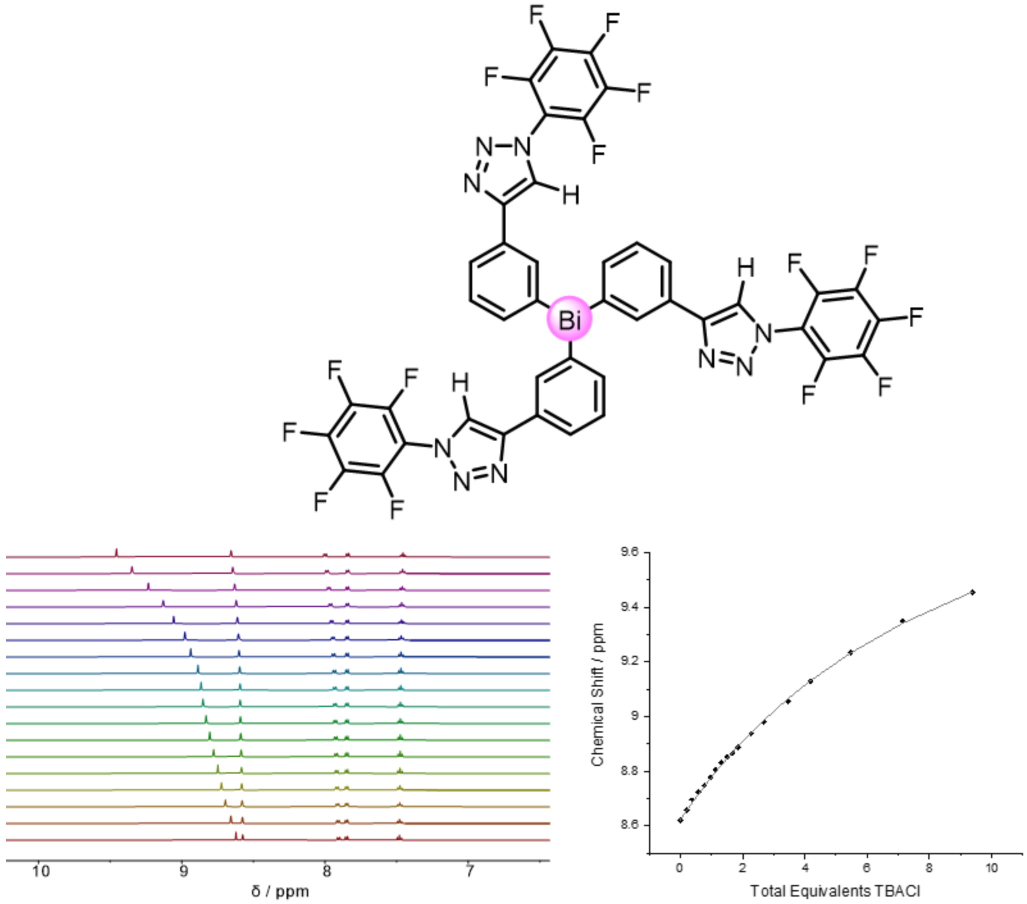

Figure S55. Stacked ^{1}H-NMR TBACl titration (left) and chloride binding isotherm with dots representing experimental data and solid lines representing the fitted binding isotherm (right) for ***2·Bi^{PFP}****. Solvent = THF-d_8. T = 298 K.*

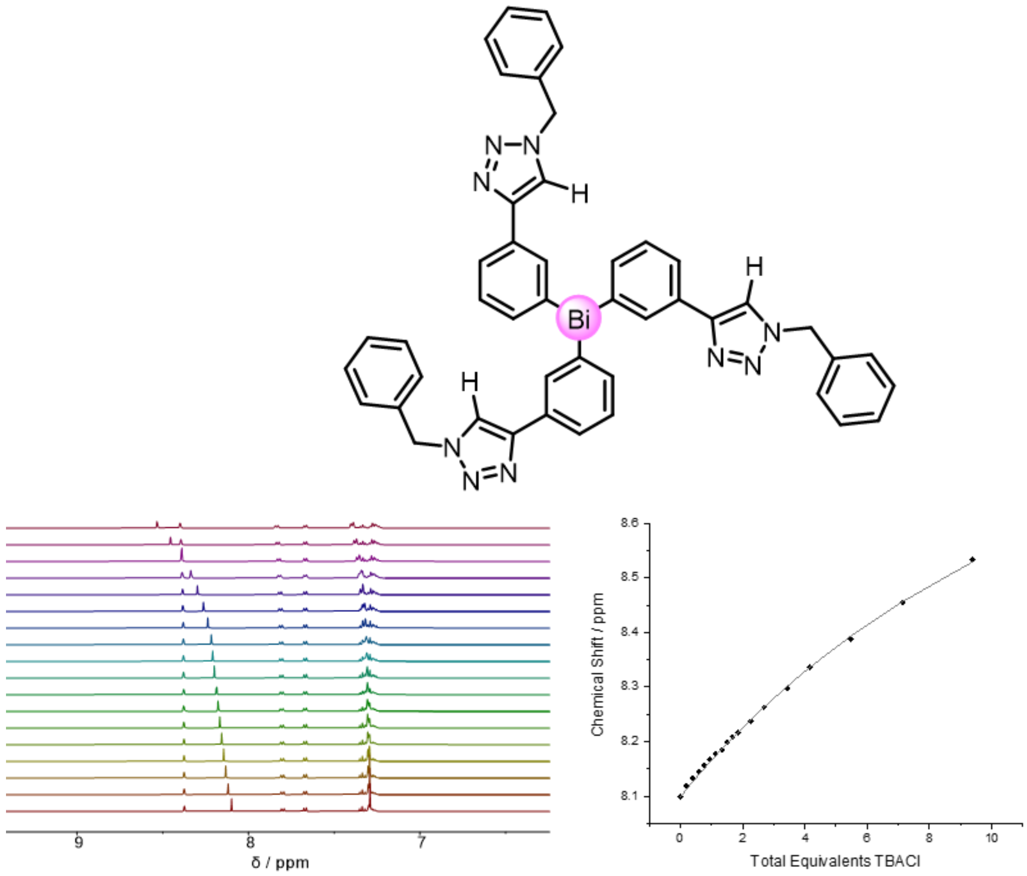

*Figure S56. Stacked ¹H-NMR TBACl titration (left) and chloride binding isotherm with dots representing experimental data and solid lines representing the fitted binding isotherm (right) for **2·Bi^{Bz}**. Solvent = THF-d_8. T = 298 K.*

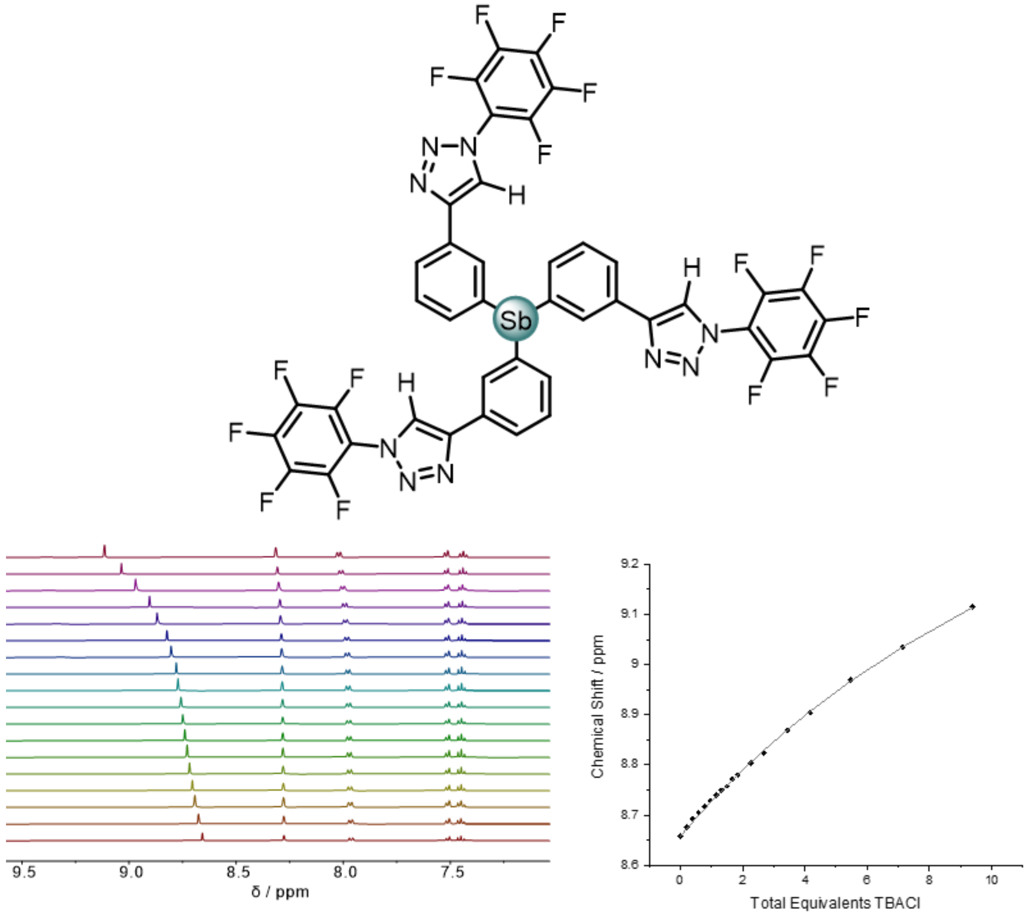

Figure S57. Stacked ^{1}H-NMR TBACl titration (left) and chloride binding isotherm with dots representing experimental data and solid lines representing the fitted binding isotherm (right) for ***2·Sb^{PFP}****. Solvent = THF-d_8. T = 298 K.*

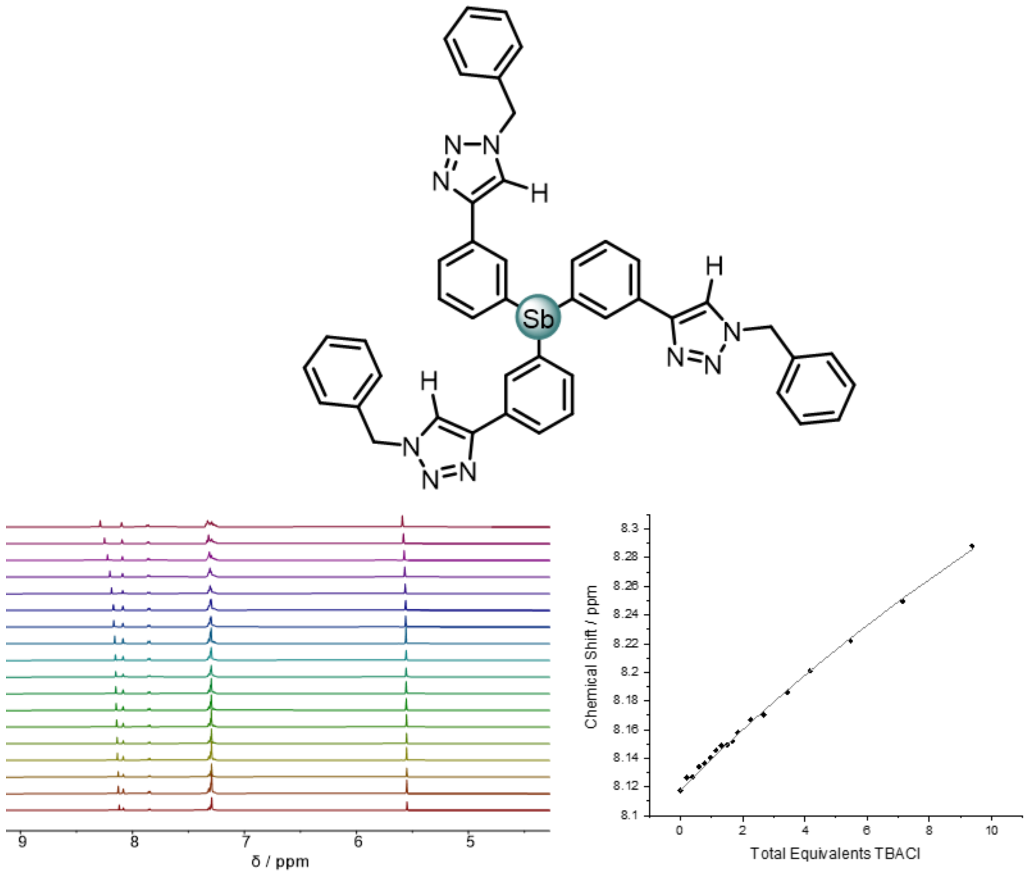

Figure S58. Stacked ^{1}*H-NMR TBACl titration (left) and chloride binding isotherm with dots representing experimental data and solid lines representing the fitted binding isotherm (right) for* ***2·Sb***$^{\mathbf{Bz}}$*. Solvent = THF-d*$_8$*. T = 298 K.*

6 Single Crystal X-Ray Diffraction

Single crystals suitable for X-ray analysis were each coated with Paratone-N oil, mounted on a 200 μm MiTeGen loop and placed in a cold nitrogen stream (150 K) on an Oxford Diffraction Supernova X-ray diffractometer. Diffraction intensities were measured using monochromated Cu Kα diffraction.[13] Data collection, indexing, initial cell refinements, frame integration, final cell refinements and absorption corrections were performed using CrysAlisPro. All structures were solved using the CRYSTALS suite using direct methods and refined against F^2.[14, 15] Hydrogen atoms were included into the model at geometrically calculated positions and refined using a riding model. Figures of the crystal structures were created using the open-source PyMOL Molecular Graphics System (version 2.4.0a0. Schrödinger LLC.). PLATON SQUEEZE was used for the refinement of **2·Sb$^{\mathbf{PFP}}$** in order to include diffuse electron density arising from disordered solvent molecules, which could not be modelled in a sensible manner.[16, 17]

Crystallographic data has been deposited with the Cambridge Crystallographic Data Centre (CCDC) with deposition numbers 2176082 – 2176089. Selected Crystallographic and Refinement Data for X-ray crystal structures can be found in Tables S8, S9 and S10.

Table S8.Selected Crystallographic and Refinement Data for ***1·Sb^{2Cl}, 1·Sb^{3Cl}*** *and* ***1·Sb^{2CF3}***

**Compound**	**1·Sb^{2Cl}**	**1·Sb^{3Cl}**	**1·Sb^{2CF3}**
**Deposition Number**	2176084	2176087	2176083
**Formula**	$C_{18}H_9Cl_6Sb$	$C_{18}H_6Cl_9Sb$, 0.5(C_6H_{14})	$C_{24}H_9F_{18}Sb$
**Formula Weight**	559.74	706.16	761.05
**a (Å)**	8.6149(1)	8.4841(2)	9.9974(3)
**b (Å)**	12.5668(1)	11.9389(3)	12.0314(3)
**c (Å)**	18.5007(2)	13.5490(3)	12.6972(3)
α **(°)**	90	76.9550(19)	112.999(2)
β **(°)**	91.6699(10)	72.2075(19)	104.312(2)
γ **(°)**	90	78.8838(19)	101.518(2)
**Unit cell volume (Å^3)**	2002.07(4)	1261.63(5)	1284.88(7)
**Crystal system**	Monoclinic	Triclinic	Triclinic
**Space group**	P 21/c	P -1	P -1
**Z**	4	2	2
**Temperature (K)**	150 K	150 K	150 K
**Radiation Type**	Copper	Copper	Copper
**λ (Å)**	1.54180	1.54180	1.54184
**Reflections (all)**	22187	26533	27155
**Reflections (unique)**	4157	5216	5337
**R_{int}**	0.031	0.037	0.043
**R[I > 2σ(*I*)]**	0.0774	0.0743	0.0300
**wR(*F*^{2}) (all)**	0.1714	0.1711	0.0428
**S**	1.106	1.107	1.026

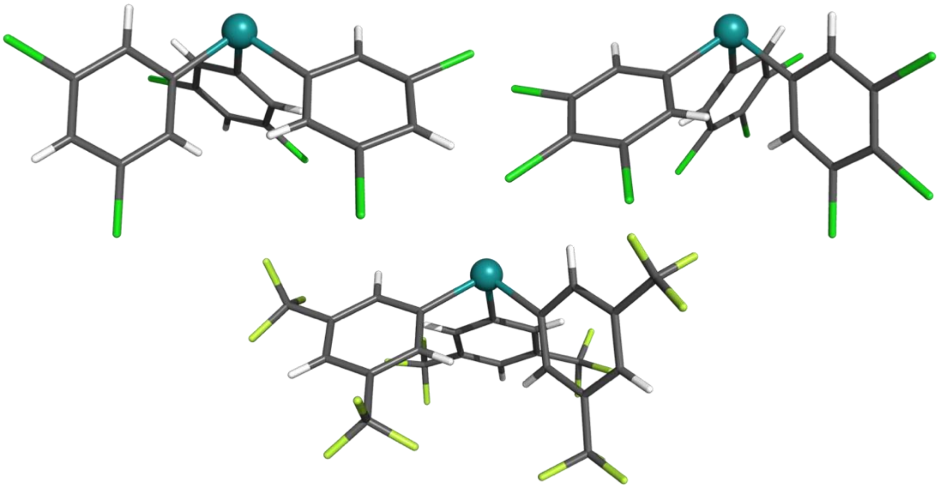

Figure S59. Solid state structures of ***1·Sb^{2Cl}*** *(top-left),* ***1·Sb^{3Cl}*** *(top-right)* ***and 1·Sb^{2CF3}*** *(bottom). Grey=carbon, white=hydrogen, green=chlorine, light green=fluorine, teal=antimony. Solvent omitted for clarity. Fluorine atoms shown for* ***1·Sb^{2CF3}*** *are the ones with the highest occupancy level if disordered over multiple positions.*

*Table S9. Selected Crystallographic and Refinement Data for **1·Bi^{2Cl}**, **1·Bi^{3Cl}** and **1·Bi^{2CF3}***

**Compound**	**1·Bi^{2Cl}**	**1·Bi^{3Cl}**	**1·Bi^{2CF3}**
**Deposition Number**	2176085	2176088	2176082
**Formula**	C_{18} H_9 Bi Cl_6	C_{18} H_6 Bi Cl_9	C_{24} H_9 F_{18} Sb
**Formula Weight**	646.97	750.30	761.05
**a (Å)**	8.5220(1)	6.9675(1)	9.9974(3)
**b (Å)**	12.7365(1)	13.4211(1)	12.0314(3)
**c (Å)**	18.5919(1)	23.0046(2)	12.6972(3)
α **(°)**	90	90	112.999(2)
β **(°)**	92.2593(4)	92.4054(8)	104.312(2)
γ **(°)**	90	90	101.518(2)
**Unit cell volume (Å^3)**	2016.40(3)	2149.30(4)	1284.88(7)
**Crystal system**	Monoclinic	Monoclinic	Triclinic
**Space group**	P 21/c	P 21/n	P -1
**Z**	4	4	2
**Temperature (K)**	150 K	150 K	150 K
**Radiation Type**	Copper	Copper	Copper
**λ (Å)**	1.54184	1.54184	1.54184
**Reflections (all)**	48717	11190	27155
**Reflections (unique)**	4197	4427	5337
**R_{int}**	0.046	0.034	0.043
**R[I > 2σ(*I*)]**	0.0748	0.0560	0.0300
**wR(*F*^{2}) (all)**	0.1824	0.1447	0.0428
**S**	1.132	1.035	1.026

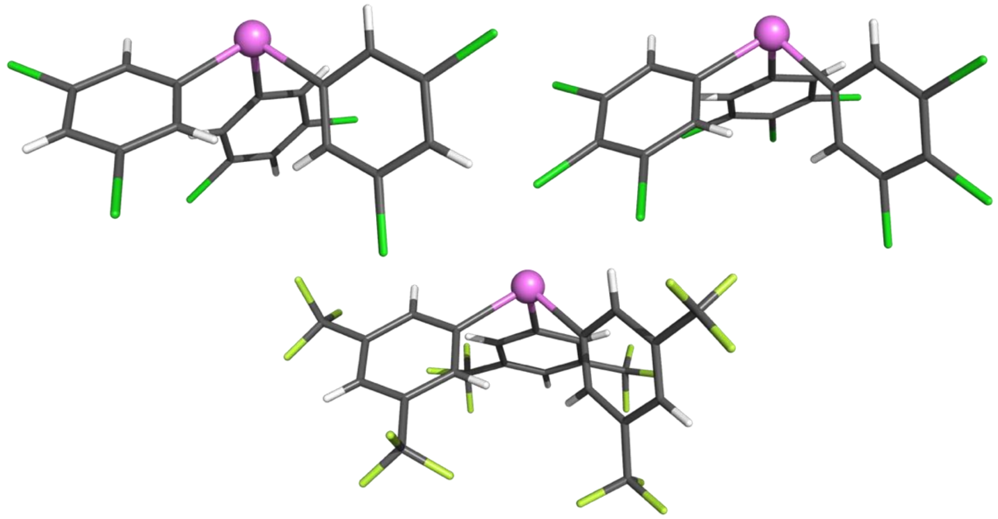

*Figure S60. Solid state structures of **1·Bi^{2Cl}** (top-left), **1·Bi^{3Cl}** (top-right) **and 1·Bi^{2CF3}** (bottom). Grey=carbon, white=hydrogen, green=chlorine, light green=fluorine, pink=bismuth. Fluorine atoms shown for **1·Bi^{2CF3}** are the ones with the highest occupancy level if disordered over multiple positions.*

*Table S10. Selected Crystallographic and Refinement Data for **Bi** and **Sb** tripods*

**Compound**	**2·Sb^{PFP}**	**2·Bi^{PFP}**
**Deposition Number**	2176089	2176086
**Formula**	C_{42} H_{15} F_{15} N_9 Sb	C_{42} H_{15} Bi F_{15} N_9, C H Cl_3
**Formula Weight**	1052.36	1258.96
**a (Å)**	12.6562(3)	23.5794(4)
**b (Å)**	13.8093(4)	10.0950(2)
**c (Å)**	14.3601(4)	18.7203(3)
***α* (°)**	104.779(2)	90
***β* (°)**	111.288(3)	106.246(2)
***γ* (°)**	97.967(2)	90
**Unit cell volume (Å^3)**	2185.42(12)	4278.14(14)
**Crystal system**	triclinic	monoclinic
**Space group**	P -1	P 21/c
**Z**	2	4
**Temperature (K)**	150 K	150 K
**Radiation Type**	Copper	Copper
**λ (Å)**	1.54184	1.54180
**Reflections (all)**	34742	103371
**Reflections (unique)**	9040	8905
**R_{int}**	0.113	0.064
**R[I > 2σ(*I*)]**	0.0803	0.0298
**wR(*F*^{2}) (all)**	0.2232	0.0790
**S**	1.011	0.990

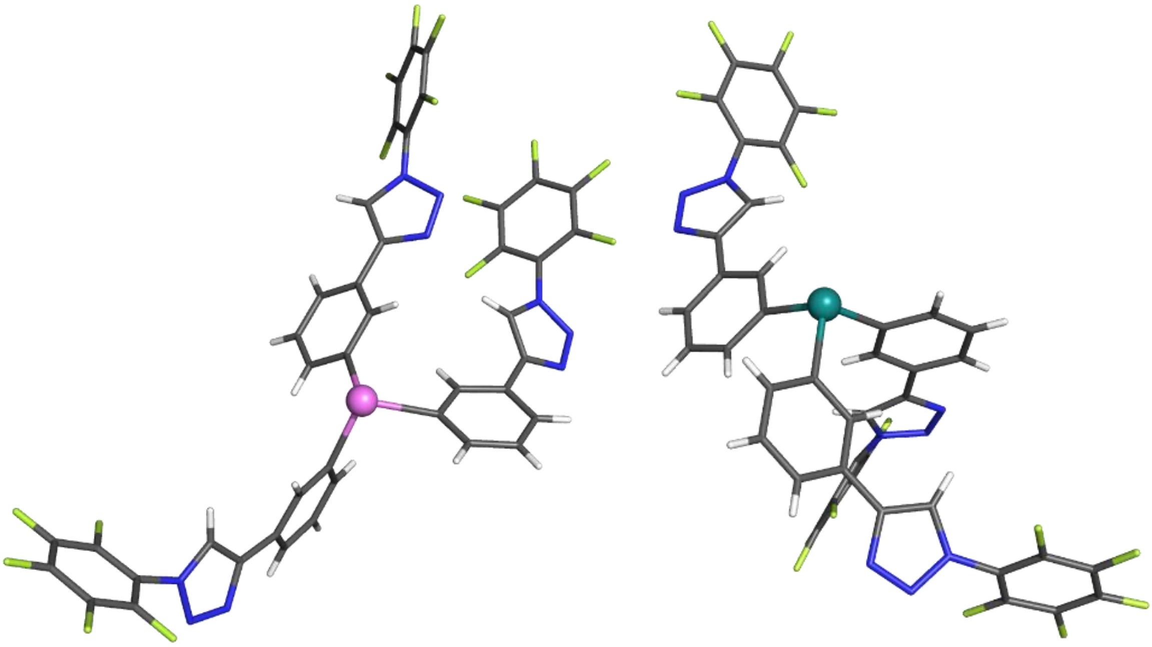

*Figure S61. Solid state structures of **2·Bi^{PFP}** (left) and **2·Sb^{PFP}** (right). Grey=carbon, white=hydrogen, light green=fluorine, teal=antimony, pink=bismuth. Solvent omitted for clarity. Atoms shown for are the ones with the highest occupancy level if disordered over multiple positions.*

7 References

1. T. Murafuji, K. Nishio, M. Nagasue, A. Tanabe, M. Aono and Y. Sugihara, *Synthesis*, 2000, **2000**, 1208-1210.

2. A. F. M. M. Rahman, T. Murafuji, M. Ishibashi, Y. Miyoshi and Y. Sugihara, *Journal of Organometallic Chemistry*, 2005, **690**, 4280-4284.

3. P. Petiot, J. Dansereau and A. Gagnon, *RSC Advances*, 2014, **4**, 22255-22259.

4. J. C. Burnett, C. Lim, B. D. Peyser, L. P. Samankumara, M. Kovaliov, R. Colombo, S. L. Bulfer, M. G. Laporte, A. R. Hermone, C. F. McGrath, M. R. Arkin, R. Gussio, D. M. Huryn and P. Wipf, *Organic & Biomolecular Chemistry*, 2017, **15**, 4096-4114.

5. L.-M. Jin, X. Xu, H. Lu, X. Cui, L. Wojtas and X. P. Zhang, *Angewandte Chemie International Edition*, 2013, **52**, 5309-5313.

6. L. S. Campbell-Verduyn, L. Mirfeizi, R. A. Dierckx, P. H. Elsinga and B. L. Feringa, *Chemical Communications*, 2009, 2139-2141.

7. I. S. Makarov, C. E. Brocklehurst, K. Karaghiosoff, G. Koch and P. Knochel, *Angewandte Chemie International Edition*, 2017, **56**, 12774-12777.

8. A. Gini, M. Paraja, B. Galmés, C. Besnard, A. I. Poblador-Bahamonde, N. Sakai, A. Frontera and S. Matile, *Chemical Science*, 2020, **11**, 7086-7091.

9. S. Yasuike, K. Nakata, W. Qin, M. Matsumura, N. Kakusawa and J. Kurita, *Journal of Organometallic Chemistry*, 2015, **788**, 9-16.

10. M. Yang, D. Tofan, C. H. Chen, K. M. Jack and F. P. Gabbaï, *Angewandte Chemie International Edition*, 2018, **57**, 13868-13872.

11. BindFit v0.5 | Supramolecular, http://app.supramolecular.org/bindfit/.

12. D. Brynn Hibbert and P. Thordarson, *Chemical Communications*, 2016, **52**, 12792-12805.

13. J. Cosier and A. M. Glazer, *Journal of Applied Crystallography*, 1986, **19**, 105-107.

14. P. W. Betteridge, J. R. Carruthers, R. I. Cooper, K. Prout and D. J. Watkin, *Journal of Applied Crystallography*, 2003, **36**, 1487-1487.

15. P. Parois, R. I. Cooper and A. L. Thompson, *Chemistry Central Journal*, 2015, **9**.

16. P. V. Van der Sluis and A. L. Spek, *Acta Crystallographica Section A: Foundations of Crystallography*, 1990, **46**, 194-201.

17. A. L. Spek, *Acta Crystallographica Section C Structural Chemistry*, 2015, **71**, 9-18.
